# Loss of p190A RhoGAP induces aneuploidy and enhances bladder cancer cell migration and invasion by modulating actin dynamics

**DOI:** 10.1038/s41598-025-23687-4

**Published:** 2025-11-18

**Authors:** Qianyu Kang, Xue Kong, Gregoire Najjar, Anca Azoitei, Markus Eckstein, Axel John, Friedemann Zengerling, Felix Wezel, Christian Bolenz, Cagatay Günes

**Affiliations:** 1 Department of Urology, Ulm University Hospital, Helmholtzstr. 10, 89081 Ulm, Germany; 2 Institute of Pathology, Friedrich-Alexander University, Erlangen, Germany; 3 Present Address: Department of Urology, Central Hospital, Shenyang Medical College, Shenyang, 110024 China

**Keywords:** Cancer genetics, Metastasis, Tumour-suppressor proteins, Urological cancer

## Abstract

p190A has been studied across various cancer types, and mutation rates of up to 20% were observed in some cancers, supporting the significance of p190A in carcinogenesis. Since the relevance of p190A in bladder cancer has not been addressed so far, we attempted to explore it. Evaluation of TCGA high-throughput sequencing datasets revealed p190A mutations in up to 8% of bladder cancer samples across several studies. Employing immunohistochemistry on tissue microarray that included 202 BC patient samples, we observed that lower p190A expression correlates with increased invasiveness and poorer survival outcomes. These findings suggested that p190A may have a tumor suppressor function in bladder tissue, consistent with its anticipated function in ploidy-control. p190A knockdown resulted in chromosomal instability in ureter-derived epithelial cells with otherwise normal karyotype, supporting its potential involvement in tumorigenesis. Loss-of-function studies in low-invasive bladder RT4 cell line and gain-of-function experiments in two highly invasive bladder cancer cell lines (T24 and BFTC) demonstrated that p190A influences cell migration and invasion in vitro, as determined by scratch assay and Boyden chamber approaches. This conclusion was further validated by ex vivo porcine bladder invasion approach, invadopodia formation, and gelatin degradation assays. Pathway analysis revealed that altered p190A expression influences both the Rho-ROCK-dependent LIMK1-cofilin pathway and the phosphorylation of cortactin by focal adhesion kinase, both of which regulate critical cellular processes such as actin network organization and polarization to facilitate efficient, coordinated cell migration and division. In summary, our results indicate that p190A has a genuine role in controlling cell-ploidy and regulates actin dynamics in the bladder urothelium, while loss of p190A results in genome instability and drives bladder cancer initiation and progression through deregulated actin dynamics.

**Supplementary Information:**

The online version contains supplementary material available at 10.1038/s41598-025-23687-4.

## Introduction

Bladder cancer (BC) is the most common cancer of the urinary tract and is the 10th most common cancer diagnosed worldwide. With a global incidence and mortality rate of 9.5 and 3.3 per 100,000, respectively, it affects men far more frequently than women^1^. BC is typically classified into non-muscle-invasive bladder cancer (NMIBC) and muscle-invasive bladder cancer (MIBC), with the degree of muscle invasion serving as a critical factor for prognosis^2^. While NMIBC patients generally exhibit favorable prognoses, tumor recurrence is highly common (> 50%), and there is a risk of progression to MIBC (5–20%)^3–6^. In contrast, MIBC is associated with a poorer prognosis due to its rapid metastasis^7^. Another feature that discriminates NMBIC and MIBC is increased aneuploidy in the latter^8^. Aneuploidy is characterized by numerical abnormalities in whole chromosomes and, more recently, by gains or losses in chromosome arms, as highlighted in the cancer genome literature^9,10^. It is a ubiquitous feature of tumor genomes^10,11^. Regarding bladder cancer, there is a positive correlation between aneuploidy and the invasive potential of the disease, with MIBC exhibiting significantly higher aneuploidy levels compared to NMIBC^12^. In fact, increased aneuploidy may serve as a prognostic factor in MIBC^13^.

The remodeling of the cytoskeleton, particularly the actin and microtubule networks, plays a crucial role in tumor cell motility and genome stability. Numerous studies have shown that GTPases play a critical role in controlling their dynamics, and Rho family GTPases are important regulators that aid in processes such cell migration, polarity, and cell cycle progression^14,15^. The dynamic activity of Rho GTPases is suppressed by GTPase-activating proteins (GAPs) and promoted by guanine nucleotide-exchange factors (GEFs)^16^. p190RhoGAP-A (p190A, glucocorticoid receptor DNA-binding factor 1 (GRF-1), or GRLF1, encoded by the *ARHGAP35* gene) is one of the most extensively studied RhoGAPs. It has been considered a primary RhoGAP for RhoA and is implicated in regulating cell migration, polarity, cell division, epithelial differentiation, and cell–cell junctions. These functions are shared with its paralog, p190B RhoGAP (encoded by ARHGAP5), which is also widely expressed and exhibits higher intrinsic RhoGAP enzymatic activity compared to p190A^17^. Interestingly, a ARHGAP5, along with other ARHGAP members (ARHGAP17 and ARHGAP24) were found to be associated with BC^18^. The authors revealed that downregulation of ARHGAP5, ARHGAP17, or ARHGAP24 suppressed BC cell proliferation, migration, and metastasis. On the other hand, p190A has been reported to localize at the cleavage furrow and interact with anillin, indicating its involvement in late mitosis^19,20^. p190A plays a crucial role in regulating RhoA activity at the contractile ring, a structure composed of actin and myosin that drives the final stages of cytokinesis. By acting as a RhoGAP, p190A ensures the timely deactivation of RhoA, allowing for proper contractile ring dynamics and successful abscission. Cells deficient in p190A may encounter difficulties during division, as RhoA GTP promotes enhanced contractility^19^. Experimental evidence suggests that p190A depletion leads to cytokinesis failure, resulting in binucleated or multinucleated cells. Additionally, p190A is essential for the formation of a bipolar spindle in HeLa and RPE cells, functioning independently of its GAP activity^21^.

Given its significant role in cell migration and cell division, dysregulation of p190A has been implicated in various pathological conditions, including cancer progression and developmental disorders^22–26^. Mutations and altered expression levels of p190A have been implicated in various cancers. In endometrial cancer, p190A mutations occur in approximately 20.2% of cases, potentially enhancing Gα₁₃-Rho signaling and contributing to tumor progression^27^. In pancreatic adenocarcinoma, p190A exhibits genetic alterations in about 11% of patients, with copy number variations correlating positively with mRNA expression levels^28^. Gastric cancer tissues often show downregulated p190A expression, which is linked to increased metastasis and poorer patient outcomes^29^. In lung adenocarcinoma, p190A overexpression has been observed, and its knockdown significantly reduces cancer cell viability and invasiveness^17^. These findings underscore the complex role of p190A in cancer, where its mutation or dysregulation can either promote or suppress tumor development depending on the cancer type.

Although high-throughput sequencing datasets revealed p190A mutations in up to 8% of bladder cancer samples across several studies, the specific role of p190A in bladder epithelium and BC remains unexplored. Given its role in actin dynamics and cell motility and based on our previous reports indicating an association between cell-ploidy control and actin dynamics^30,31^ it is intriguing to investigate if p190A contributes to chromosome stability of urothelial cells, and whether p190A has a role in BC progression by influencing the invasive and migratory capacities of BC cells through actin dynamics.

## Materials and methods

### Cell culture

Y235T cells, generously provided by Prof. Jennifer Southgate (University of York, UK), and HBLAK cells, generously provided by Dr. Michele Hoffmann (Department of Urology, Düsseldorf, Germany), were cultured in CnT-Prime medium. HEK293T cells were maintained in DMEM with 10% fetal calf serum (FCS) and 1% penicillin/streptomycin (P/S). T24, UMUC3, J82, and RT4 cell lines were cultured in RPMI1640 with 10% FCS and 1% P/S, while BFTC cells were maintained in DMEM with 20% FCS and 1% P/S. BC61 cells were grown in DMEM F12, supplemented with 10% FCS, 1% P/S, 1% insulin-transferrin-sodium selenite (ITS) medium, and 25 ng/µl basic fibroblast growth factor (bFGF). All cultures were kept at 37 °C in a humidified atmosphere with 5% CO2.

### Transfection and infection

The p190A expression construct on the PCDH-Puro backbone was kindly provided by Dr. S. Huang (Fudan University, Shanghai, China). The lentiviral pZIP-hCMV-ZsGreen-Puro vector containing shRNA targeting human p190A was acquired from transOMIC Technologies Inc., supplied in a bacterial glycerol-preserved format. DNA transfections were conducted using polyethyleneimine (PEI) (Polysciences, Warrington, USA), following the manufacturer’s guidelines. Virus harvesting and cell infections were performed using standard protocols. To generate cell lines with stable target gene expression, cells were cultured under continuous selection with 2.0 µg/ml Puromycin (ThermoFisher Scientific, Waltham, USA).

### Quantitative real-time PCR (RT-qPCR)

Total RNA was extracted using the RNeasy^®^ mini kit (Qiagen, Hilden, Germany) following the manufacturer’s guidelines. For reverse transcription, 100 ng of total RNA was converted into cDNA using the GoScript™ reverse transcription kit (Qiagen, Hilden, Germany). The RT-qPCR experiment was carried out on the Viia7 Real-Time PCR system (Applied Biosystems, Foster City, USA) with a MicroAmp fast optical 96-well reaction plate. Gene expression levels were calculated using the ΔCT method, with GAPDH serving as the reference gene. The primer sequences were as follows (5’ to 3’): for p190A, forward: GTCAGCGGGAACAAGTCTGA, and reverse: GCTCCCGGTCGTTGATTTTG; for GAPDH, forward AAGGTCATCCCTGAGCTGAAC, and reverse ACGCCTGCTTCACCACCTTCT.

### Western blot

Total proteins were extracted using a radio-immunoprecipitation (RIPA) lysis buffer containing protease and phosphatase inhibitors. Following extraction, Western blot analysis was conducted according to standard protocols. The antibodies utilized in the analysis were as follows: Anti-p190A (1:1000, HPA055184, Sigma-Aldrich Chemie GmbH, Steinheim), Anti-RhoA (1:1000, sc-418, Santa Cruz Biotechnology, Inc), Anti-ROCK1 (1:1000, #4035, Cell Signaling Technology, lnc, USA), Anti-LIMK-1 (1:500, sc-28370, Santa Cruz Biotechnology, Inc), Anti-phospho-LIMK1 (Thr508)/LIMK2 (Thr505) (1:1000, #3841, Cell Signaling Technology, lnc, USA), Anti-Cofilin (1:500, sc-376476, Santa Cruz Biotechnology, Inc), Anti- phospho-Cofilin (1:500, sc-271921, Santa Cruz Biotechnology, Inc), Anti-Cortactin (1:500, sc-55579, Santa Cruz, California, USA), Anti- phospho-Cortactin (1:500, #4569, Cell Signaling Technology, lnc, USA), Anti-FAK (1:1000, sc-1688, Santa Cruz Biotechnology, Inc), Anti- phospho-FAK (1:500, sc-81493, Santa Cruz Biotechnology, Inc), Anti-GAPDH (1:10000, MA5-15738, Thermo Fisher Scientific Waltham, Massachusetts, USA). Secondary antibodies, HRP anti-Mouse (1:5000, 7076 S) and HRP anti-Rabbit (1:2000, 7074 S) (Cell Signaling Technology, Danvers, USA).

### Ureter tissue sample UR4-2

For the UR4-2 sample, ureter was collected from a patient, who underwent a nephrectomy at the Urology Department, at the University of Ulm. The studies were conducted with the patients’ written consent and with the local research ethics committee’s approval (239/18). Epithelial cells were collected by mild scraping of the epithelium from ureter tissue surface.

### Tissue microarrays and immunohistochemistry (IHC)

**Fig. 1:**
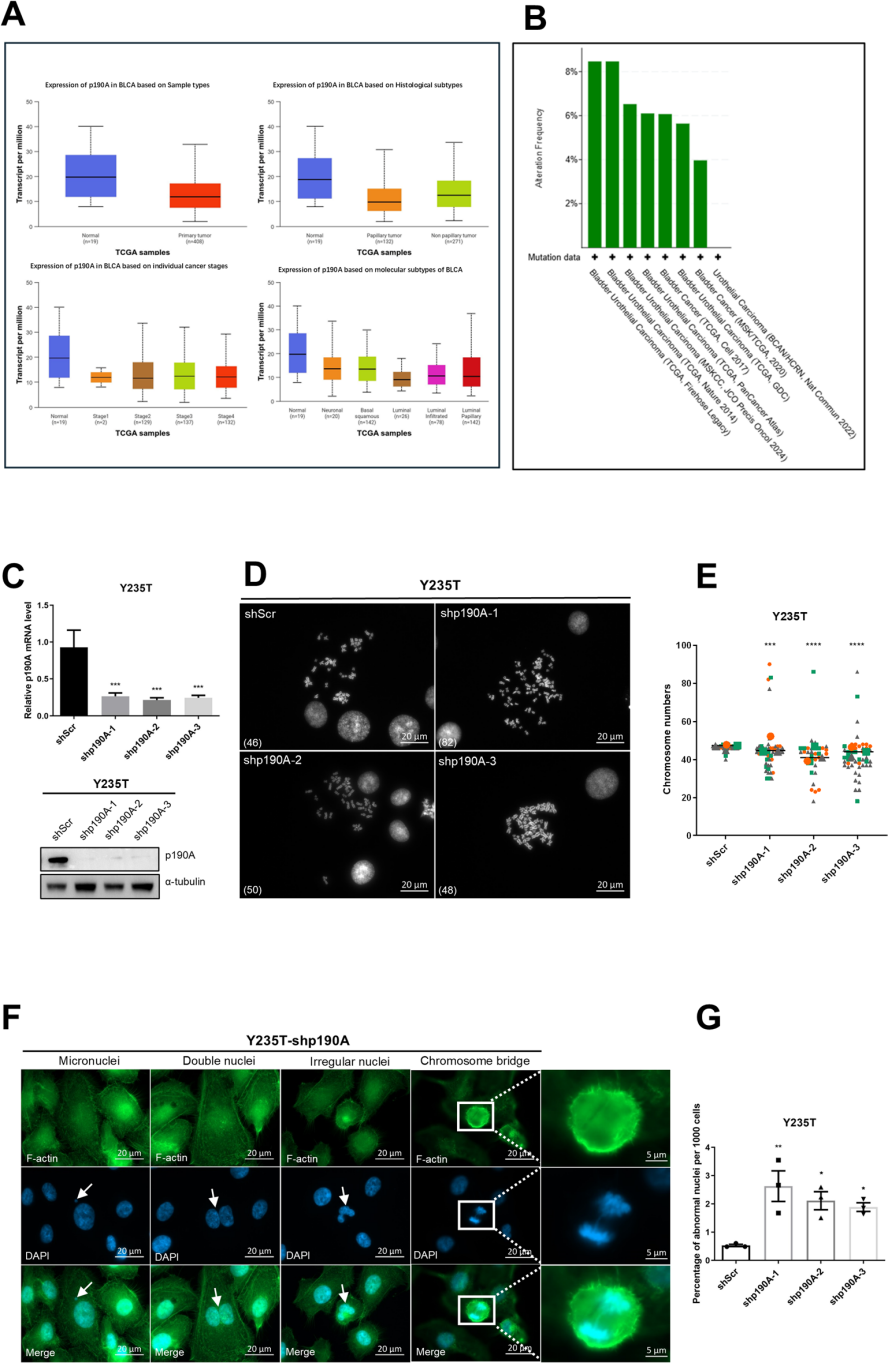
A p190A downregulation induces aneuploidy and genome instability in urothelial cells. p190A expression and genetic alterations in bladder cancer samples. We generated the graphs in A via the UALCAN data analysis portal ^56,57^. The numbers in parentheses indicate the numbers of patients in the respective groups. We generated the graph B via the cBioPortal ^58,59^. **(A)** Expression of *p190A* in normal bladder tissues (*n* = 19) and primary bladder tumour samples (*n* = 408). We indicate the mRNA levels as transcripts per million. Expression of *p190A* in normal bladder tissues and BC tissues based on histological subtypes. Expression of *p190A* in normal bladder tissues and BC tissues based on individual cancer stages. Expression of *p190A* in normal bladder tissues and BC tissues based on molecular subtypes. **(B)** p190A alteration frequency among patients with primary bladder cancer in different urothelial cancer studies. Only studies with more than 100 patients and only patients with point mutations are shown. Green bars represent the mutation rate of samples. **(C)** Relative p190A mRNA (top) and protein (bottom) levels in Y235T scramble control (shScr), shp190A-1, shp190A-2, and shp190A-3 cells. α-tubulin was used as a loading control. Please note that for all Western blots (WB), full-length gels are provided as supplemental data (Suppl. Figure 2). **(D)** Representative metaphase chromosome images and **(E)** quantification of the chromosome numbers in Y235T-shScr and Y235T-shp190A cells. Scale bar, 20 μm. Number in () indicated chromosome number. Data are presented as SuperPlots as described by Lord et al. {Lord, 2020 #1}. Each dot represents an individual cell, color-coded by biological replicate. Large symbols indicate the mean value of each replicate. **(F)** Representative images and **(G)** quantification of abnormal nuclei division in Y235T-shp190A cells. Scale bar, 20 μm and 5 μm. Statistical data are shown as the mean ± SEM. All experiments were performed at least in triplicates (*n* = 3). ANOVA was applied for the statistical analyses. (* *p* < 0.05, ** *p* < 0.01, *** *p* < 0.001, **** *p* < 0.0001).

Two ureteral urothelium samples were sourced from patients who underwent nephrectomy at the Urology Department of Ulm University with local ethical approval (239/18). The tissue microarray comprising BC patient samples was graciously provided by Dr. Markus Eckstein from the Department of Pathology at Erlangen University, Germany, with ethical approval of Friedrich-Alexander-University (3755 and 329_16B). Informed consent was obtained from all patients, and all analyses were performed in accordance with the Declaration of Helsinki. A detailed list of clinical data of patients with BC is provided in Supplementary Table 1. Immunohistochemical (IHC) staining of tissue samples was performed with 3,3’-Diaminobenzidine (DAB) substrate solution (Scytek Laboratories, West Logan, USA) following the manufacturer’s instructions. The expression of p190A was evaluated in 50 pairs of NMIBC and 152 pairs of MIBC samples. The specificity of p190A antibody (1:20, HPA055184, Sigma-Aldrich Chemie GmbH, Steinheim) was confirmed by Western blot experiments, where only one specific protein band is observed (Figs. 1, 3 and 5 and Suppl. Figure 4) in the tissue sections from ex vivo porcine bladder invasion model experiment. IHC staining showed specific p190A staining in the RT4 cells, while staining intensity was reduced in RT4-shp190A cells upon p190A knockdown, supporting the antibody’s specificity (Suppl. Figure 4). The protein expression and staining intensity of arrays were scored as outlined in the Supplementary Tables 2 and 3, respectively. The scores were evaluated by two individuals independently. An average score was derived from duplicate staining of each patient’s samples.

### Chromosomes spread assay

Following the infection and selection of Y235T cells with p190A-targeting shRNA, the cells were cultured to 50–70% confluency and chromosome spread assays to assess the induction of aneuploidy were performed as previously described ^31^. A volume of 120–150 µl of the resulting suspension was dropped onto a clean, ice-cold glass slide from a height exceeding one meter to lyse the cells and achieve an even dispersion of chromosomes on the slide. DAPI mounting medium (ThermoFisher Scientific, Waltham, USA) was applied, followed by a cover slip. The slides were allowed to dry in a dark room overnight before analysis using a Zeiss TCS SP5 confocal microscope equipped with a 100 × objective.

### Immunofluorescence

To detect abnormal nuclei, Y235T-shScr and Y235T-shp190A cells were cultured on coverslips for a minimum of 48 h. After fixation with 4% paraformaldehyde (PFA), the cells were stained with DAPI for visualization of the nuclei and with Phalloidin-iFluor 488 Reagent (1:1000, ab176753, Abcam, Cambridge, UK) to highlight the cell membrane. For cortactin staining, RT4, T24, BFTC cells were incubated with mouse anti-Cortactin primary antibody (1:500, sc-55579, Santa Cruz, California, USA) overnight at 4℃. The following day, cells were treated with an anti-Mouse antibody conjugated with Cy5 (1:200, 115–605-006, ThermoFisher Scientific, Waltham, USA) at room temperature for 1 h. To visualize F-actin, Phalloidin-iFluor 488 or Phalloidin-iFluor647 reagent (1:1000, ab176759, Abcam, Cambridge, UK) was combined with the secondary antibody.

### Wound healing and Boyden chamber assays

The assessment of cell migration was conducted using the Wound healing assay, while cell migration and invasion were evaluated through the Boyden Chamber assay, following previously established protocols ^32^. In wound healing assay, we monitored the cell migration capacity by photography, taking images every 4 h (T24 cells), 6 h (BFTC cells) or 24 h (RT4 cells) using microscopy (Carl Zeiss, Oberkochen, Germany). The wound area at each time point was analyzed using ImageJ software, version 1.53t (https://imagej.net/ij) Wound healing percentage was calculated as the proportion of the closed area relative to the initial wound area. In Boyden chamber assay, the seeded cell number was 2 × 10^4^ /well for T24 and BFTC and 1 × 10^5^ /well for RT4. Images were captured with a 5× objective for the Wound Healing assay and a 10× objective for the Boyden Chamber assay, utilizing a Zeiss TCS SP5 confocal microscope.

### Ex vivo porcine bladder invasion model

The experiments were conducted following the methodology outlined by Wezel et al. ^33^. After a culture period of 2 weeks for T24 cells and 4 weeks for RT4 cells, the tissue samples were fixed in a 10% formalin solution and subsequently embedded in paraffin using a semi-enclosed bench-top tissue processor. The resulting paraffin blocks were sliced into 4-µm thick sections, which were then processed for hematoxylin and eosin (HE) staining and immunohistochemistry (IHC). Both anti-p190A and anti-human leukocyte antigen (HLA) (1:200, ab70328, Abcam, Cambridge, UK) antibodies were used for IHC staining to evaluate the invasiveness of cells into the porcine bladder submucosa. While the signal of p190A staining was relatively weak (Suppl. Figure 4) and making it difficult to evaluate invasion clearly, HLA staining provided a stronger, more distinct signal for human cells within porcine tissue and was therefore used for imaging in the main figures (Fig. 3Q, R).

### Detection and quantification of invadopodia formation

Invadopodia formation is detected and counted through the co-staining of F-actin and cortactin in immunofluorescence (IF). The procedure of IF is described before. DAPI is applied for nuclear staining. Under observation in Zeiss TCS SP5 confocal microscope, the DAPI stain emits a blue fluorescence when excited at a wavelength of 405 nm, while Cortactin exhibits red fluorescence upon excitation at 545 nm and F-actin displays green fluorescence under excitation at 488 nm. The merged yellow dots resulting from the co-localization of Cortactin (red) and F-actin (green) represents invadopodia. We counted the number of yellow dots as the number of invadopodia formation.

### Gelatin degradation assay

This assay was conducted according to the protocol established by Diaz ^34^. In this procedure, 3 × 10^4^ RT4 cells or 1 × 10^4^ T24 and BFTC cells were plated on coverslips coated with Gelatin-FITC. Analysis using confocal microscopy was performed with a 40× objective. The degradation area was quantified using the ImageJ software, and the normalized area was determined by dividing the measured area by the total cell area in the entire image.

### Zymography

To evaluate matrix metalloproteinase (MMP) activity, gelatin and collagen zymography assays were performed using the conditioned media (supernatants) from RT4 and BFTC cells with p190A knockdown or overexpression. Briefly, equal amounts of cell culture supernatants were collected and subjected to SDS-PAGE in gels containing either gelatin or collagen as substrates. For collagen zymography, additionally whole cell lysates (WCL) were also used. After electrophoresis, gels were renatured, incubated in developing buffer to allow proteolytic digestion, and stained with Coomassie Blue. Clear bands represent areas of substrate degradation, indicating MMP enzymatic activity.

### Statistics analysis

GraphPad Prism 8 was used to create graphical representations and conduct statistical analysis. The Student’s t-test was applied for group comparisons. An ANOVA was used, either one-way or two-way, when comparing three or more groups. A 95% confidence interval was used to determine statistical significance, and a p-value of less than 0.05 was considered statistically significant.

## Results

### p190A downregulation induces aneuploidy and genome instability in urothelial cells

p190A has been studied across various cancer types, but the potential function of p190A in bladder tissue and BC nor its role in ploidy-control have been explicitly discussed in prior research. We searched The Cancer Genome Atlas (TCGA) resources for available information on the expression of p190A in normal and cancer tissues and for p190A mutations in cancer samples. Firstly, using the ualcan web resource (http://ualcan.path.uab.edu) for analyzing TCGA sequencing results, we found that p190A expression is often lower in bladder cancer samples compared to normal bladder samples, especially in papillary tumors of luminal origin (Fig. 1A). Moreover, several studies revealed a high mutation rate for *ARHGAP35*, the gene that encodes p190A, ranging from 4% to over 8% of tumor samples (http://www.cbioportal.org) (Fig. 1B).

Firstly, we investigatated the role of p190A in maintaining genome stability in uroepithelial cells. For this purpose, we used three distinct shRNAs to suppress p190A expression in Y235T cells, a telomerase-immortalized cell strain derived from human ureter epithelium with otherwise normal karyotype. The knockdown efficiency was confirmed by RT-qPCR and Western blot analyses, showing a significant reduction in p190A expression at both the mRNA and protein levels compared to cells transduced with a scrambled shRNA (Fig. 1C). Subsequently, metaphase spread assay was conducted to count chromosome numbers, demonstrating a marked increase in aneuploidy in Y235T cells with p190A knockdown (Fig. 1D and E). Chromosome counts varied from 30 to 90 in Y235T- shp190A-1 and from 18 to 86 in Y235T-shp190A-2 and Y235T- shp190A-3. As expected, Y235T-shScr cells infected with a control shRNA vector preserved a normal karyotype in the majority of control cells (Fig. 1E). Defects in ploidy control are also known to be characterized by aberrant nuclear shapes, including micronuclei, double nuclei, irregular nuclei, and the existence of chromosome bridges during cell division. By utilizing cytoskeleton labeling with Anti-F-actin and DAPI staining for DNA, we were able to quantify these anomalies. By counting ~ 1,000 cells for each group, we observed that p190A knockdown led to an increase of abnormal nuclei in Y235T-shp190A cells compared to Y235T-shScr cells (0.5% in shScr vs. 2.6% in Y235T-shp190A-1, *p* < 0.01, 2.1% in Y235T-shp190A-2, *p* < 0.05 and 1.9% in Y235T-shp190A-3, *p* < 0.05; Fig. 1F and G). These findings reveal that p190A plays a critical role in maintenance of genome stability in human ureter epithelial cells and that alterations in p190A expression or function may play a role in the initiation of BC.

### Low p190A levels associate with BC progression and correlate with poor survival outcome

**Fig. 2:**
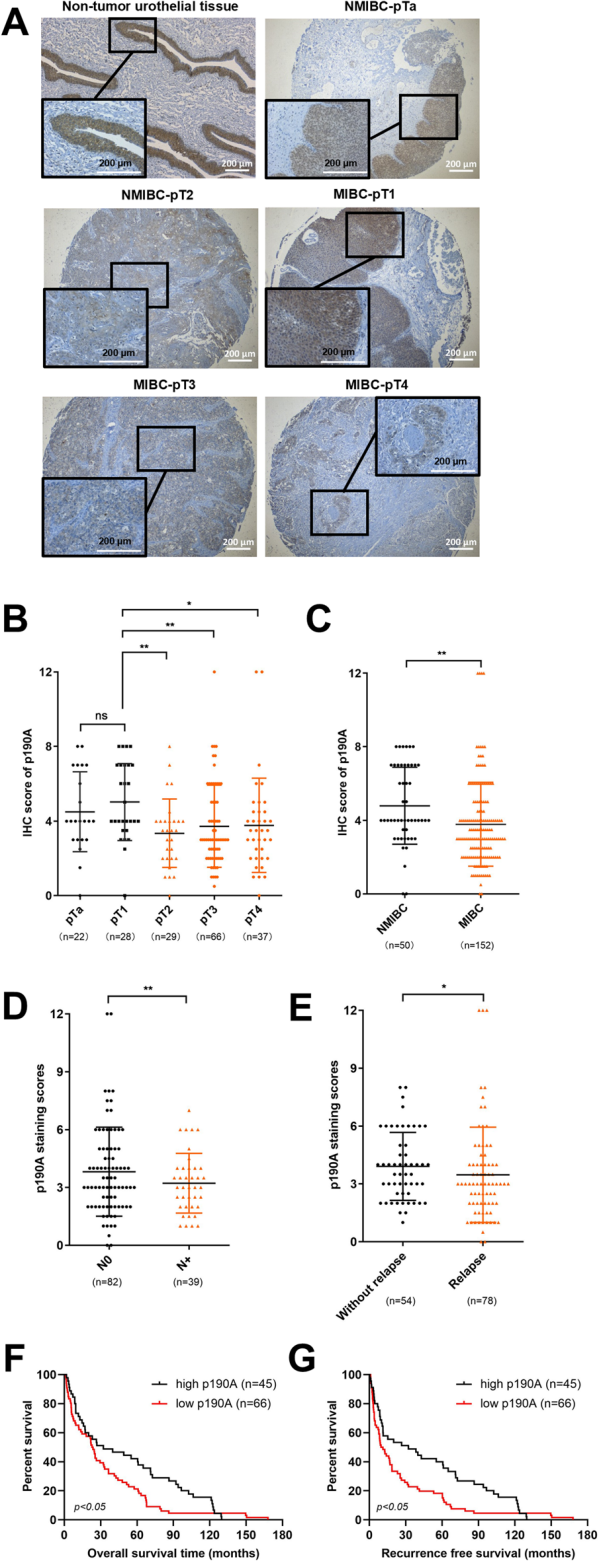
A p190A expression is decreased in high-invasive BC and correlates with poor prognosis. **(A)** Representative immunohistochemical (IHC) images of p190A in non-tumor urothelial tissue and a tissue microarray of bladder cancer (BC) samples with different pathological stages. Scale bar, 200 μm. **(B**,** C)** Statistics of IHC scores of p190A in non-muscle invasive bladder cancer (NMIBC) (pTa + pT1) and MIBC (pT2 + pT3 + pT4) patients from tissue microarrays. Statistics of IHC scores of p190A in MIBC patients with/without lymph node metastasis **(D)** and with/without relapse **(E)** from tissue microarrays. Kaplan–Meier survival curves of overall survival **(F)** and recurrence free survival **(G)** based on p190A expression in the MIBC patients. Evaluation of p190A immunoreactive (IRS) scores is calculated by the product defined by staining intensity scores x and the number of the positive cells scores as described in the Supplementary Tables 2 and 3. Based on the average p190A score across all patient samples, we set an IHC staining score of 4 as cutoff, classifying expression levels as high (≥ 4) and low (< 4). Please note that 111 out of the 152 patients with MIBC were included for the survival analyses. Patients classified as having secondary bladder cancer, as these likely represent non-initial diagnoses, including possible recurrences, second primary tumors, or cases with prior cancer history were excluded for the survival analyses to ensure consistency and avoid potential bias in survival outcomes. Statistical data are presented as mean ± SD. ANOVA (**B**), Student’s t-test (**C**); F test **(D** and **E)** and Gehan-Breslow-Wilcoxon test; (**F** and **G)** were applied for statistical analyses. ns, not statistically significant. (*p < 0.05, **p < 0.01).

We next assessed p190A protein levels in a tissue microarray composed of BC patient samples at different pathological stages in order to comprehend the association of p190A levels with the clinical progression and outcomes of BC using immunohistochemical (IHC) staining (Fig. 2A). Evaluation of the staining results revealed a significant decline in p190A expression from non-muscle-invasive bladder cancer (NMIBC) tissues (pTa + pT1) (*n* = 50) to muscle-invasive bladder cancer (MIBC) tissues (pT2 + pT3 + pT4) (*n* = 152) (p190A staining scores: 4.8 in NMIBC group vs. 3.8 in MIBC group, *p* < 0.01; Fig. 2B and C). These results imply that decreased p190A levels are associated with higher malignancy and invasiveness in BC. It is also noteworthy to mention that patients with primary BC expressing high levels of p190A are less prone to develop lymph node metastases (p190A staining scores: 3.8 in patients without lymph node metastases (N0) (*n* = 82) vs. 3.2 in patients with lymph node metastases (N+) (*n* = 39), *p* < 0.01; Fig. 2D) and demonstrate a reduced likelihood of relapse (p190A staining scores: 3.9 in patients without relapse (*n* = 54) vs. 3.5 in patients with relapse (*n* = 78), *p* < 0.05; Fig. 2E). Moreover, we found that MIBC patients with high p190A levels (*n* = 45) tend to have better overall survival (OS) and recurrence-free survival (RFS) outcomes compared to MIBC patients with low p190A levels (*n* = 46) (*p* < 0.05) (Fig. 2F and G). For this analysis, we defined the staining 4 as a cutoff to differentiate between high and low expression levels, as this was the average p190A score of all patient samples. Collectively, these findings suggest that p190A may function as a tumor suppressor protein and could serve as a potential prognostic biomarker in BC.

### p190A impairs migration and invasion capacities of BC cells

**Fig. 3:**
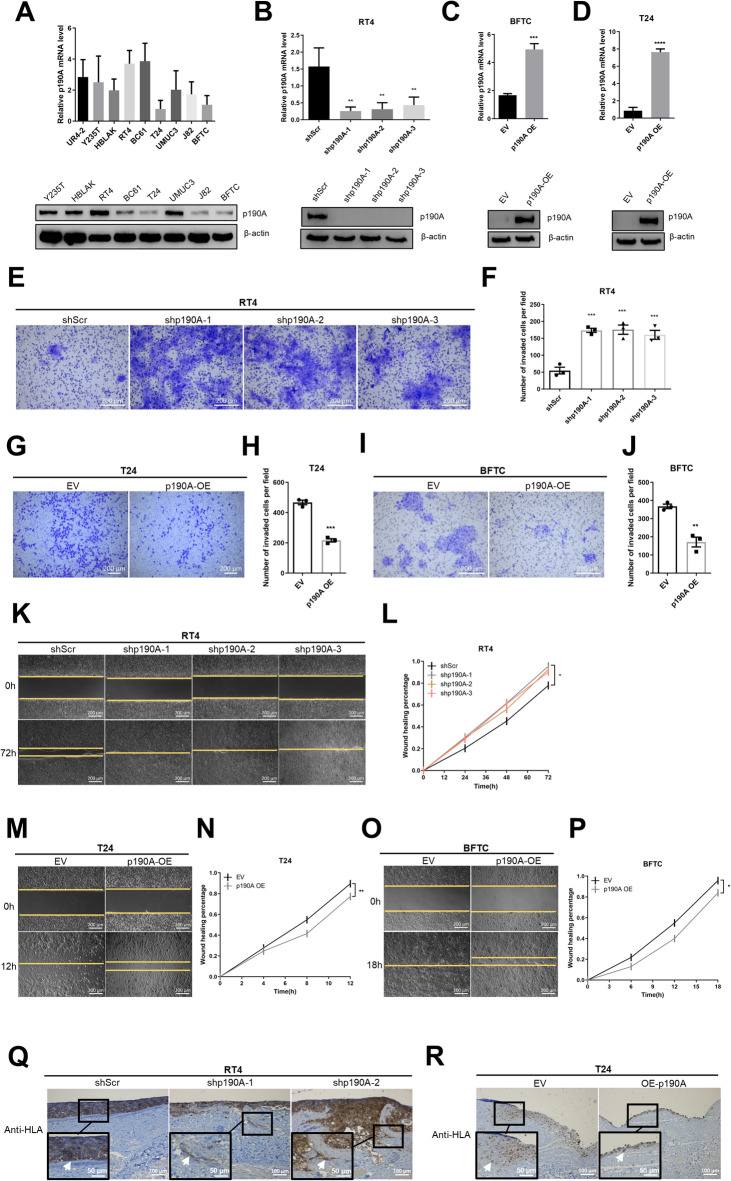
A p190A impairs invasion and migration capacities of BC cells both in in vitro and ex vivo experiments. **(A)** Relative mRNA and protein levels of p190A in normal ureter UR4-2, immortalized urothelial cell lines Y235T, HBLAK, low invasive bladder cancer cell lines RT4, BC61, and high invasive bladder cancer cell lines T24, UMUC3, J82, and BFTC. **(B**,** C**,** D)** Relative mRNA and protein levels of p190A in RT4-p190A-knockdown cells and BFTC/T24-p190A-overexpression cells. β-actin was used as a loading control. Representative images **(E**,** G**,** I)** and quantification **(F**,** H**,** J)** show that cell invasion is increased in RT4 cells with p190A knockdown and decreased in T24 and BFTC cells with p190A overexpression in Boyden chamber assay. Scale bar, 200 μm. Representative images **(K**,** M**,** O)** and quantification **(L**,** N**,** P)** show that cell migration is increased in RT4 cells with p190A knockdown and decreased in T24 and BFTC cells with p190A overexpression in would healing assay. Scale bar, 200 μm. Ex vivo porcine urinary bladder (PUB) model show that cell invasion is **(Q)** increased in RT4 cells with p190A knockdown and **(R)** decreased in T24 cells with p190A overexpression. The white arrow indicates cell invasion. The invasiveness of cells was detected by immunohistochemistry (IHC) staining with human leukocyte antigen (HLA) antibody. Scale bar, 100 μm and 50 μm. All experiments were performed at least in triplicates (*n* = 3). Statistical data are presented as mean ± SEM. ANOVA (for **B**,** F**,** L)** and Student’s t-test (for **C**,** D**,** H**,** J**,** N**,** P**) were applied for statistical analyses. (* *p* < 0.05, ** *p* < 0.01, *** *p* < 0.001, **** *p* < 0.0001).

Having demonstrated an association between p190A and BC initiation and progression, we employed functional experiments to investigate the role of p190A in BC cell lines. We initially assessed p190A mRNA in several immortalized, but non-cancerous cell strains derived from ureters (Y235T and HBLAK) and BC cell lines exhibiting varying invasive capacities, specifically low or no invasiveness (RT4 and BC61) and high invasiveness (T24, UMUC3, J82, and BFTC). Quantitative PCR and immunoblotting analyses revealed that p190A levels were significantly higher in the non-cancerous cells and in BC cell lines with low or no invasive characteristics, compared to BC cell lines with high invasive capacities. This suggests that p190A expression levels are inversely correlated with the invasive potential of the BC cell lines (Fig. 3A). In order to investigate the impact of p190A on the invasive capacity of BC cells, we knocked down its expression in the RT4 cell line, which exhibits non/low invasive and migratory abilities. Conversely, p190A was ectopically expressed in the T24 and BFTC cell lines, known for their high invasive and migratory capacities. The efficacy of the knockdown and ectopic expression was validated by RT-qPCR and WB analyses (Fig. 3B-D).

To evaluate the impact of p190A on tumor cell migration, we applied the wound healing method (also known as Scratch assay), while its influence on tumor cell migration and invasion was investigated by the Boyden chamber (using Matrigel-coated inserts on apical side of the membrane) and the ex vivo porcine bladder invasion assays, respectively. The representative pictures and the quantitation of Boyden chamber assay shows that p190A knockdown enhanced the migratory and invasive capacities of the RT4 cell lines (3.2-fold increase with RT4-shp190A-1 and shp190A-2 and 2.9-fold increase with RT-shp190A-3 compared to RT4-shScr cells, all *p* < 0.001), while p190A ectopic expression impairs the migratory and invasive capacities of T24 and BFTC cell lines (2.2-fold decrease with T24-p190A compared to T24-EV, *p* < 0.001; 2.1-fold decrease with BFTC-p190A compared to BFTC-EV, *p* < 0.01; Fig. 3E-J). Given that Boyden chamber assay measures both migration and invasion and capacities of cells, we also employed a wound-healing assay to specifically assess cell migration. Consistent with the results of the Boyden chamber assays, stable knockdown of p190A promoted migration rate of RT4 cells (percentage of closed area after 72 h: 81% in RT4-shScr vs. 95% in RT4-shp190A-1, *p* < 0.05, 93% in shp190A-2, and 90% in shp190A-3 cells; Fig. 3K and L). In contrast, the overexpression of p190A inhibited migration of T24 cells (percentage of closed area after 12 h: 90% in T24-EV vs. 77% in T24-p190A, *p* < 0.01; Fig. 3M and N) and BFTC cells (Percentage of closed area after 18 h: 96% in BFTC-EV vs. 84% in BFTC-p190A, *p* < 0.05; Fig. 3O and P). While we cannot fully exclude a proliferative component in this assay, the wound closure experiments support the impact of p190A on cell motility. Importantly, MTTs assays (Suppl. Figure 1) showed that p190A knockdown reduced cell proliferation, while migration ability of the RT4-shp190A cells was increased. Conversely, ectopic p190A in BFTC cells promoted cell proliferation while inhibiting cell migration. These results rather support a proliferation-independent impact of p190A on cell migration. We further investigated the influence of p190A on migratory and invasive capacities of BC cell lines using an ex vivo porcine bladder approach ^30,31^. As shown in representative images, RT4 cells with low p190A expression invaded into the stroma and muscle layers of porcine bladder tissue, whereas the control RT4-shScr cells formed a smooth epithelium-like layer on its de-epithelized surface and showed no invasiveness (Fig. 3Q), similar to previous observations^33^. p190A ectopic expression, on the other hand, decreased the capacity of T24 cells to infiltrate the stroma and muscle layers of the porcine bladder tissue (Fig. 3R).

### p190A reduces invadopodia formation in BC cells

**Fig. 4:**
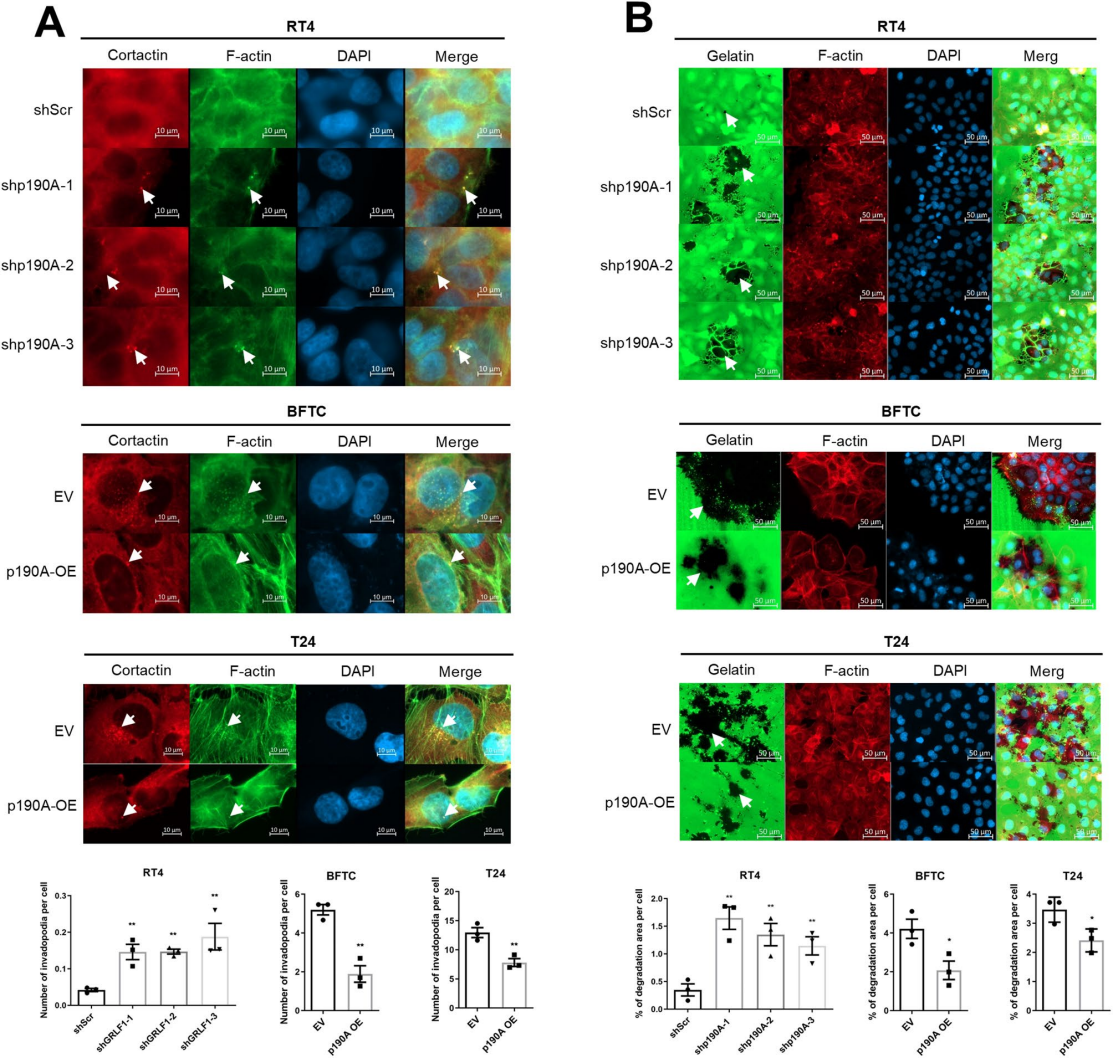
A p190A reduces invadopodia formation and gelatin degradation in BC cells. **(A)** Representative immunofluorescence (IF) images and quantification of invadopodia formation in bladder cancer cells. Invadopodia were identified by co-localization of F-actin and cortactin (white arrows). Scale bar, 10 μm. To quantify invadopodia formation, F-actin and cortactin double-positive puncta were counted from 8–10 random microscopic fields per condition. The number of invadopodia per cell was calculated by dividing the total number of invadopodia by the total number of cells. **(B)** Representative images and quantification of gelatin degradation in bladder cancer cells. Assessment of the functional consequences of altered invadopodia formation was investigated by fluorescent gelatin degradation assays. Areas of matrix degradation appear as dark regions beneath or within cell clusters (white arrows). Scale bar, 50 μm. Degradation was quantified from 8–10 random fields per condition. Gelatin degradation capacity of the cells was quantified by measuring the degradation area per cell and the degradation area per cell was calculated. N-numbers indicate total cell number from three independent experiments (RT4-shScr: *n* = 949; RT4-shp190A-1: *n* = 1185; RT4-shp190A-2: *n* = 1722; RT4-shp190A-3: *n* = 839; BFTC-EV: *n* = 702; BFTC-p190A: *n* = 1218; T24-EV: *n* = 1059 and T24- p190A: *n* = 934). **A**,** B** All experiments were performed at least in triplicates (*n* = 3). Quantification was based on the results of three independent experiments, and results are presented as mean ± SEM. ANOVA (RT4) and Student’s t-test (BFTC, T24) were used for the statistical analyses. (* *p* < 0.05, ** *p* < 0.01).

Invadopodia play a crucial role in cell adhesion and the remodeling of the extracellular matrix (ECM) during the process of cell invasion. Cortactin, an F-actin-binding protein that is abundant in invadopodia and F-actin/cortactin association serves as an early marker for invadopodia formation. Consequently, we examined whether p190A affects the formation of invadopodia through F-actin/cortactin colocalization analysis (Fig. 4A, Suppl. Figure 5). The results indicated that the depletion of p190A in RT4 cells increased invadopodia formation compared to cells transduced with a non-targeting shRNA. The combined data from three independent experiments shows that RT4-shScr formed 0.041 invadopodia per cell (31 invadopodia/754 cells), while knockdown of p190A increased invadopodia per cell to 0.141 in RT4-shp190A-1 (103 invadopodia/730 cells, thus 3.4-fold increase compared to RT4-shScr), to 0.147 in RT4-shp190A-2 (118 invadopodia/803 cells, thus 3.6-fold increase compared to RT4-shScr), and to 0.1934 in RT4-shp190A-3 (106 invadopodia/548 cells, thus 4.7-fold increase compared to RT4-shScr). Conversely, ectopic expression of p190A in BFTC and T24 cells reduced their ability to form invadopodia. While in BFTC-EV cells, 4.3 invadopodia per cell were detected (2209 invadopodia/509 cells), only 1.5 invadopodia per cell (899 invadopodia/579 cells) could be detected in BFTC-p190A cells, indicating a 2.8-fold reduction of invadopodia formation in response to ectopic p190A expression. Similarly, in T24-EV cells, 13.9 invadopodia per cell were detected (3495 invadopodia/251 cells), while in T24-p190A cells, 7.2 invadopodia per cell were detected (2106 invadopodia / 293 cells), corresponding to a 1.9-fold decrease of invadopodia formation in response to ectopic p190A expression. In summary, while p190A knockdown in RT4 cells significantly increased invadopodia formation compared to control cells (RT4-shScr), ectopic p190A expression in BFTC and T24 cells led to a marked reduction of invadopodia formation. We also performed gelatin degradation assays to investigate the influence of p190A on invadopodia formation at the subcellular level and to correlate invadopodia formation to cell migration. A significant increase of gelatin degradation, which was evident as dark regions both at the periphery and, more strikingly, within the interior of cell clusters, was observed with RT4-shp190A-1, shp190A-2, and shp190A-3 cells compared to RT4-shScr cells (5.4-fold, 4.5-fold, and 3.8-fold, respectively; *p* < 0.01 for all comparisons). In contrast, p190A ectopic expression resulted in reduced gelatin degradation activity of BFTC cells (2.3-fold) and of T24 cells (1.4-fold), respectively, compared to their empty vector (all *p* < 0.05; Fig. 4B). In order to clarify whether the above described gelatin degradation alterations in 2D cell culture are matrix metallo-proteinase (MMP)-dependent, expression of several MMPs was determined by RT-qPCR and gelatin and collagen zymography experiments were conducted (Suppl. Figure 6). While there were no significant changes in MMP expression as well as gelatin and collagen degradation upon p190A downregulation in RT4 cells, we observed increased mRNA levels of some MMPS (e.g. MMP1 and MMP2) and changes in MMP enzymatic activity in BFTC cells with ectopic p190A. Interestingly, the expression of MMP1 and MMP2 was increased in response to p190A expression. In summary, the expression and activity of MMPs do not correlate to the observed alterations in 2D cell culture gelatin degradation experiments.

### p190A regulates actin dynamics through Cofilin and cortactin

**Fig. 5:**
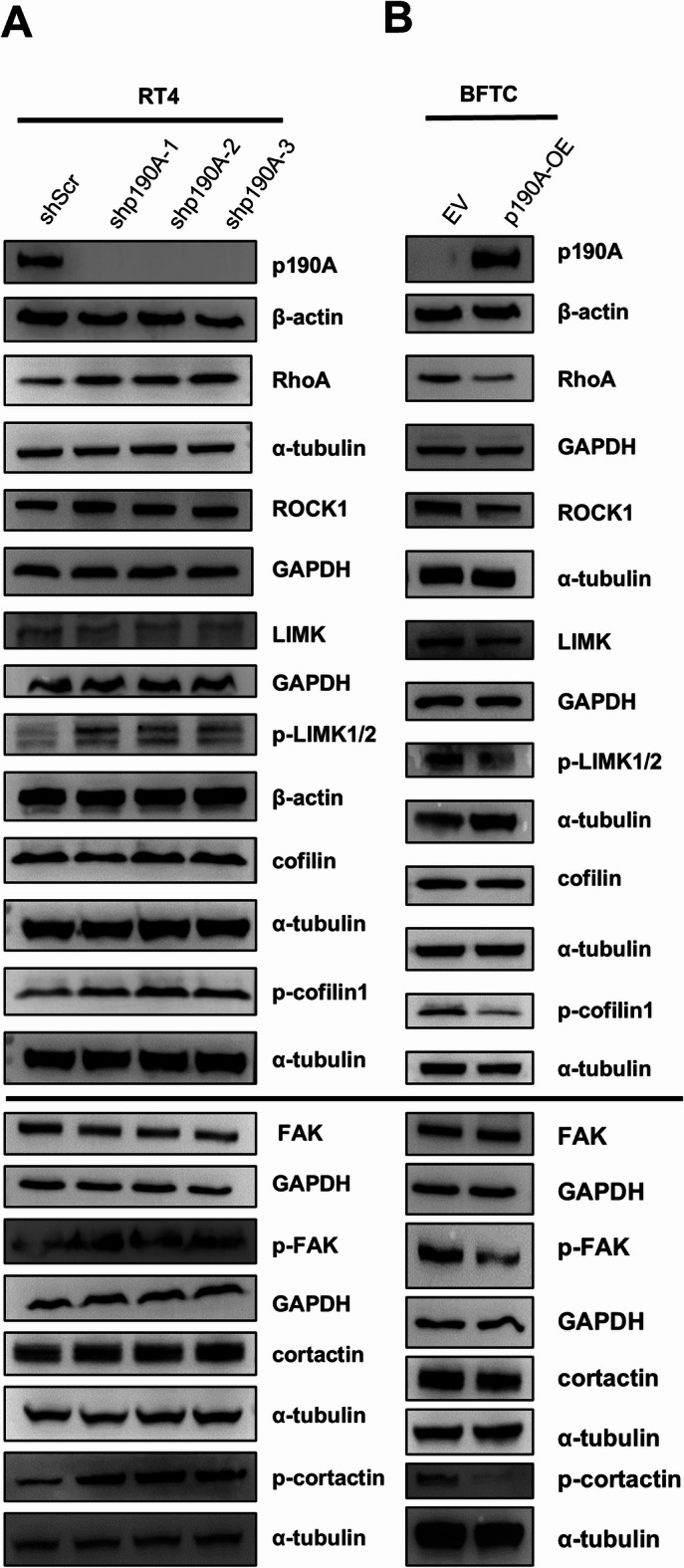
A p190A regulates LIMK/Cofilin and FAK/cortactin pathways in BC cells. (A) Western blots show that the expression of RhoA, ROCK1, phospho-LIMK1/2 (p-LIMK1/2), and phospho-cofilin1 (p-cofilin1) are upregulated upon p190A knockdown in RT4 cells using three different shRNAs (shp190A-1, shp190A-2, shp190A-3), while total cofilin and LIMK levels remained unchanged. Similarly, phospho-cortactin (p-cortactin) and phospho-FAK (p-FAK) levels are increased in cells with p190A knockdown but the total cortactin and FAK levels remain unchanged. **(B)** Western blots show that the expression of RhoA, ROCK1, phospho-LIMK1/2 (p-LIMK1/2), and phospho-cofilin1 (p-cofilin1) are downregulated upon ectopic p190A expression in BFTC cells (p190A-OE), while p190A overexpression did not influence total cofilin and LIMK levels. Similarly, phospho-cortactin (p-cortactin) and phospho-FAK (p-FAK) levels are downregulated upon p190A overexpression (BFTC-OE) but the total cortactin and FAK levels remain unchanged. α-tubulin, β-actin and GAPDH were used as loading controls. Please note that for all Western blots (WB), full-length gels are provided in the supplemental data file (Suppl. Figure 2). Quantification of all WBs shown in Fig. 5, including full gels of the WBs are provided as supplemental data (Suppl. Figure 3). The Western blots in Figs. 1, 3 and 5 were repeated at least for three times (Suppl. Figure 7). Please note that due to the experimental requirements, i.e. multiple use of the membranes with different antibodies, different loading controls were used in some cases in Figs. 1, 3 and 5.

Aneuploid cells often exhibit cytoskeletal abnormalities, leading to enhanced or impaired actin polymerization, altered adhesion dynamics, and increased contractility through dysregulated Rho GTPase signaling. p190A is an important Rho GTPase-activating protein that regulates actin dynamics via the RhoA/ROCK1/LIMK/Cofilin and the FAK/cortactin pathways. Moreover, LIM kinases (LIMK1/2) have been implicated in regulating the mitotic spindle organization, chromosome segregation, and cytokinesis during the cell division while cortactin impacts cytokinesis through actin organization. Therefore, we investigated whether p190A regulates these pathways in the BC cell lines. The knockdown of p190A in RT4 cells showed a tendency towards an increase in RhoA and ROCK1 levels and consequently to the activation of LIM kinases (p-LIMK1/2) and increased phospho-cofilin (p-cofilin) levels (Fig. 5A). Similarly, we observed a tendency towards an increased phosphorylation of the focal adhesion kinase (p-FAK) and of its target protein cortactin (p-cortactin) in RT4-shp190A cells, while the total protein levels remained unchanged (Fig. 5A). More disctinctly, ectopic expression of p190A resulted in reduced RhoA and ROCK1 levels and consequently impaired phosphorylation of LIMK1/2 and cofilin as well as in reduced phosphorylation of FAK and cortactin (Fig. 5B).

## Discussion

In this study, we revealed that p190A has a tumor suppressor function in bladder tissue. We find that p190A downregulation is associated with increased genome instability in otherwise karyotypically normal urothelium cells, as well as enhanced cell motility and invasive behavior of BC cell lines. Evaluation of p190A protein levels in 202 BC patient samples from various clinical stages revealed that, regardless of tumor stage, lower p190A expression is associated with increased malignancy and tumor invasiveness. Particularly, lower p190A levels were associated with disease recurrence, lymph node metastases, and worse survival rates in patients with highly aggressive BC. Our results suggest that p190A regulates the function of the actin-binding proteins cofilin and cortactin in the bladder epithelium and plays a role in maintaining cell ploidy while its loss promotes tumor initiation and progression.

Given the positive correlation between aneuploidy and the invasive and metastatic potential of BC ^12^, it is important to note that our results indicate that both molecular processes are influenced by p190A in bladder tissue. We show for the first time that p190A simultaneously regulates phosphorylation of cofilin and cortactin, two major actin binding proteins. Importantly, actin dynamics have been shown to influence both cell division and motility, in particular through the interaction between microtubules and the actin cytoskeleton. Cofilin, an intracellular actin-modulating protein, promotes F-actin turnover by severing and depolymerizing actin filaments^35,36^. Through ROCK, RhoA inhibits cofilin activity by promoting the phosphorylation of LIMK, ultimately leading to the cleavage of F-actin^37,38^. The Rho/ROCK/LIMK/cofilin signaling cascade is crucial for various carcinogenic processes, including proliferation, survival, migration, and invasion of tumor cells, primarily due to its role in actin remodeling^39,40^. On the other side, cortactin, another F-actin-binding protein, plays a crucial role in regulating actin polymerization during the formation of invadopodia^41^, prominent structures involved in cell migration and metastasis. Invasive cells are characterized by invadopodia, which facilitates focal adhesions and the dynamic of cell motility^42^. In 1997, Nakahara et al. first identified the connection between p190A and downstream signaling pathways -involving Rho family GTPases- and the actin cytoskeleton in invadopodia^43^. Consistent with prior findings, we observed an increase in invadopodia formation and ECM degradation in cells lacking p190A. On the other hand, focal adhesions (FA) are F-actin binding attachments in cells and provide the dynamic center for the F-actin architecture^44^. The main regulator of focal adhesions is FAK, which can undergo autophosphorylation upon its recruitment to FA sites, following the binding of transmembrane integrin receptors to the ECM^45,46^. FAK and its main target protein cortactin are important for cell migration and are linked to cancer^47^. As we observe that p190RhoGAP silencing increases RhoA activity and alters phosphorylation of the acting regulatory proteins p-cortactin and p-cofilin (Fig. 5), it is conceivable that the influence of 190 A on actin contractility is primarily responsible for the impact of p190A on cell migration. This interpretation is further supported by MMP expression and zymography analyses (Supplementary Fig. 6), which indicate that the apparent gelatin degradation observed in the FITC-gelatin assays does not consistently correlate with the expression or activity of the tested MMPs. While the FITC-gelatin assay is a powerful tool for visualizing and quantifying the enzymatic degradation of the extracellular matrix in order to correlate MMP activity to cell migration, the migration of cells across the gelatin surface can sometimes lead to observations that mimic true degradation. This “apparent” degradation is not due to enzymatic activity but rather results from the physical forces exerted by the cells on the gelatin matrix, which supports the interpretation that p190A influences protease-independent cell motility^48,49^.

In conclusion, our findings identify p190A as a tumor suppressor in BC. The absence of p190A results in aneuploidy and genomic instability in urothelial cells, as well as enhanced cell migration and invasion capabilities in BC. In this line, expression of a constitutively active mutant of the Wiskott-Aldrich Syndrome protein (WASP) results in excessively high levels of F-actin, which subsequently induces aneuploidy in cells^50,51^. A study from our laboratory demonstrated that ORP3 downregulation promotes BC migration and invasion, as well as aneuploidy induction in normal uretelium cells, through interactions with γ-tubulin and F-actin^31^. In line with this, we recently reported that loss of the connexin protein GJB3 mechanistically interacts with α-tubulin and F-Actin. We showed that GJB3 deficiency influences microtubule and actin dynamics, leading to aneuploidy and the formation of invadopodia^30^. Similarly, p190A knockdown resulted in aneuploidy and impaired mitosis in normal urothelial cells. We propose that the mechanism by which p190A regulates actin dynamics in the context of bladder cancer migration and invasion may also elucidate the role of p190A dysfunction in maintaining genome stability. This association adds evidence to the concept that increased cancer cell invasiveness during tumor progression is associated with increased aneuploidy.

As a future outlook, it should be emphasized that the present study could not cover of the different aspects related to the different functions and regulatory pathways that are associated with p190A. As such, increasing number of studies link the tumor suppressor function of p190A to Hippo-YAP signaling pathway ^52–55^. Although not analyzed in this study, it is conceivable to assume that p190A also regulates tumor initiation and/or progression in bladder cancer through this central pathway. This aspect remains to be studied in future investigations.

## Supplementary Information

Below is the link to the electronic supplementary material.

Supplementary Material 1

Supplementary Material 2

Supplementary Material 3

Supplementary Material 4

Supplementary Material 5

Supplementary Material 6

Supplementary Material 7

Supplementary Material 8

## Data Availability

Data generated and analyzed in this study are included in the manuscript and supplementary files. Additional information is available from the corresponding author on reasonable request.
