# Supplementary material for: Loss of p190A RhoGAP induces aneuploidy and enhances bladder cancer cell migration and invasion by modulating actin dynamics
Source: Sci Rep. 2025 Nov 18;15:40399. doi: 10.1038/s41598-025-23687-4 (PMC12627482; doi:10.1038/s41598-025-23687-4)
Supplement: Supplementary file 1 — Supplementary Material 1 [file 41598_2025_23687_MOESM1_ESM.pdf]

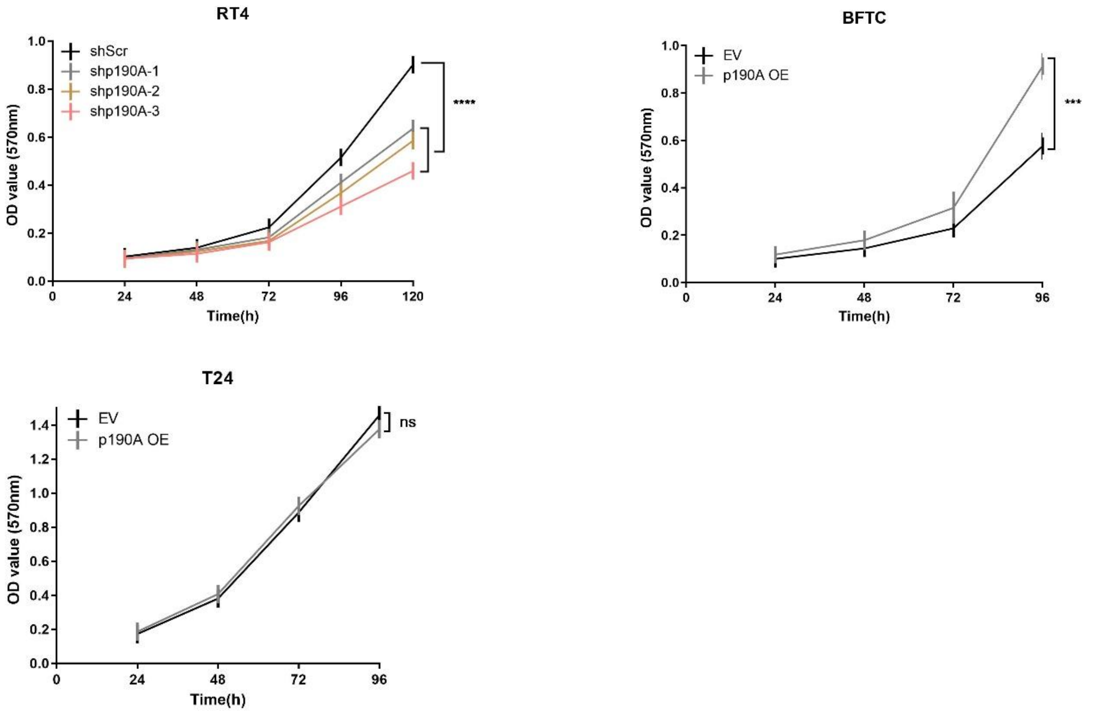

Figure S1. p190A influences viability of bladder cancer cells in MTT assay. Cell viability is decreased in RT4 cells upon p190A knockdown compared to scramble control (shScr), while it is increased in BFTC and T24 cells with p190A overexpression (OE) compared to empty control (EV). Cell proliferation in T24 EV and p190A OE cells shows no significant difference. Data are presented as mean ± SD (n=3, ns: $P > 0.05$, *** $p < 0.001$, **** $p < 0.0001$, Two-Way ANOVA).

MTT assay

Cell viability was quantified using a MTT assay. Cells were trypsinized and transferred into 15 ml sterile tubes. Cell numbers were counted three times and cells were diluted to 100,000 cells in 10 ml of culture medium. Cell suspension was mixed thoroughly and 100 µl cells were seeded per well in a 96-well plate with 6 replicates. The cells were repeatedly seeded in 5 plates for the different time points, and cell viability was detected at 24, 48, 72, 96, and 120-hour time points post seeding. To determine cell viability, 20 µl of 5 mg/ml MTT solution was added to each well. One set of wells was included with MTT but without cells (control). Subsequently, plates were placed into an incubator for 4 hours at 37°C and MTT assay was performed according to standard protocols and absorption was measured at 590 nm wavelength in a Spectrophotometer.
