# Supplementary material for: Loss of p190A RhoGAP induces aneuploidy and enhances bladder cancer cell migration and invasion by modulating actin dynamics
Source: Sci Rep. 2025 Nov 18;15:40399. doi: 10.1038/s41598-025-23687-4 (PMC12627482; doi:10.1038/s41598-025-23687-4)

Figure 1C

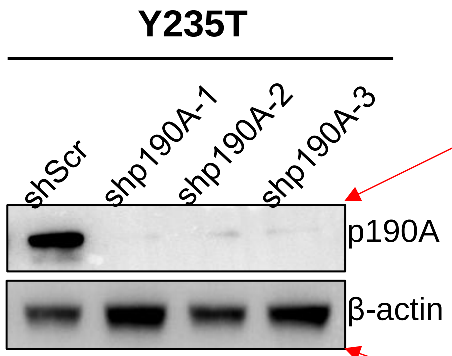

Y235T
shScr
shp190A-1
shp190A-2
shp190A-3
p190A
β-actin

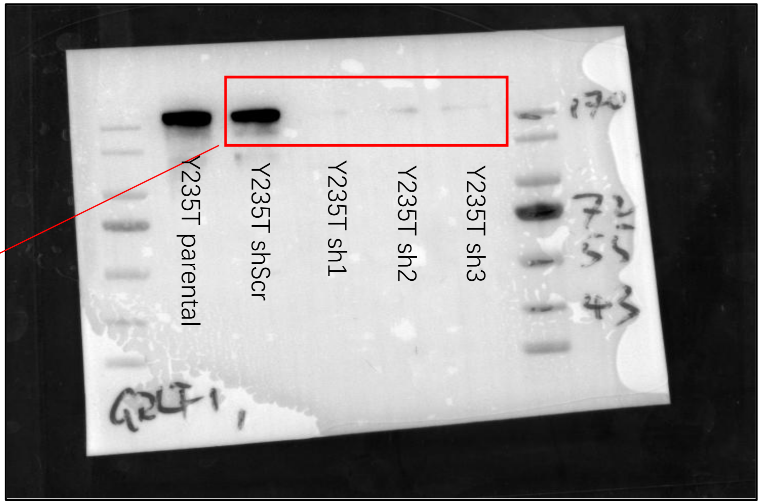

Y235T parental
Y235T shScr
Y235T sh1
Y235T sh2
Y235T sh3

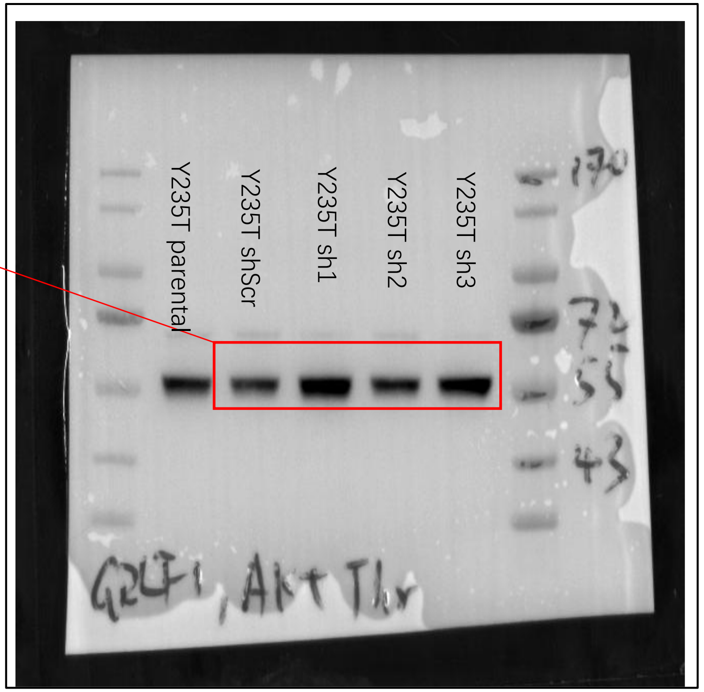

Y235T parental
Y235T shScr
Y235T sh1
Y235T sh2
Y235T sh3

Figure 3A

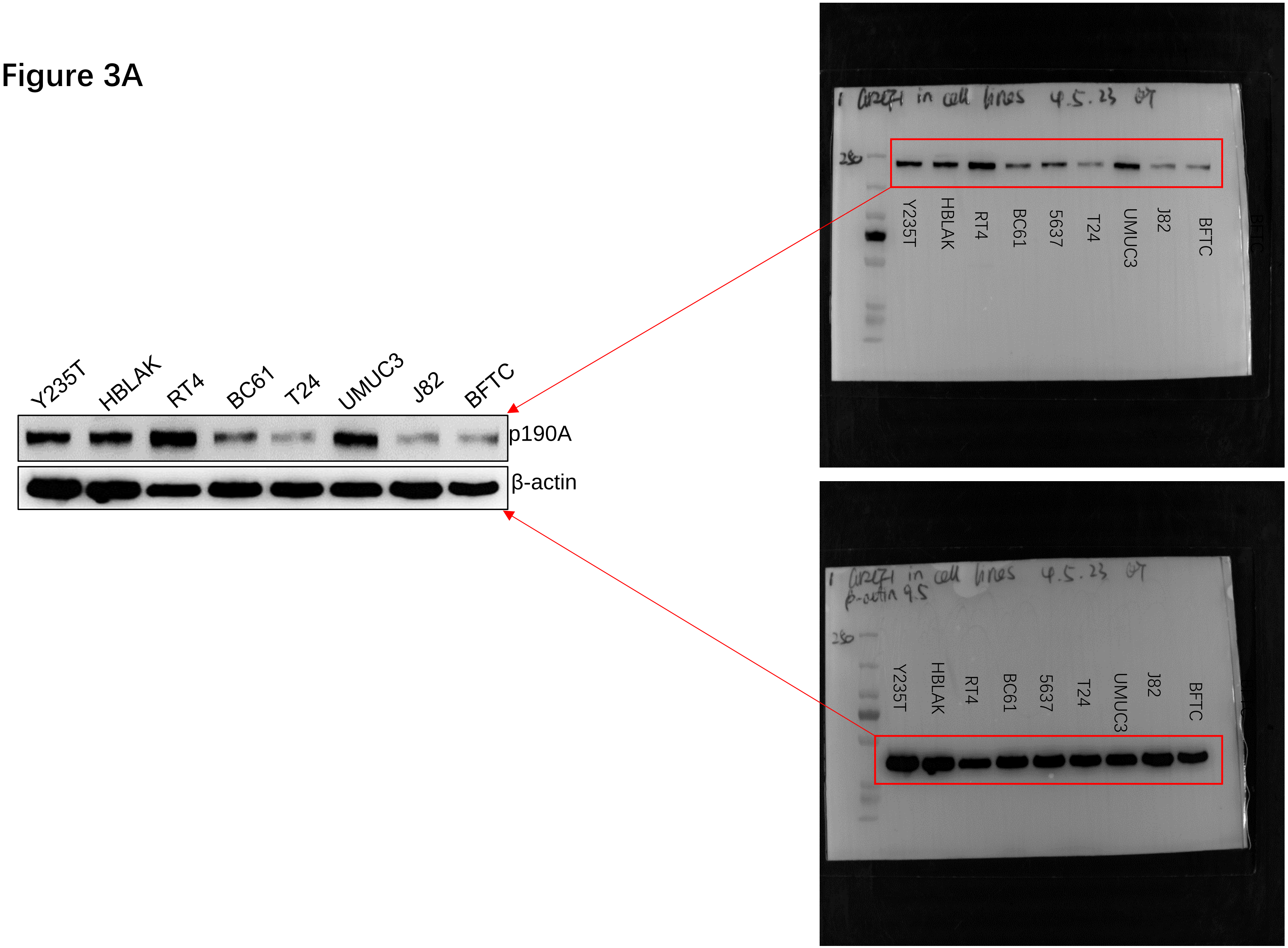
Y235T
HBLAK
RT4
BC61
T24
UMUC3
J82
BFTC
p190A
β-actin
Y235T
HBLAK
RT4
BC61
5637
T24
UMUC3
J82
BFTC

Figure 3B

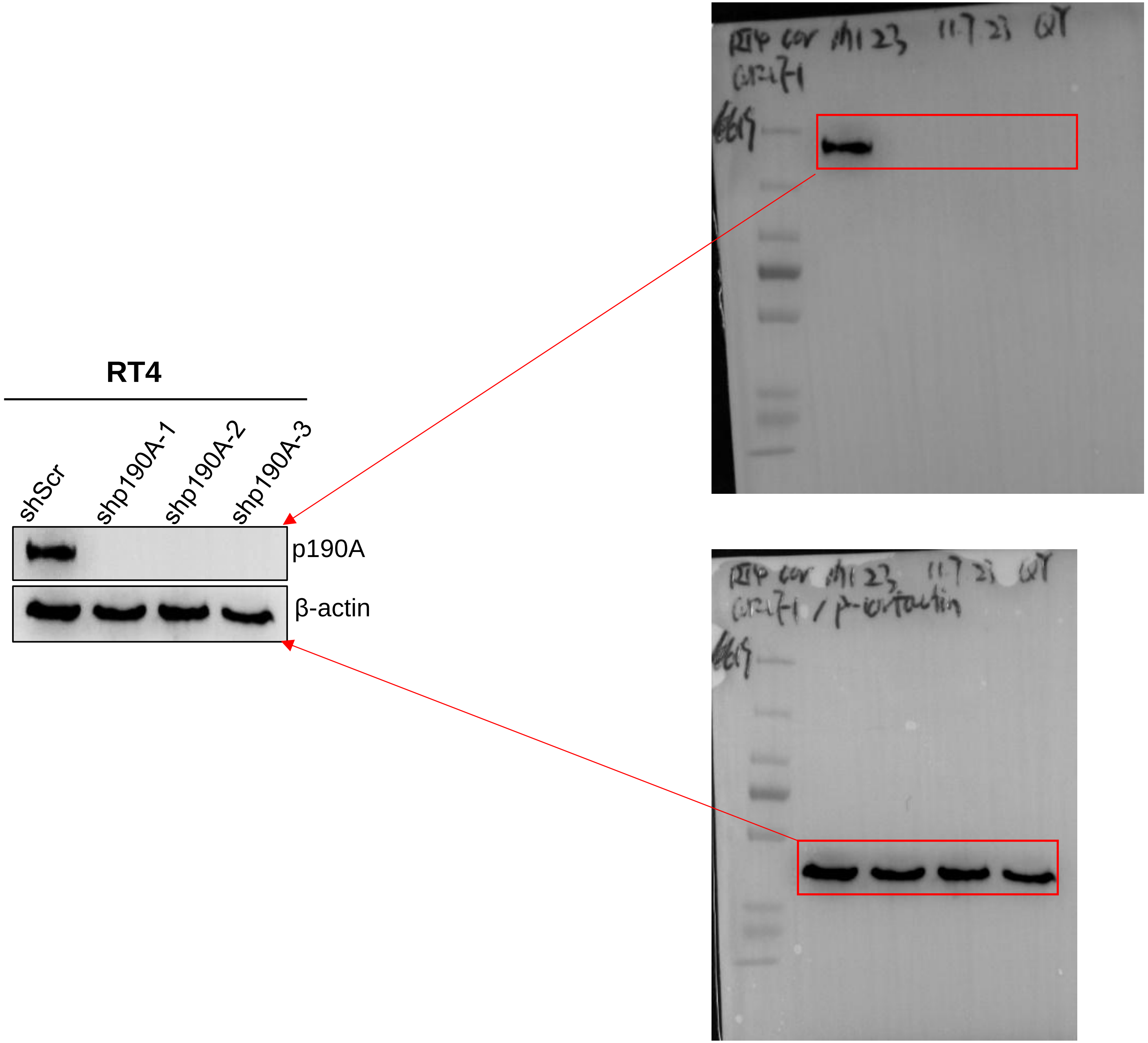

Figure 3C

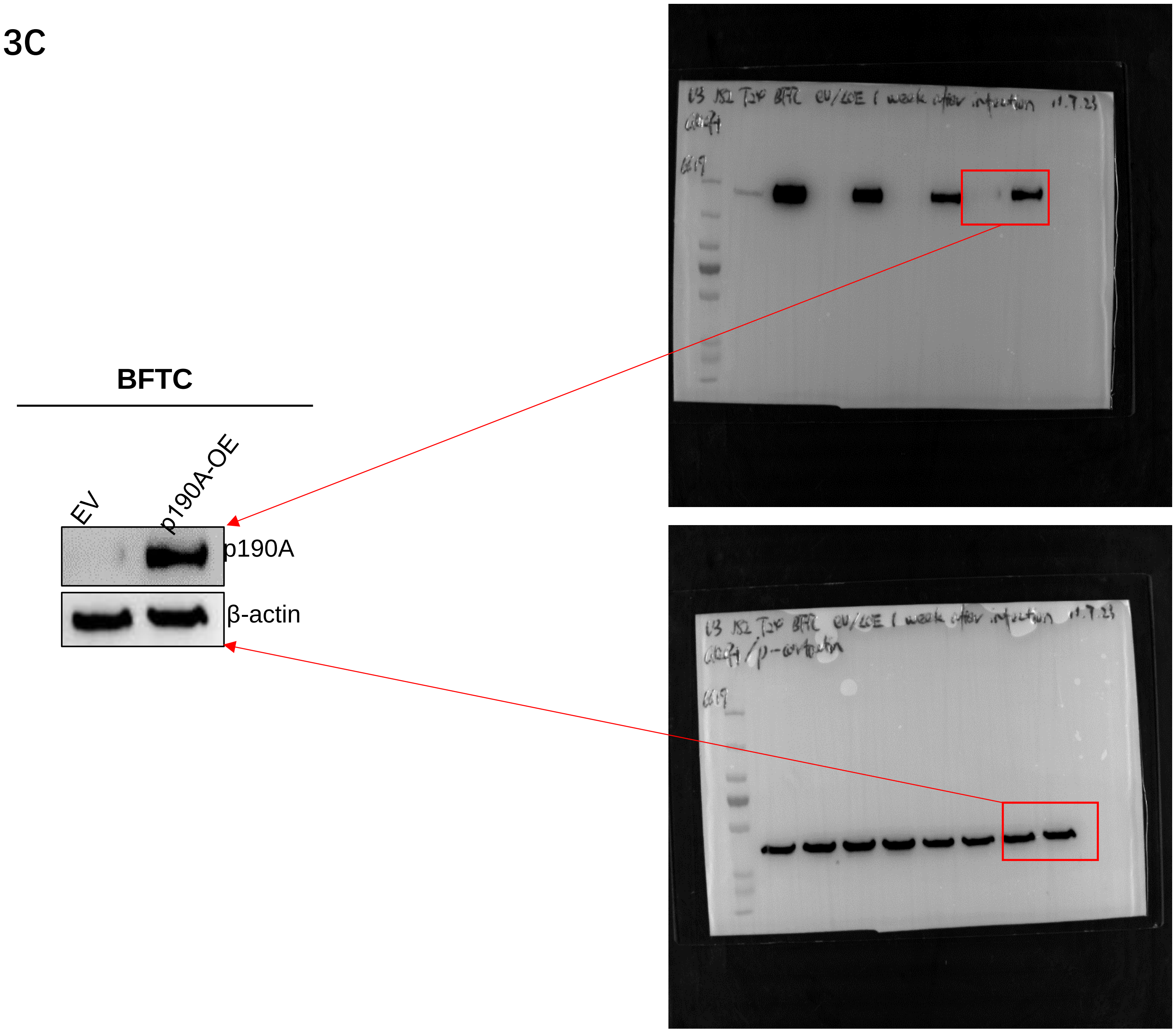

Figure 3D

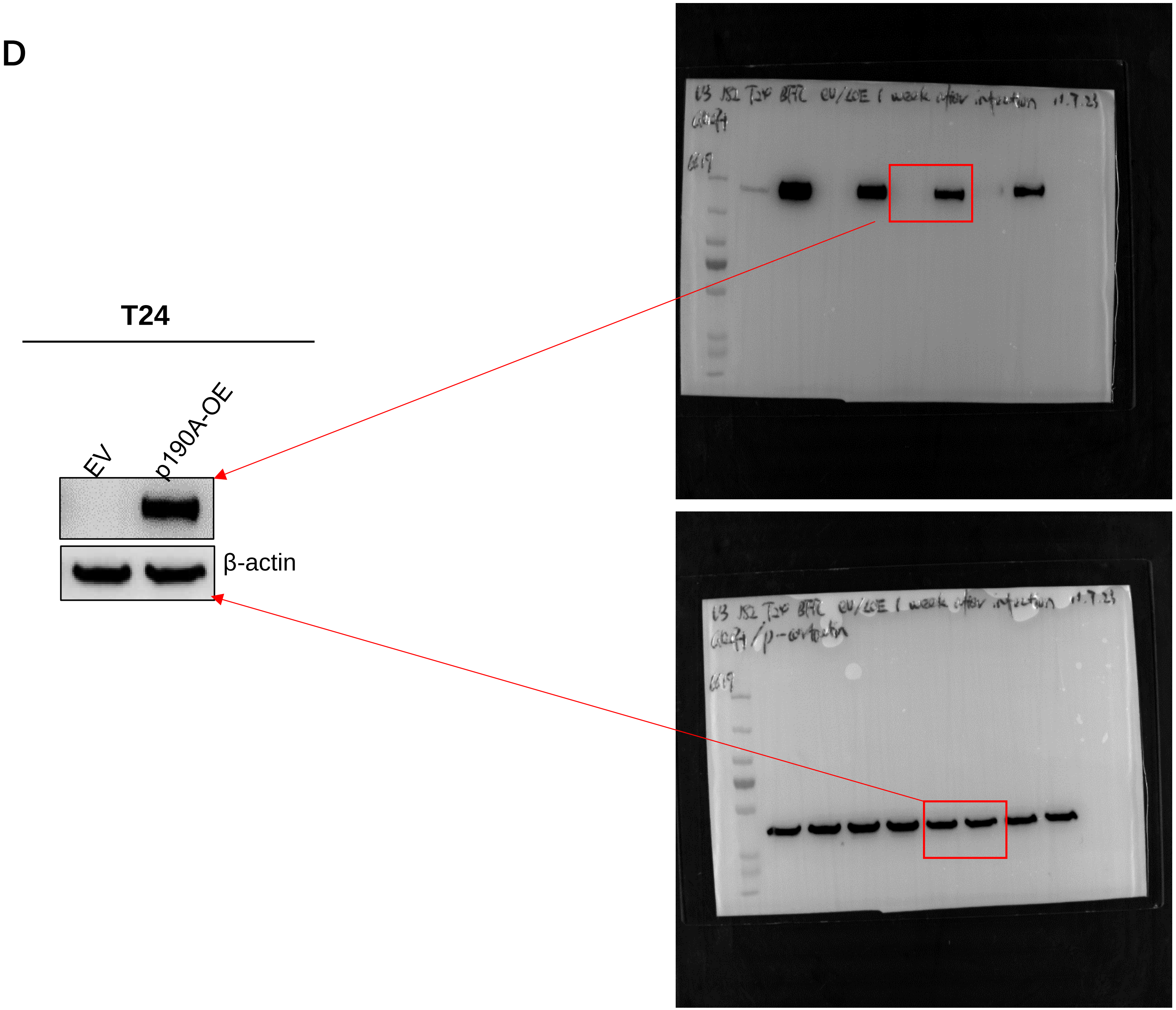

Figure 5A

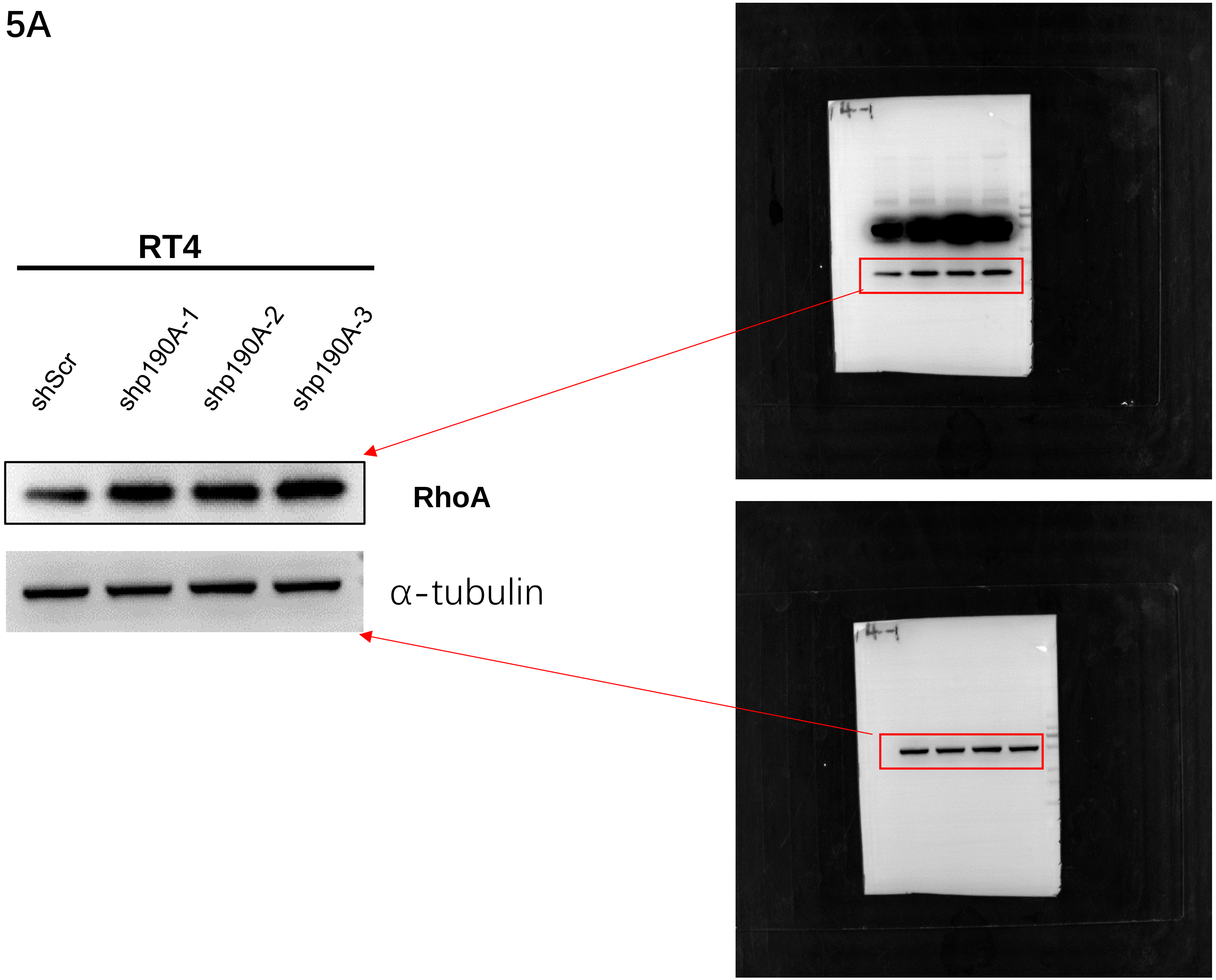

Figure 5A

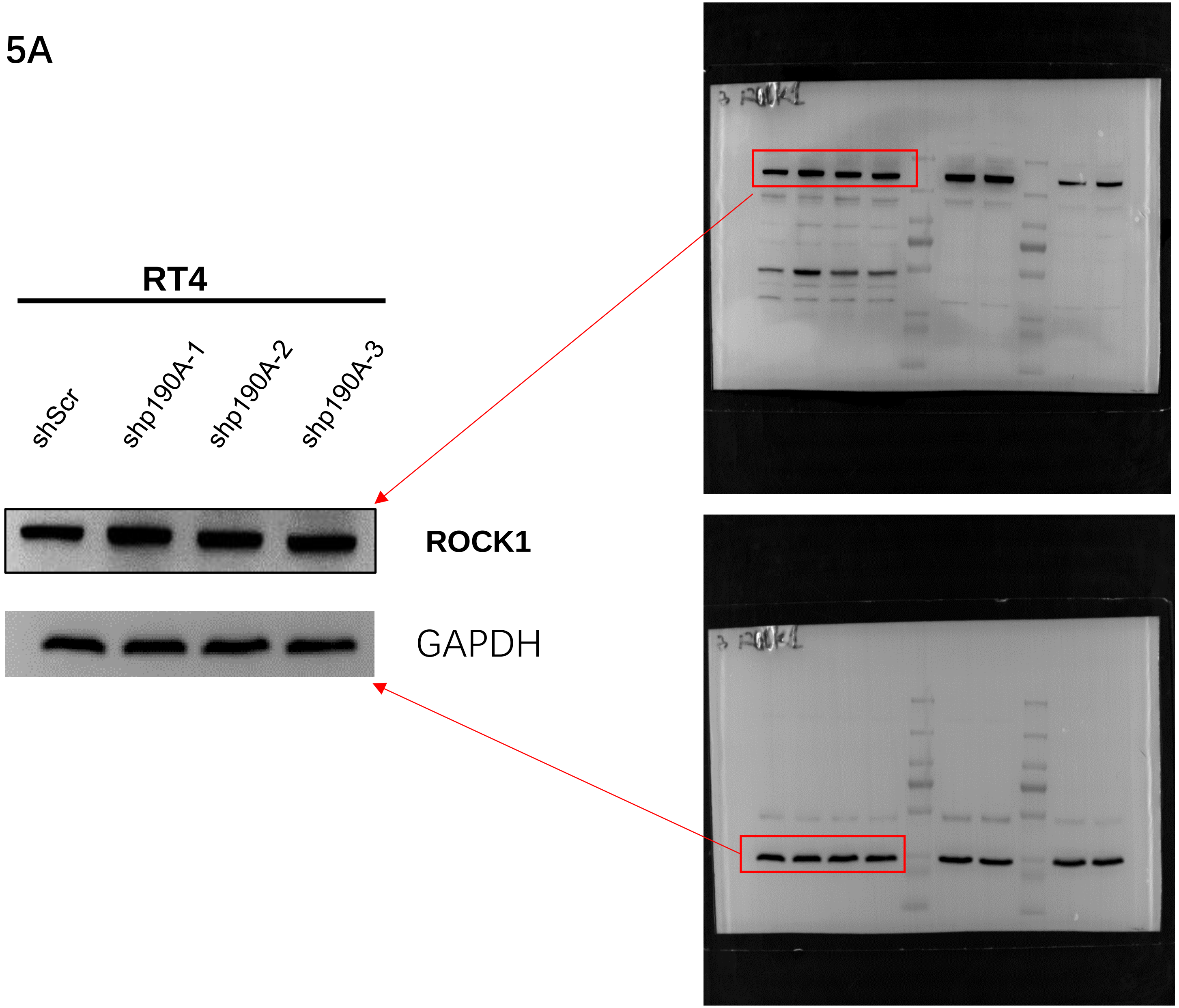

Figure 5A

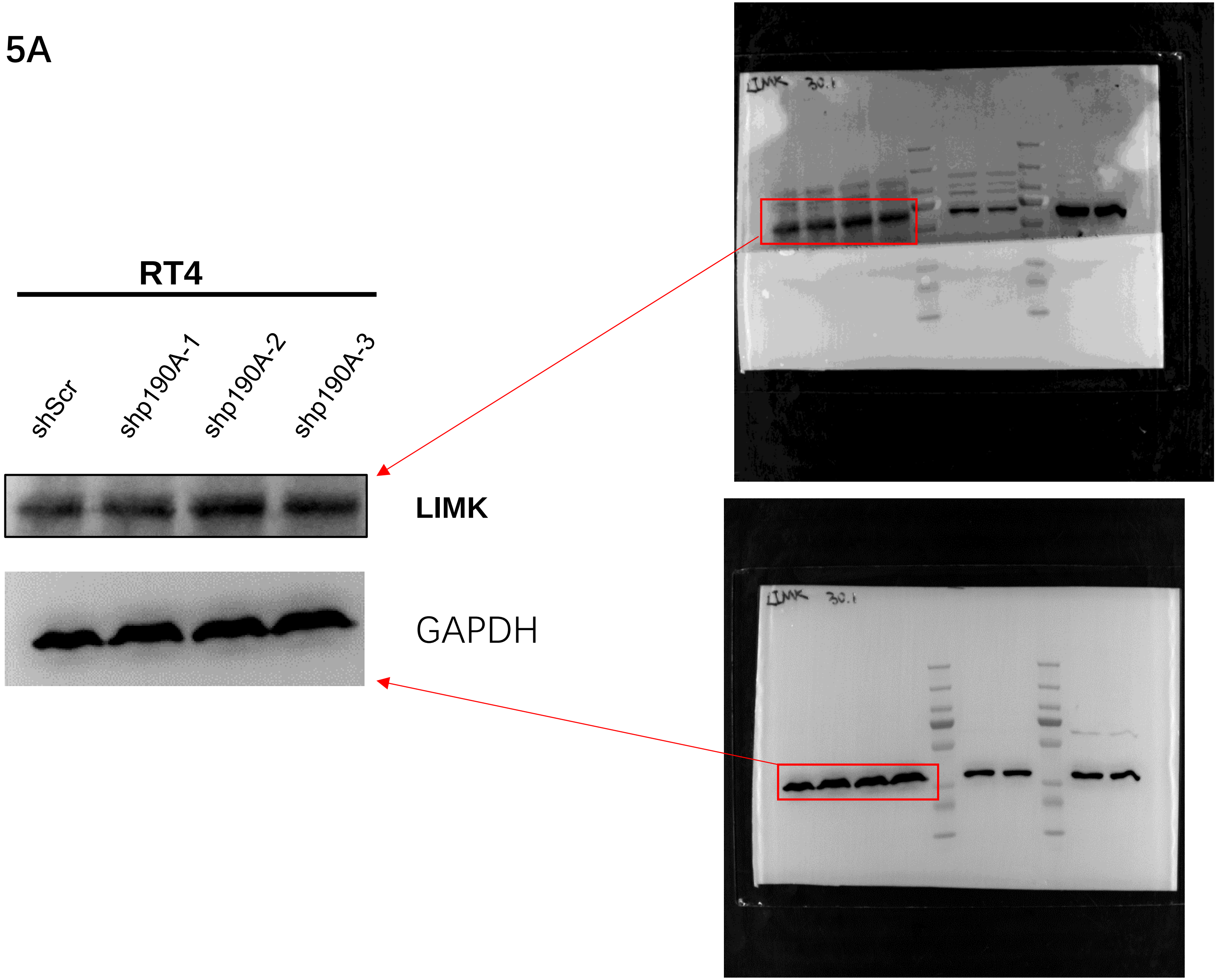

Figure 5A

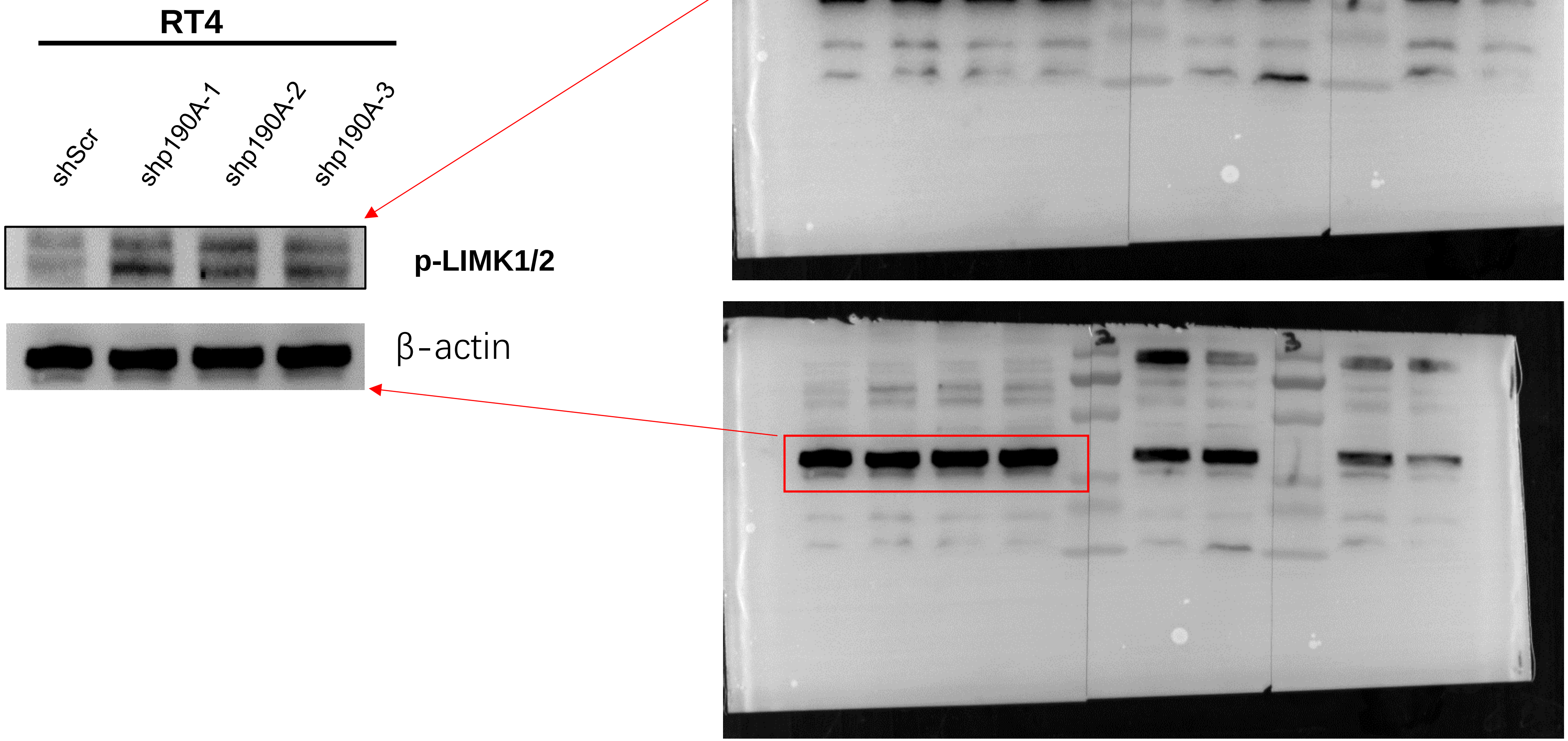

Figure 5A

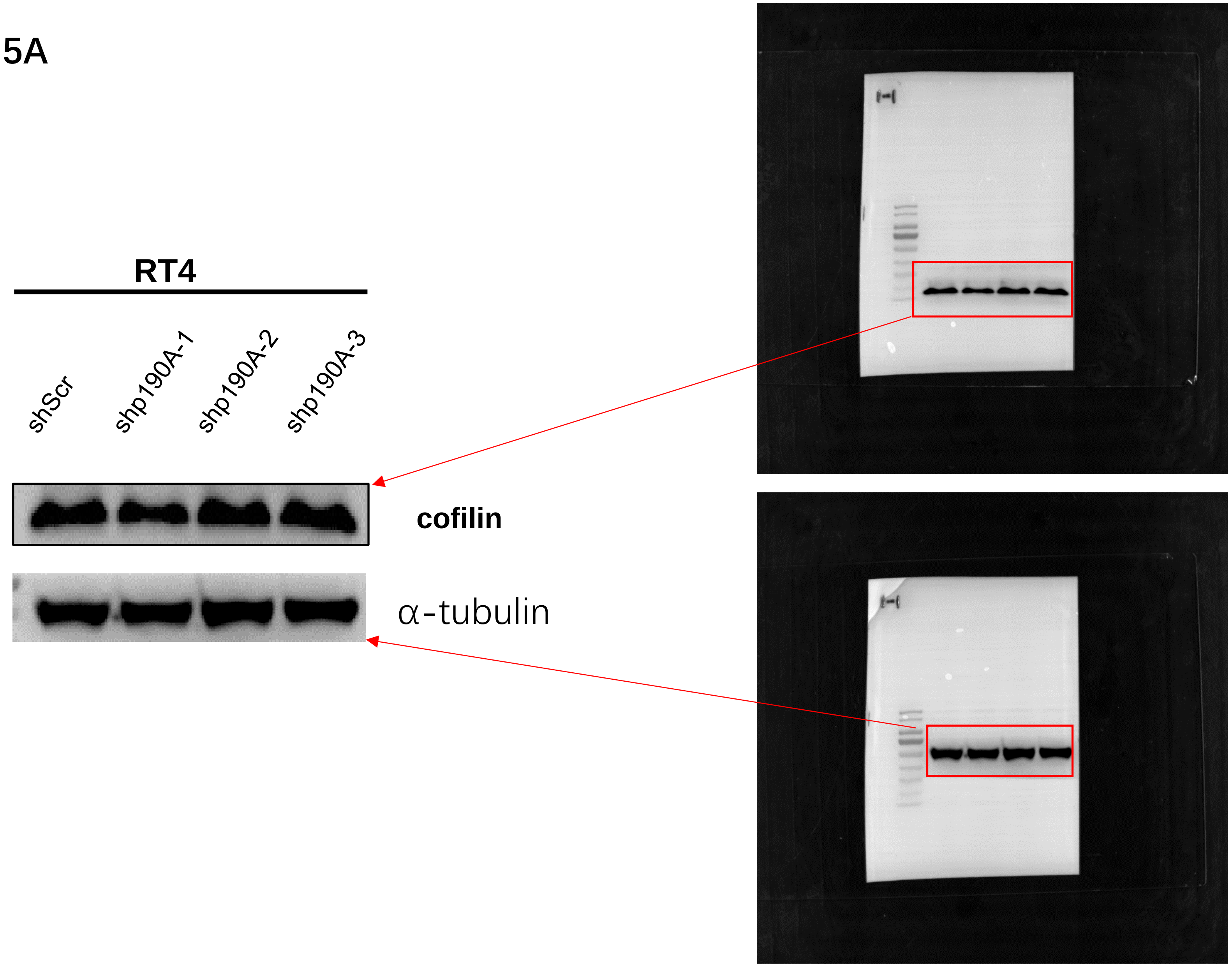

Figure 5A

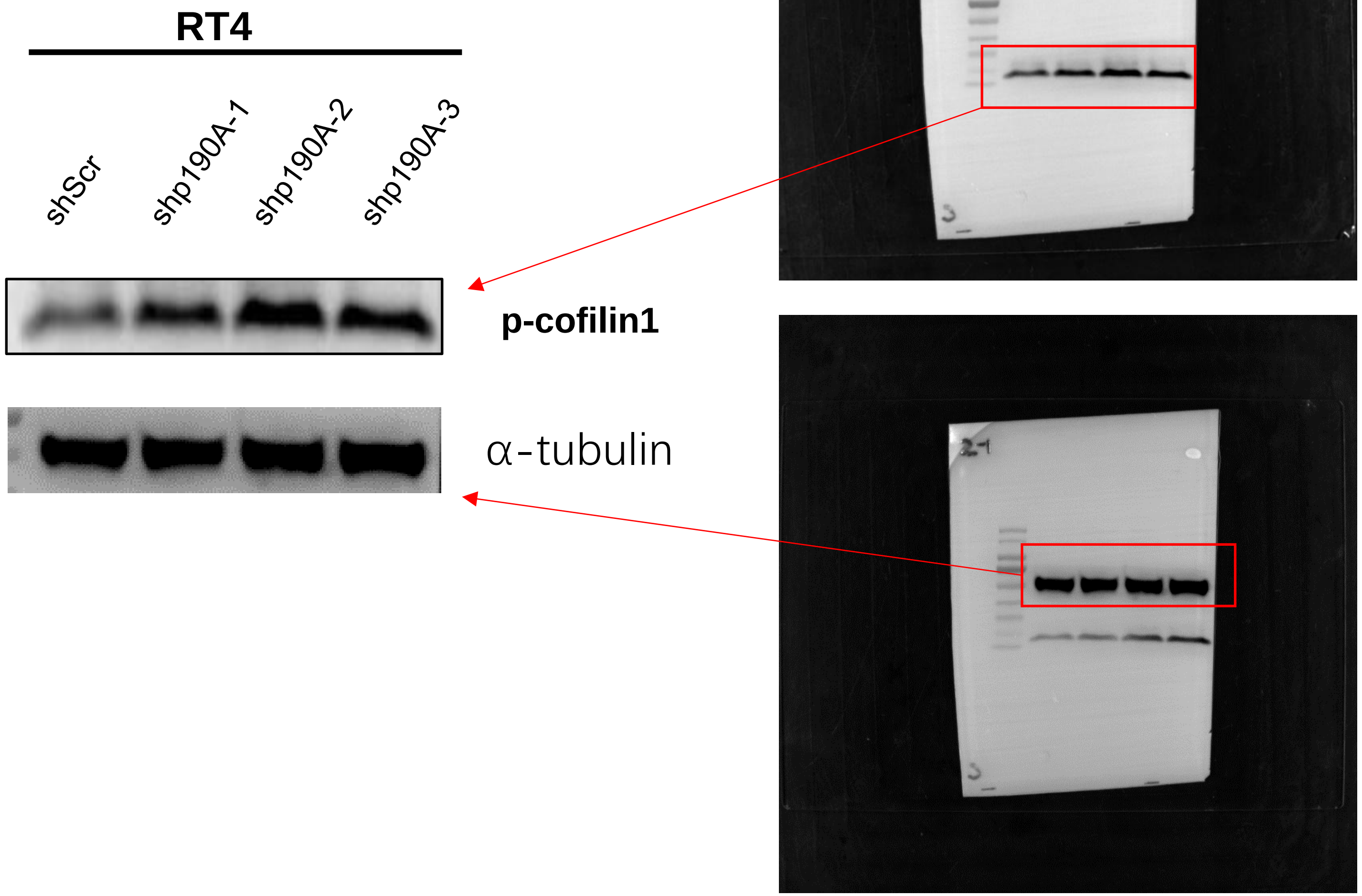

Figure 5A

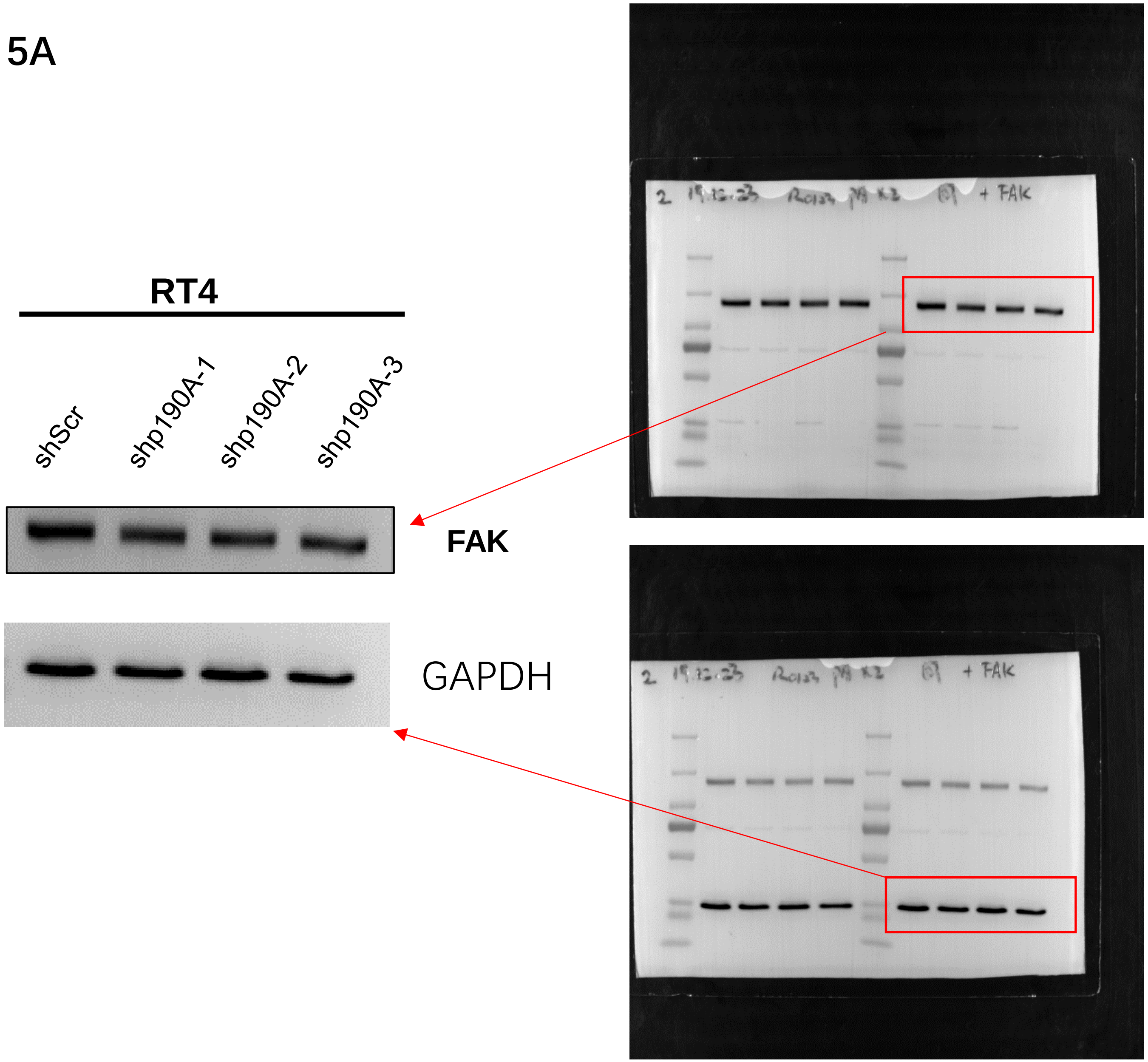

Figure 5A

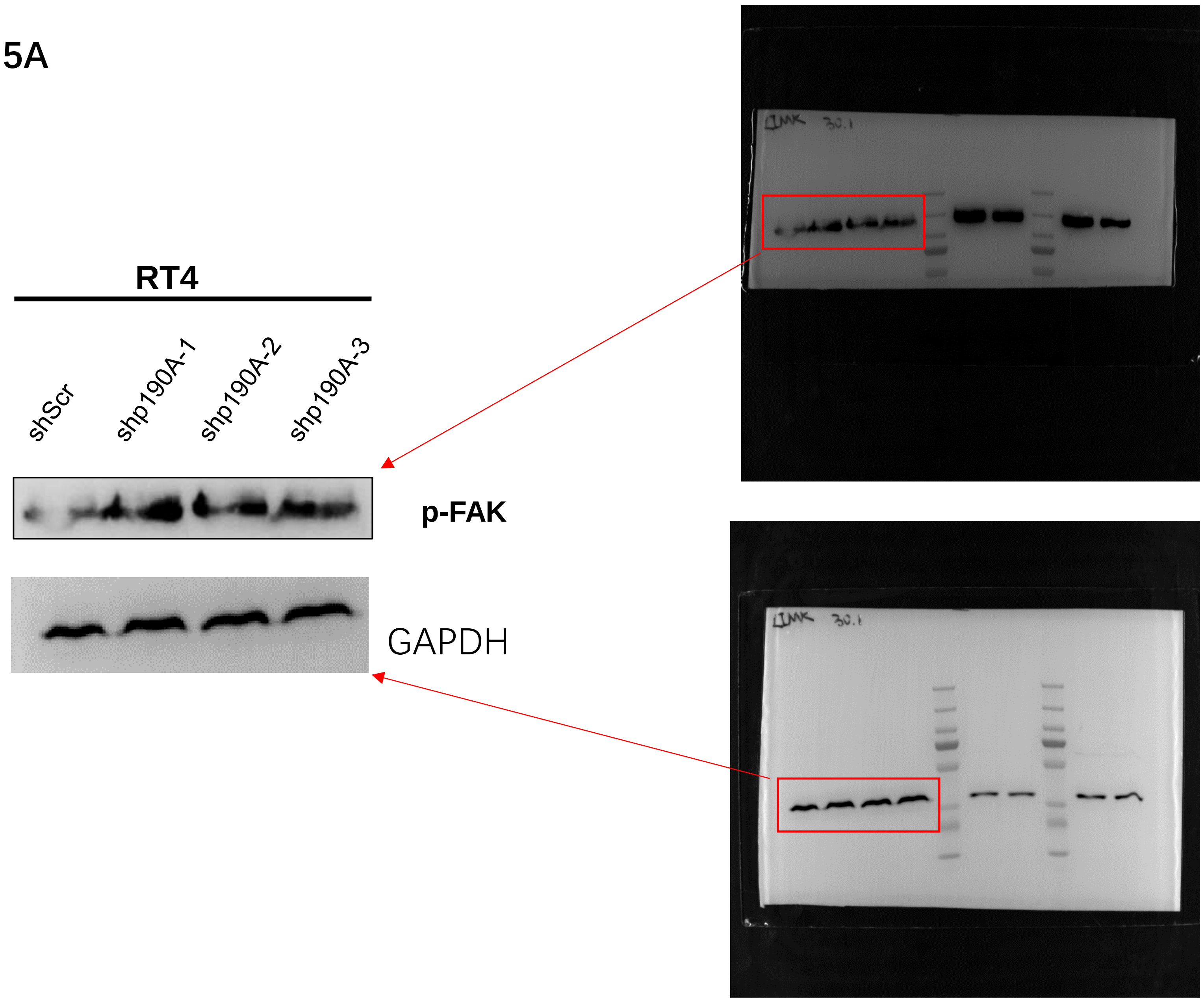

Figure 5A

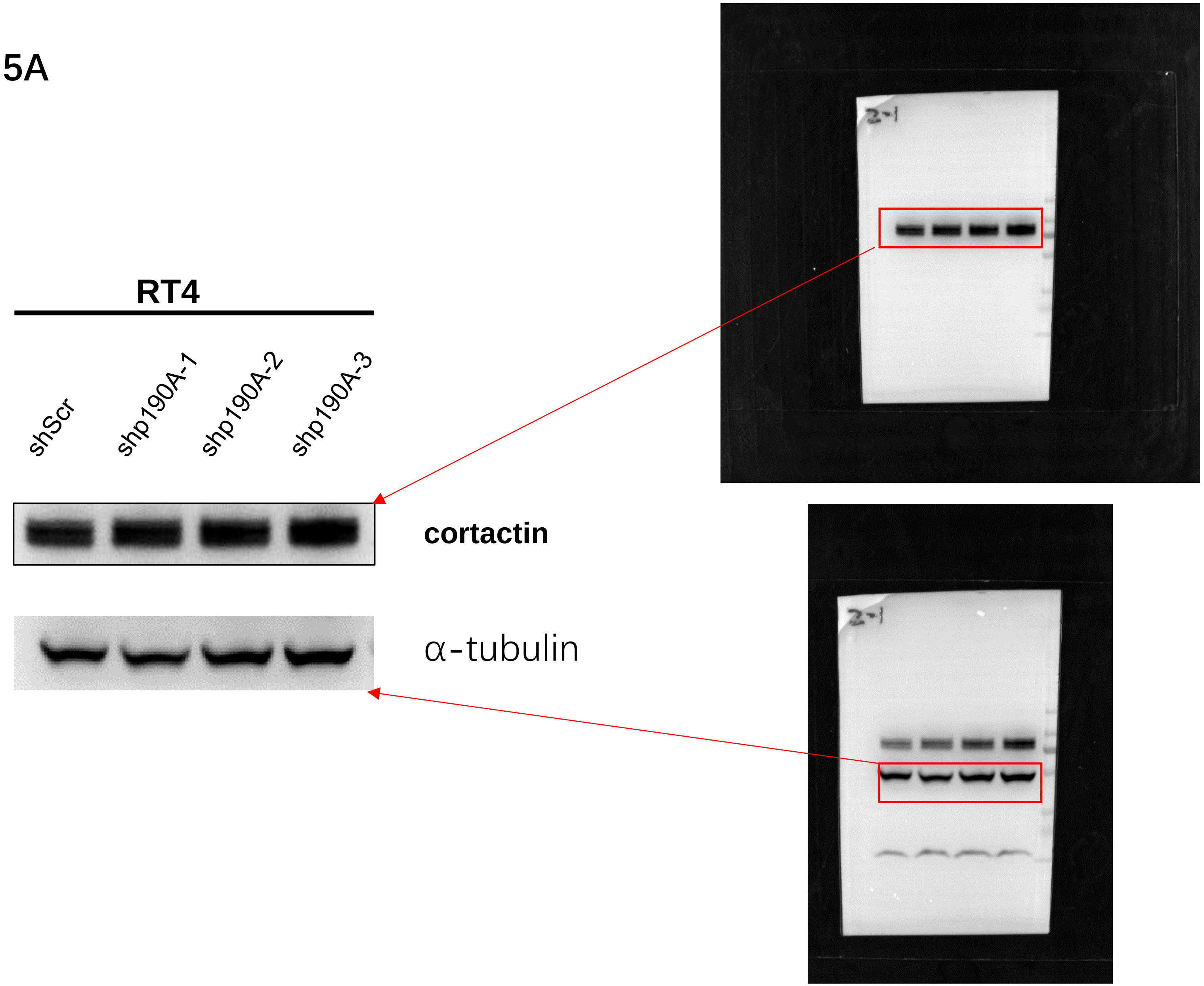

Figure 5A

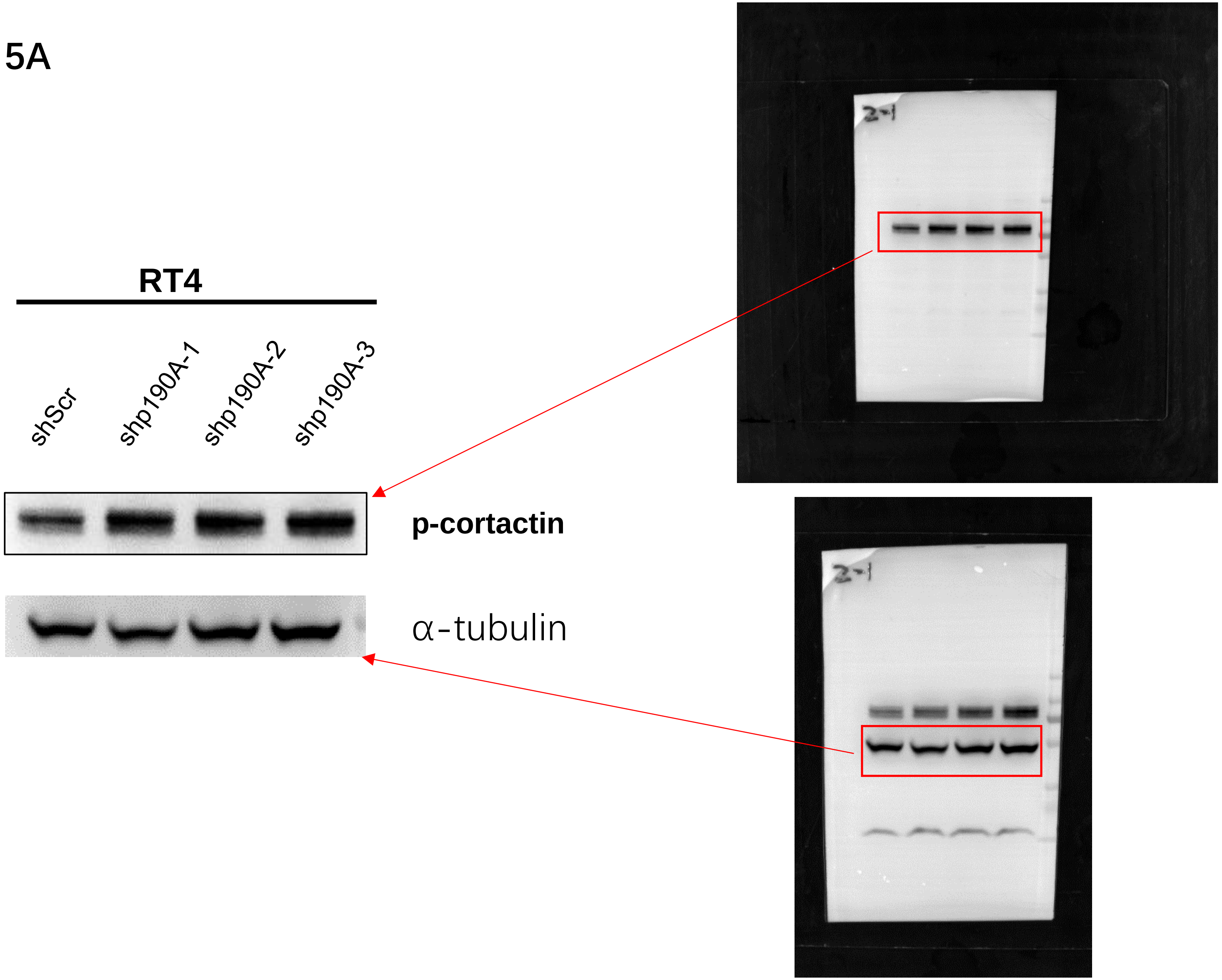

RT4
shScr
shp190A-1
shp190A-2
shp190A-3
p-cortactin
α-tubulin

Figure 5B

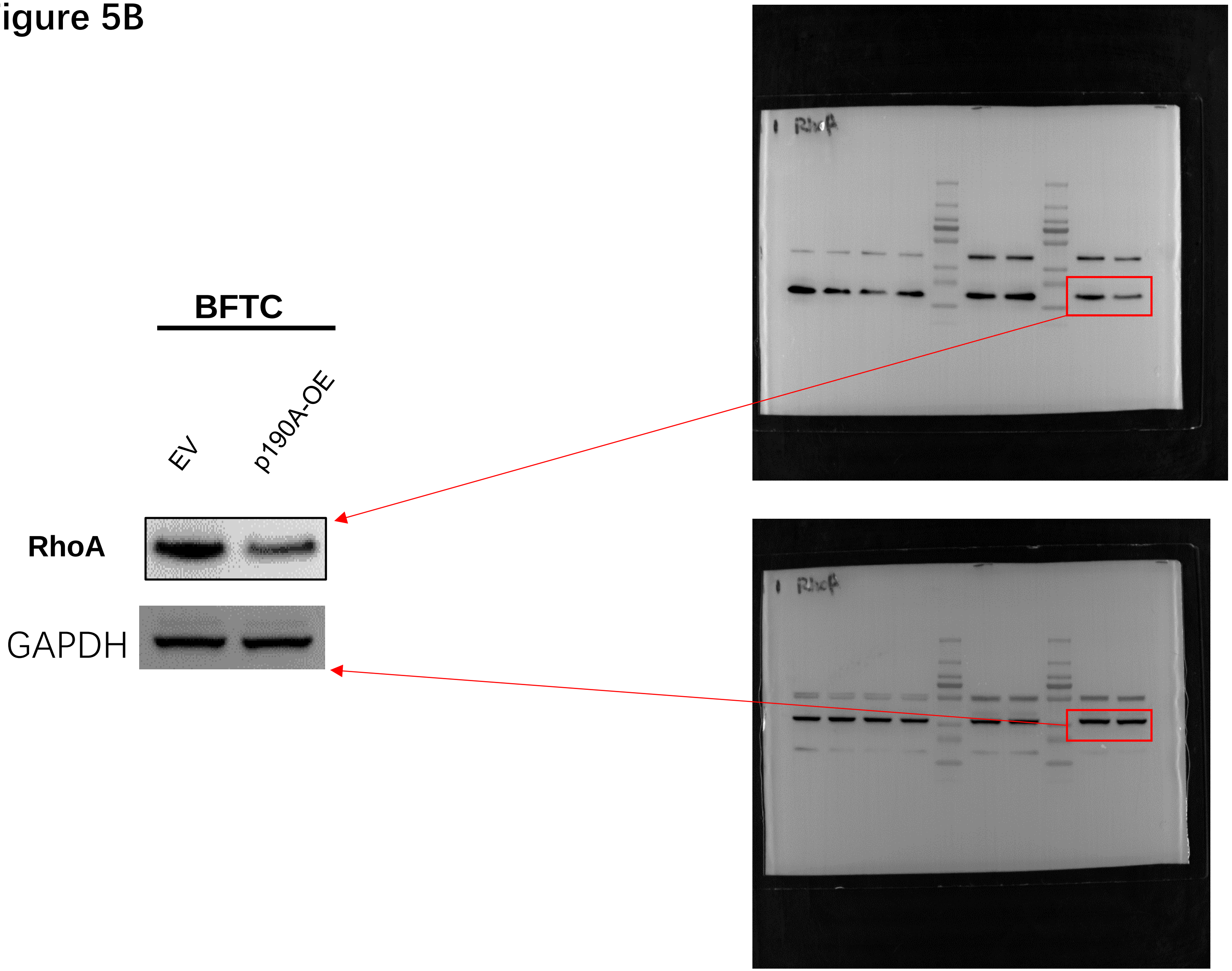

Figure 5B

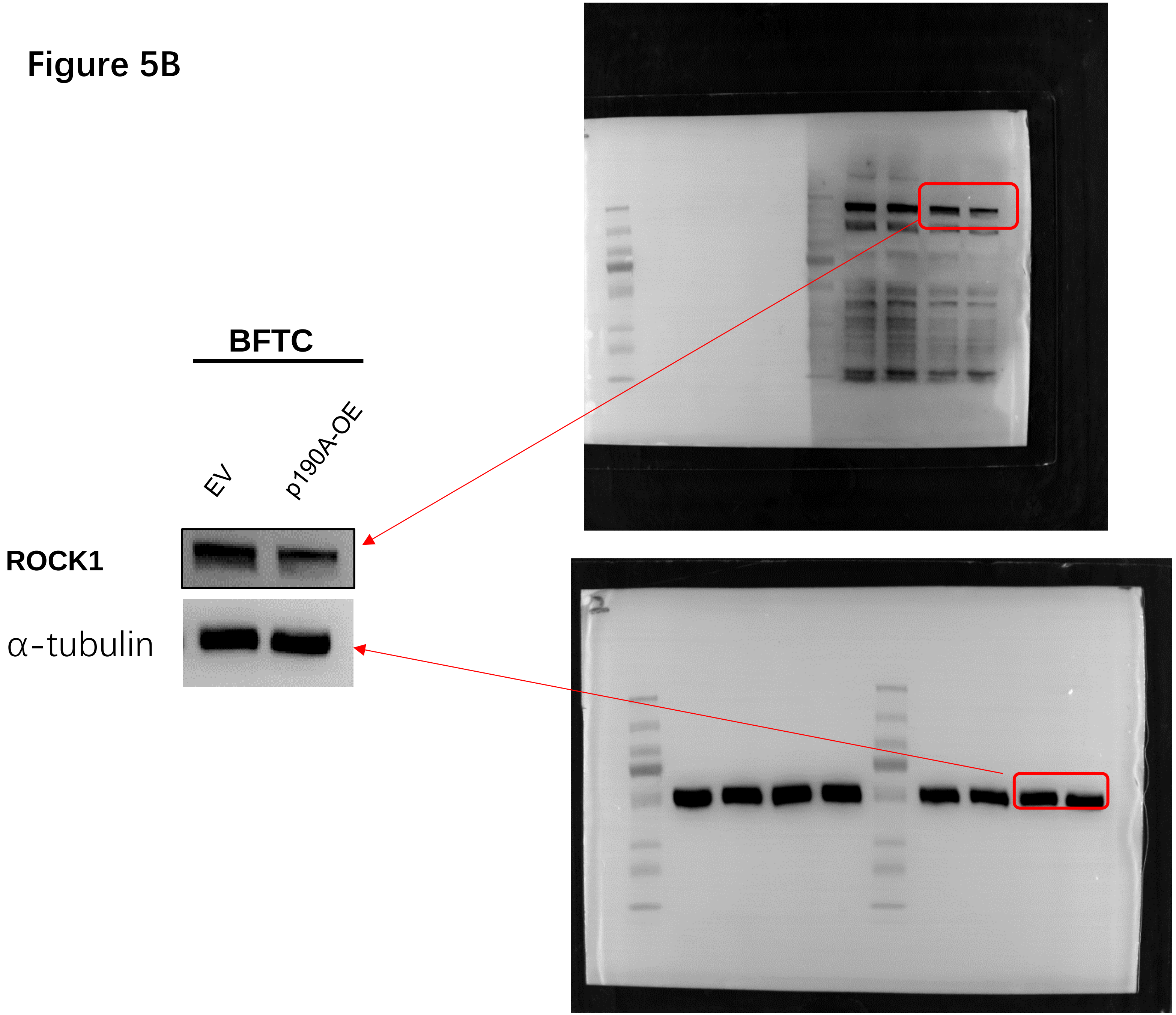

Figure 5B

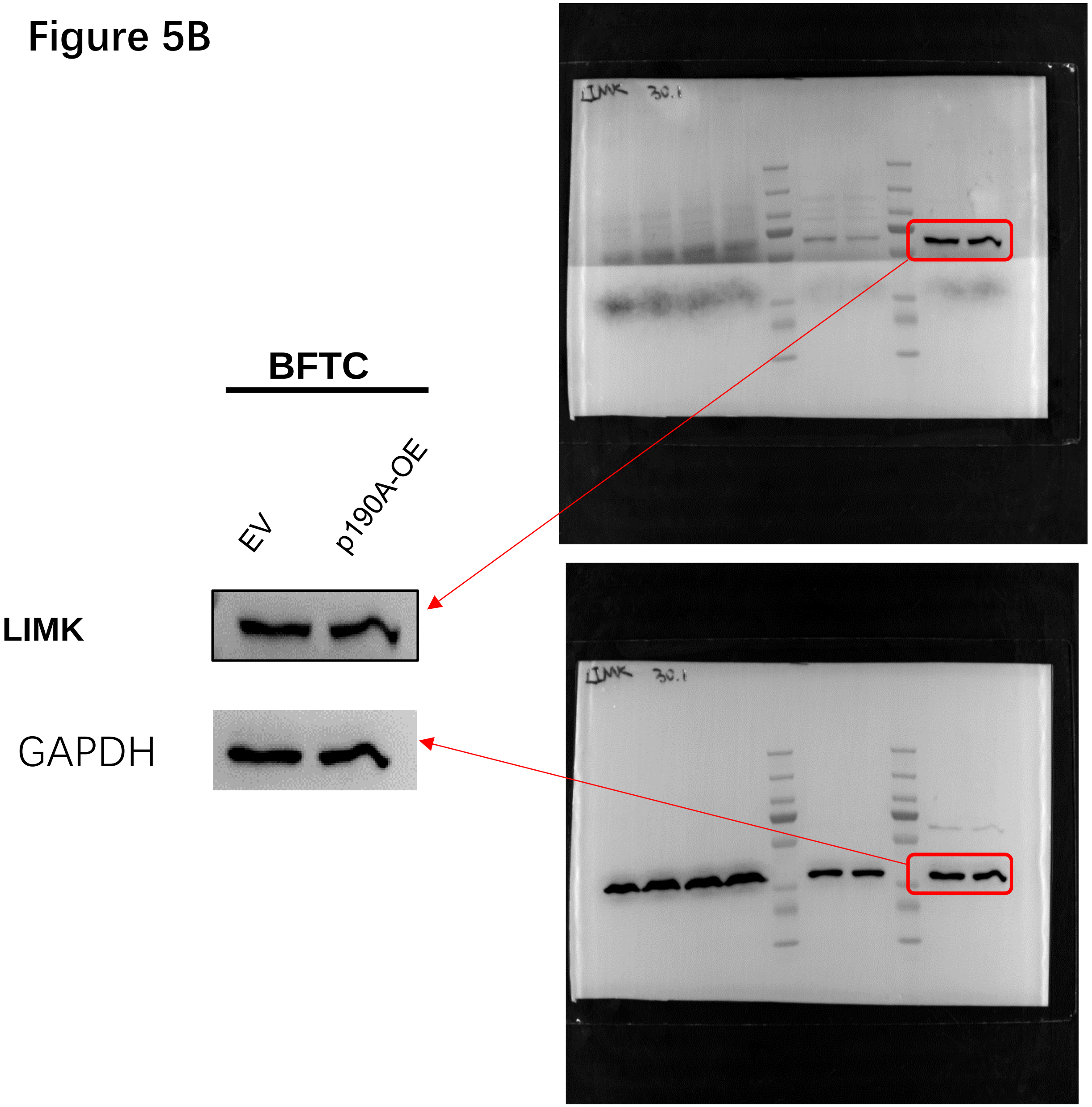

Figure 5B

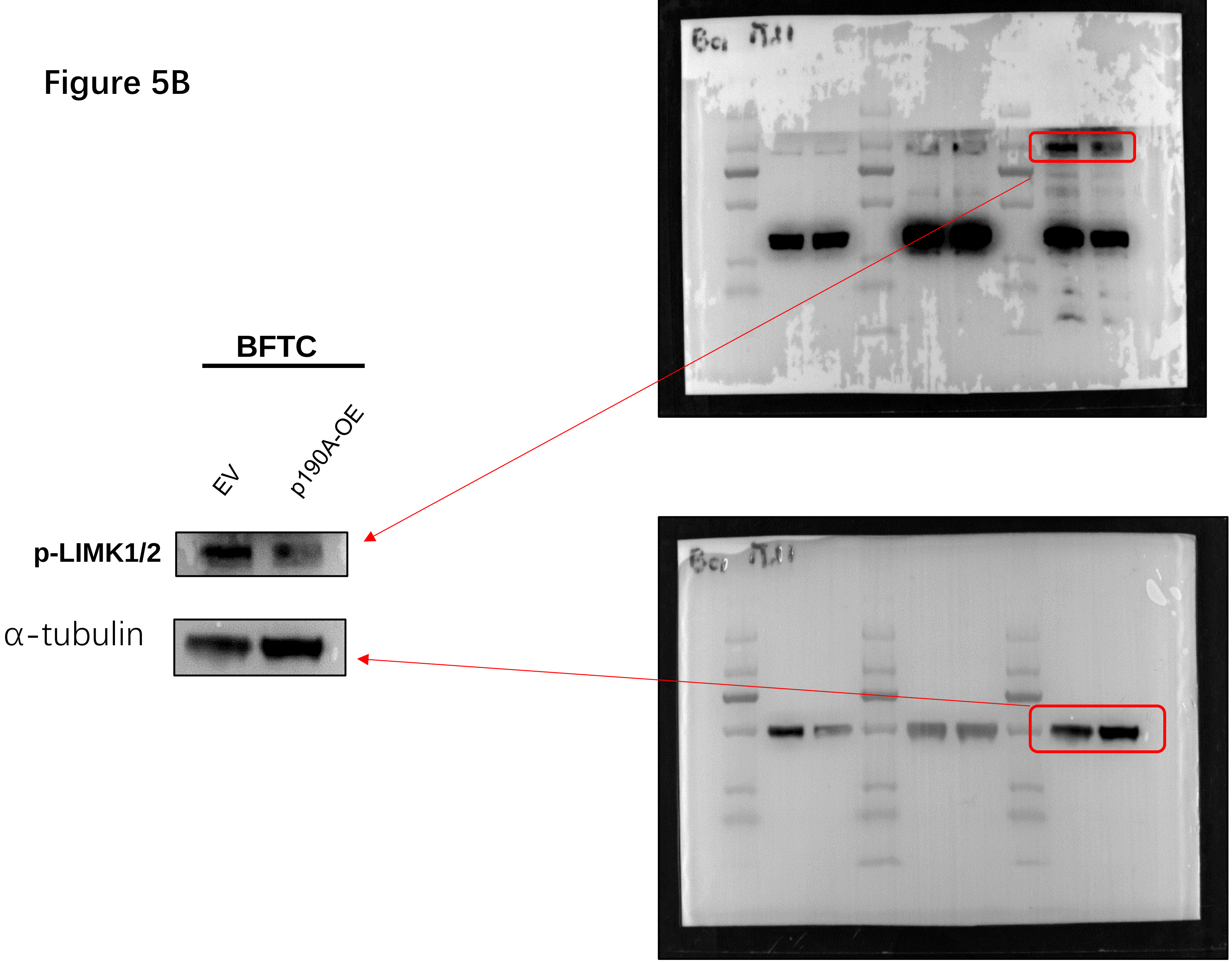

Figure 5B

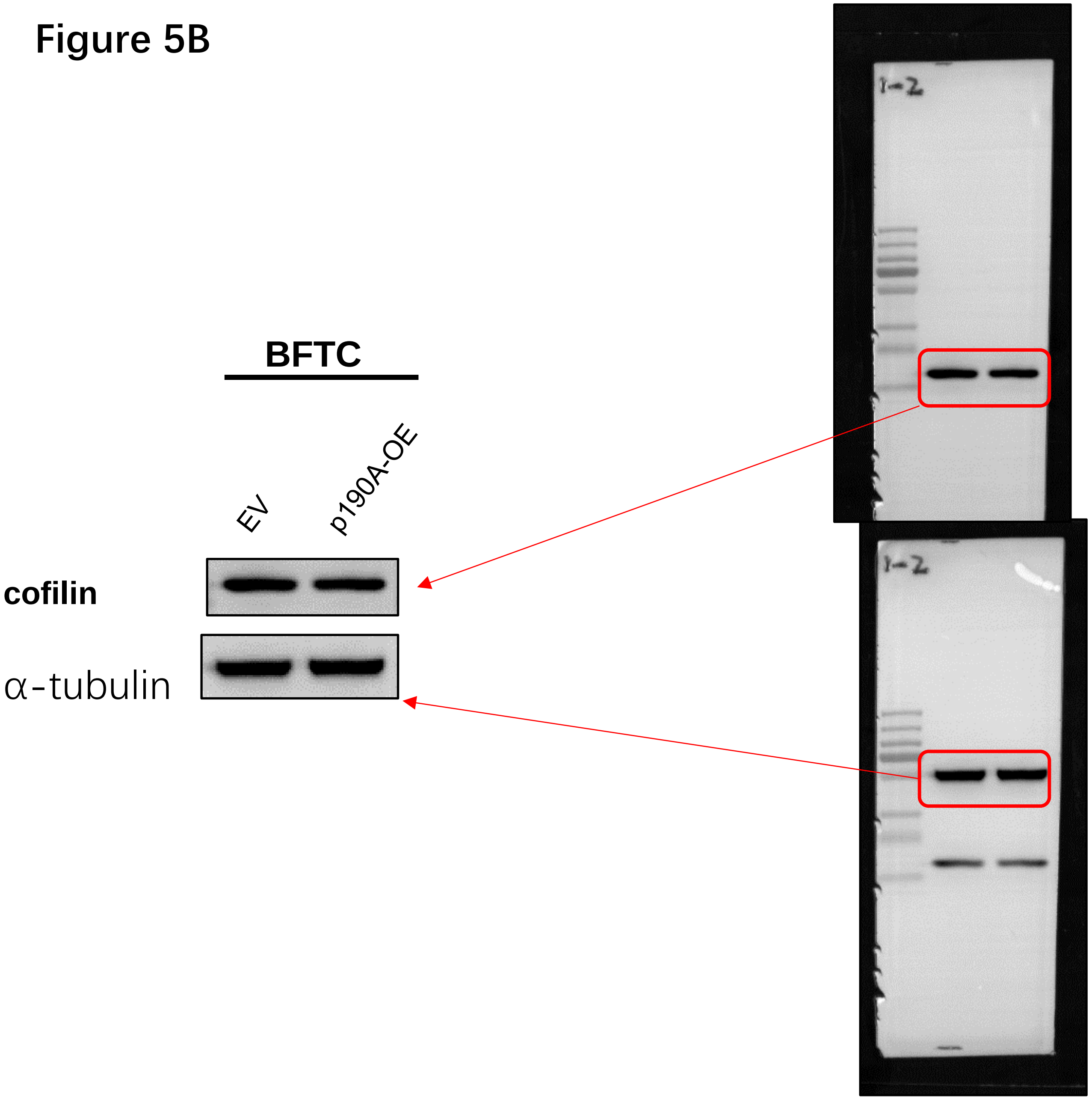

Figure 5B

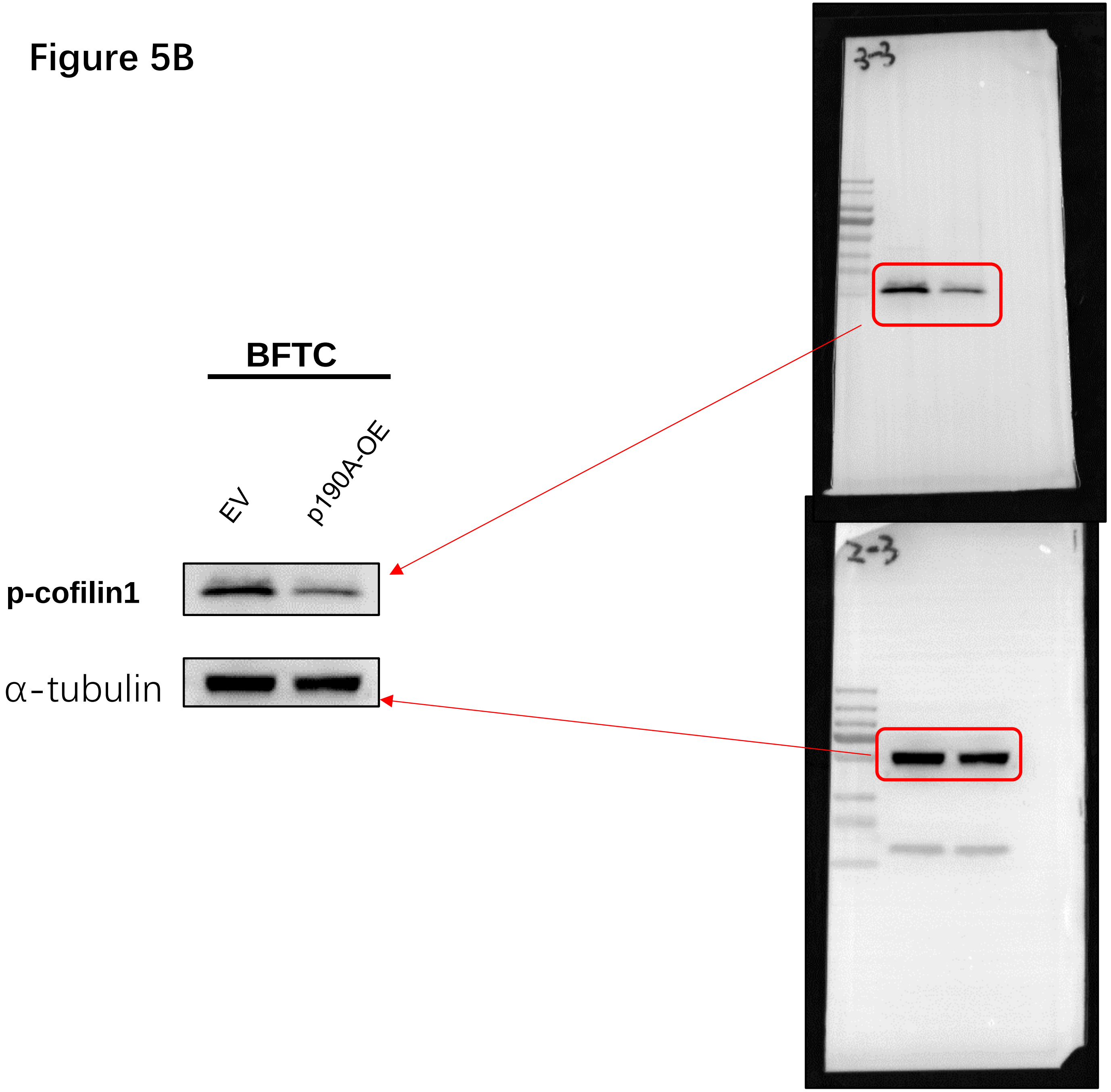

Figure 5B

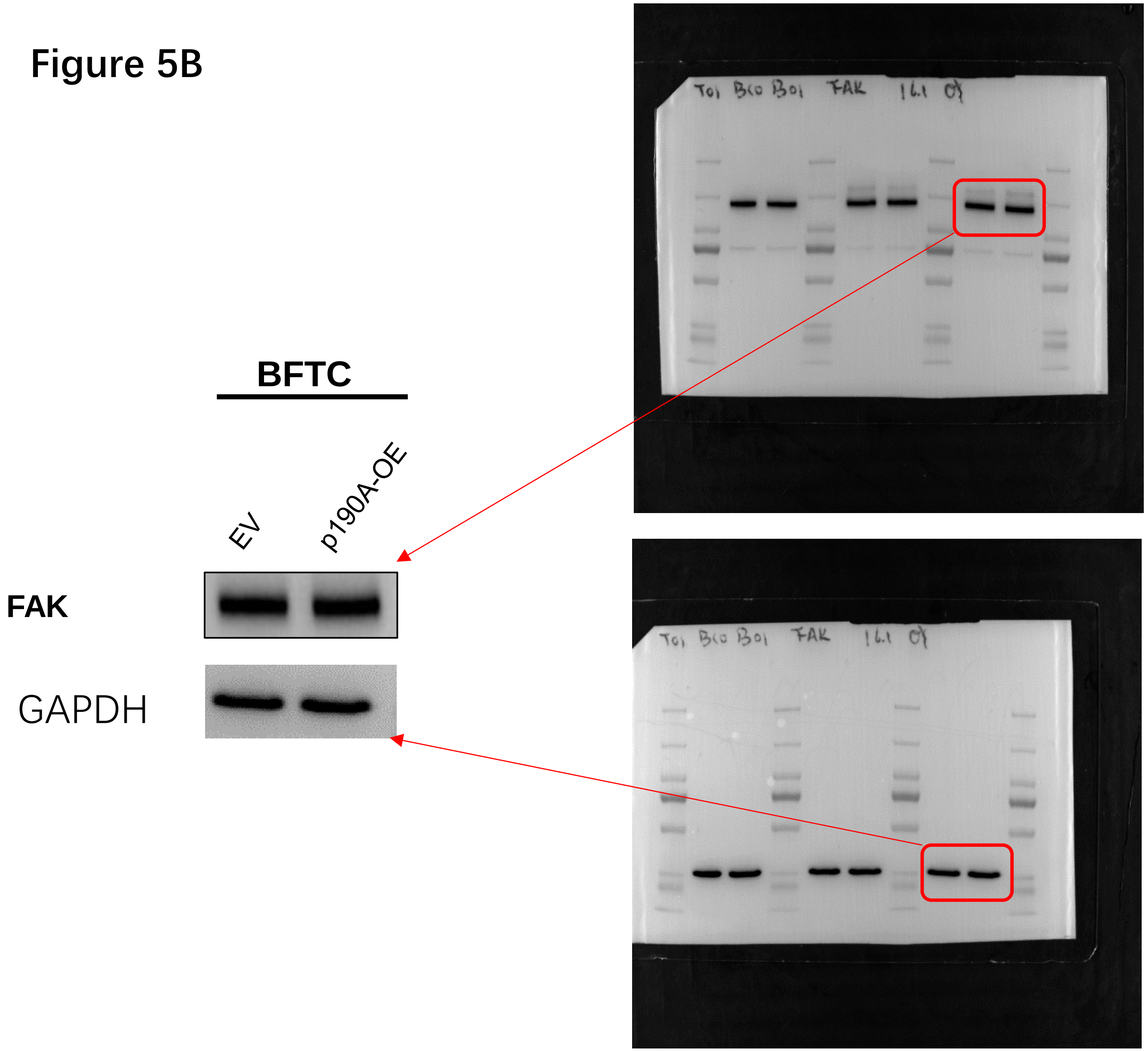

Figure 5B

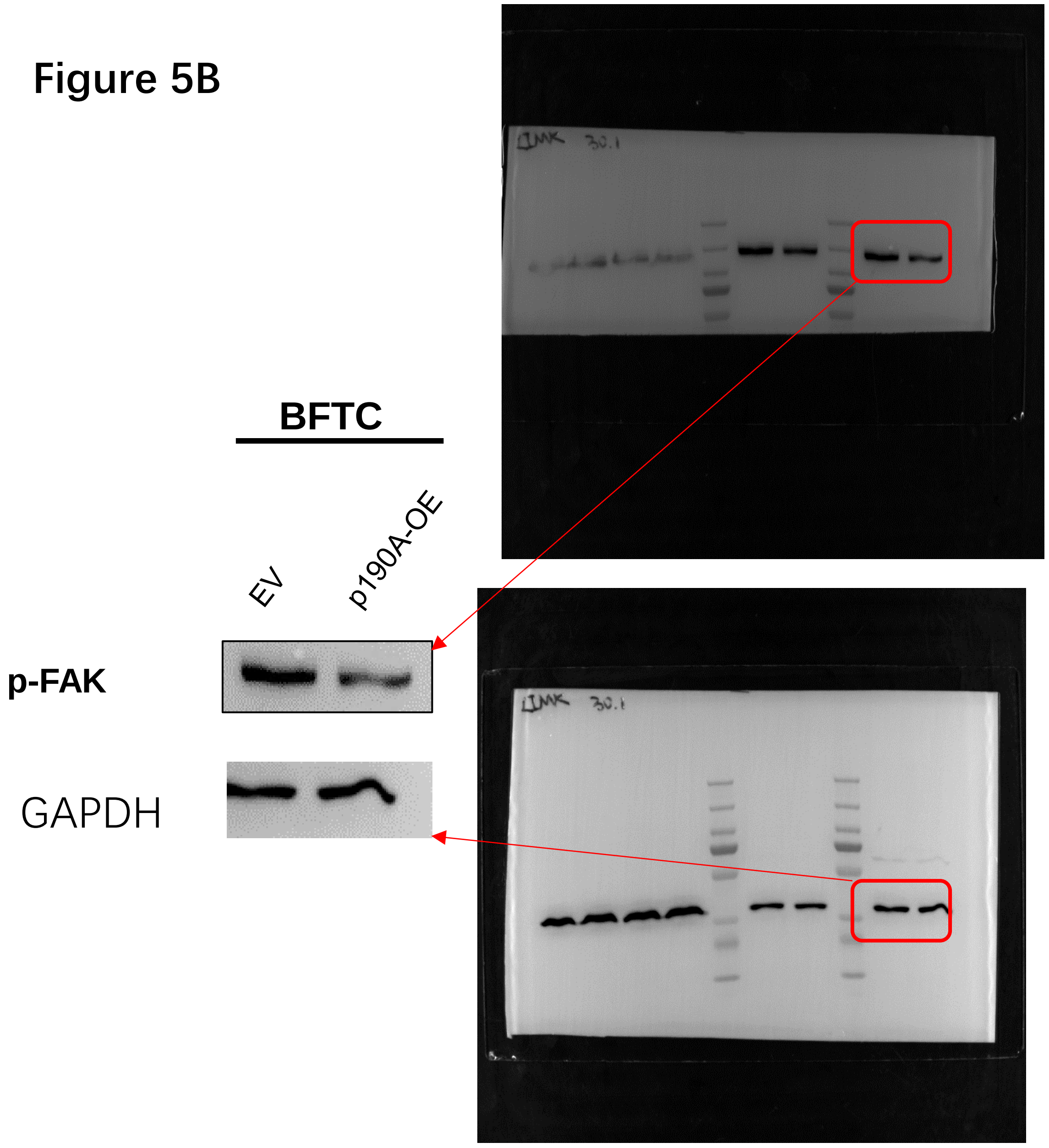

Figure 5B

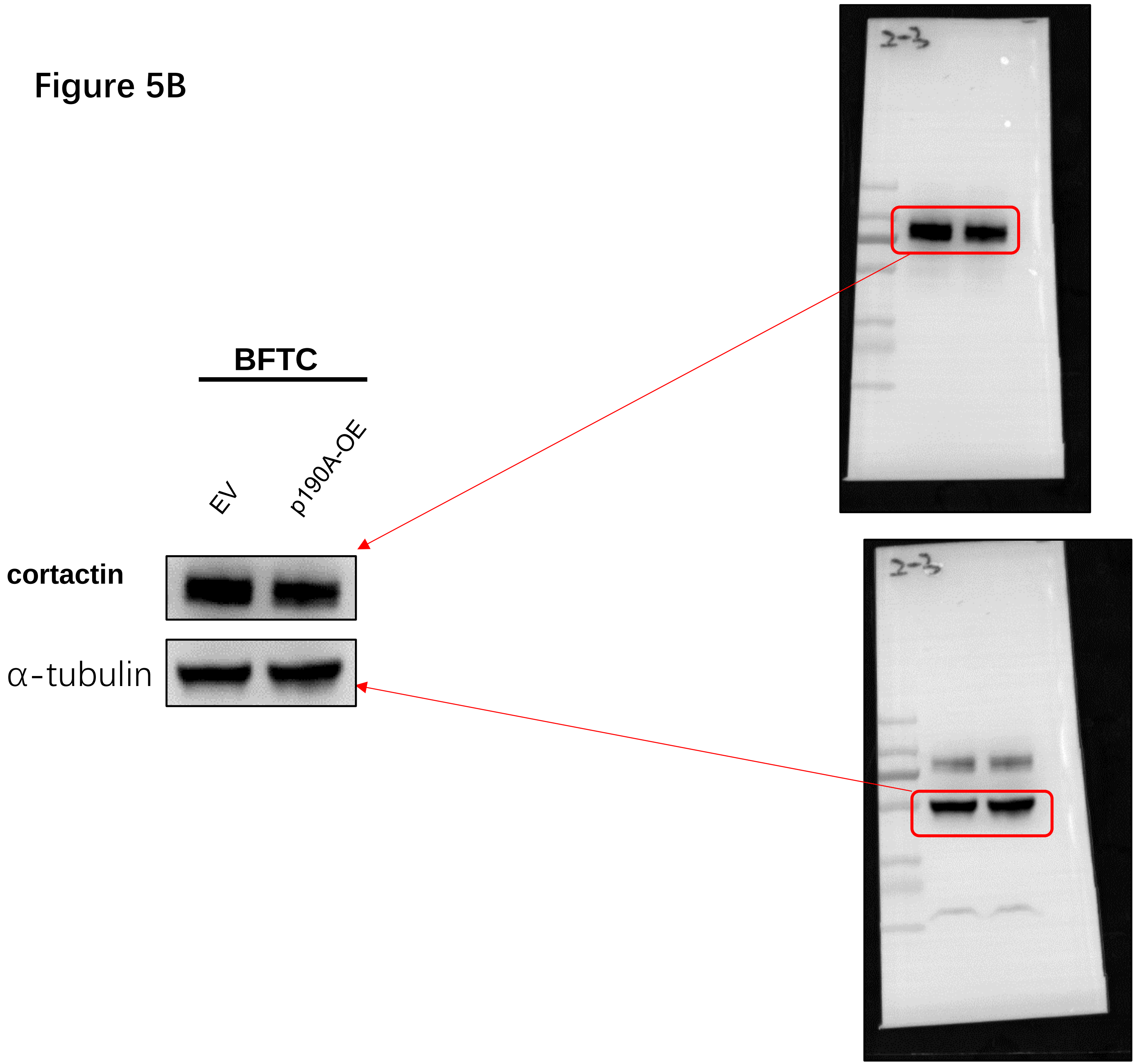

Figure 5B

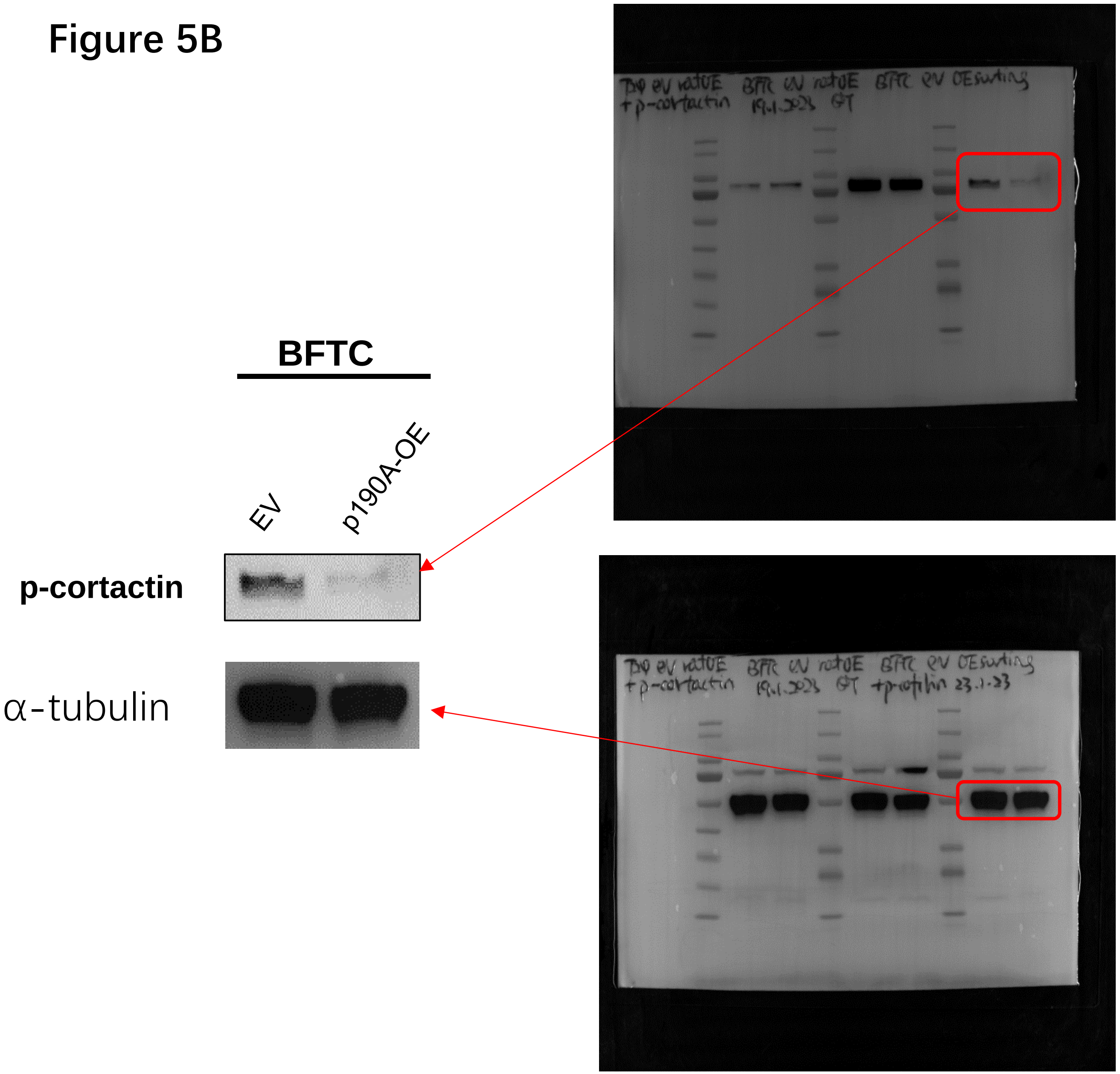

Supplement: Supplementary file 2 — Supplementary Material 2 [file 41598_2025_23687_MOESM2_ESM.pdf]
