# Supplementary material for: Loss of p190A RhoGAP induces aneuploidy and enhances bladder cancer cell migration and invasion by modulating actin dynamics
Source: Sci Rep. 2025 Nov 18;15:40399. doi: 10.1038/s41598-025-23687-4 (PMC12627482; doi:10.1038/s41598-025-23687-4)
Supplement: Supplementary file 3 — Supplementary Material 3 [file 41598_2025_23687_MOESM3_ESM.pdf]

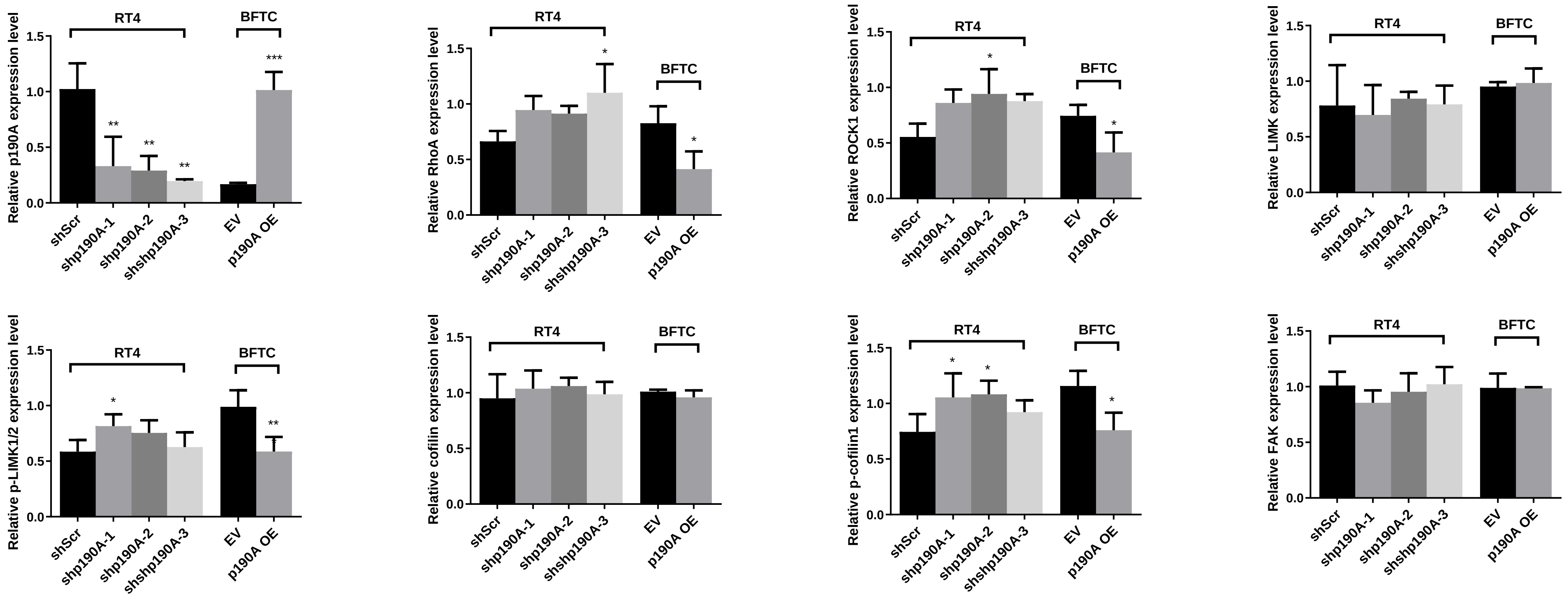

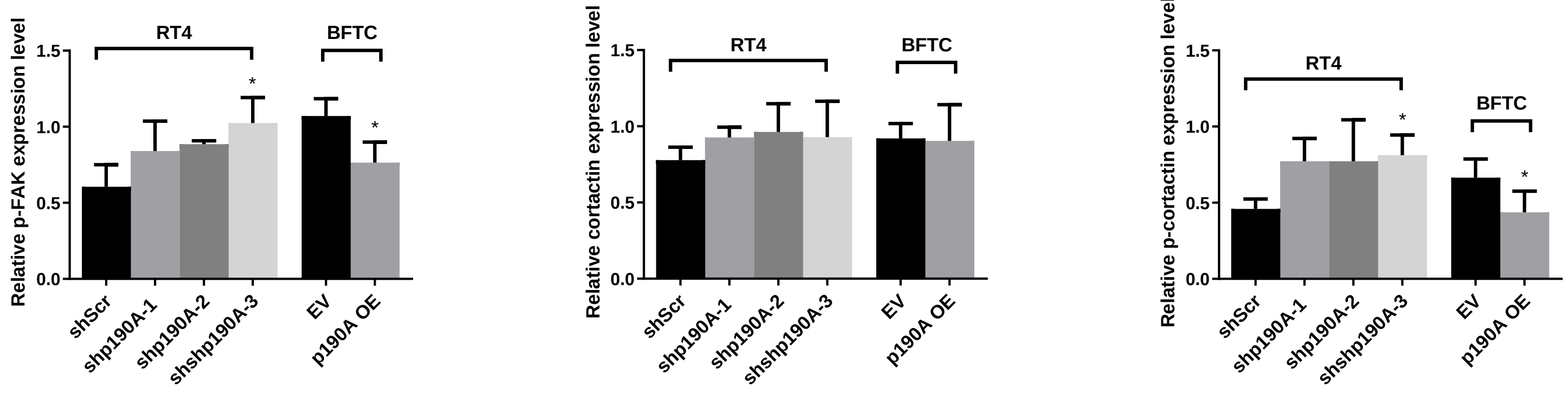

Densitometric analysis of band intensities was performed using ImageJ software. For each protein of interest, the intensity of the band was measured and normalized to the corresponding loading control from the same lane to account for differences in protein loading. Statistical data are presented as mean ± SD (n=3, * $p < 0.05$, ** $p < 0.01$, *** $p < 0.001$. (RT4) ANOVA, (BFTC) Student’s t-test).
