# Supplementary figures and images for: Loss of p190A RhoGAP induces aneuploidy and enhances bladder cancer cell migration and invasion by modulating actin dynamics
Source: Sci Rep. 2025 Nov 18;15:40399. doi: 10.1038/s41598-025-23687-4 (PMC12627482; doi:10.1038/s41598-025-23687-4)

# RT4

shScr

shp190A-1

shp190A-2

Anti-HLA

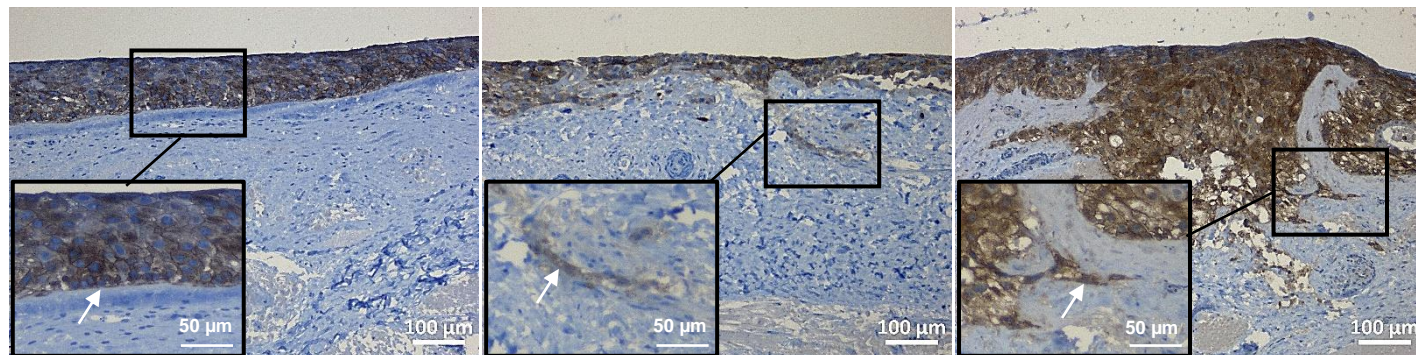

Anti-p190A

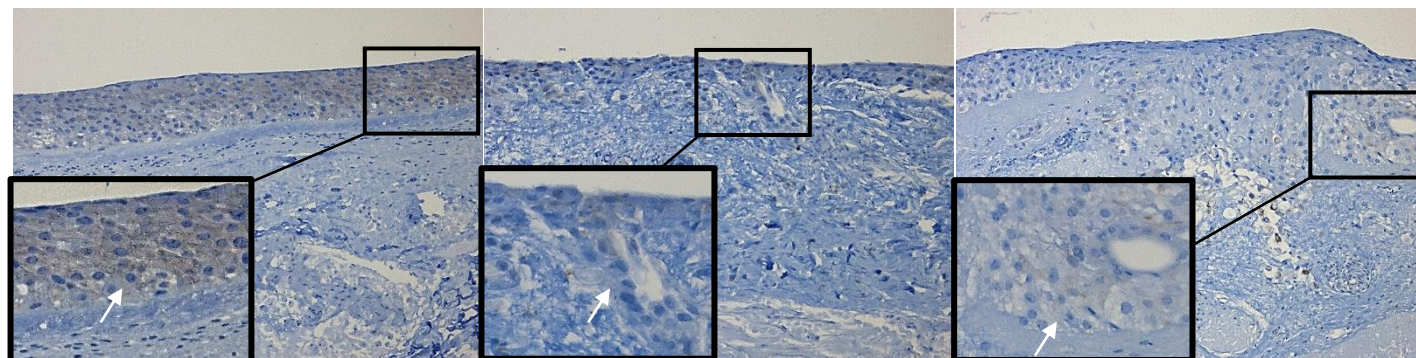

## RT4

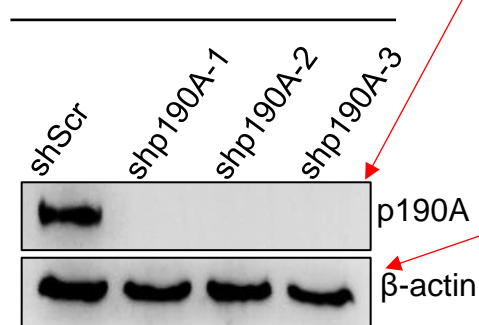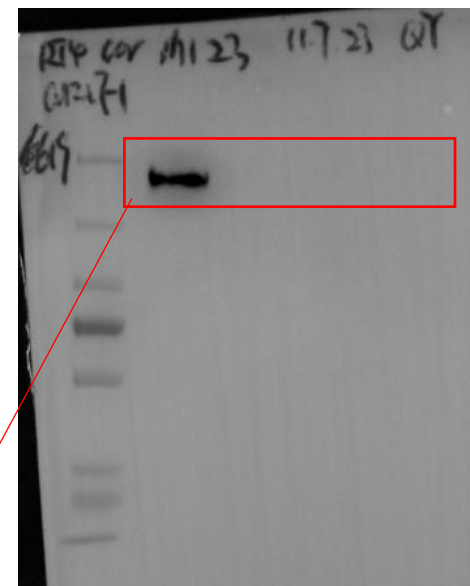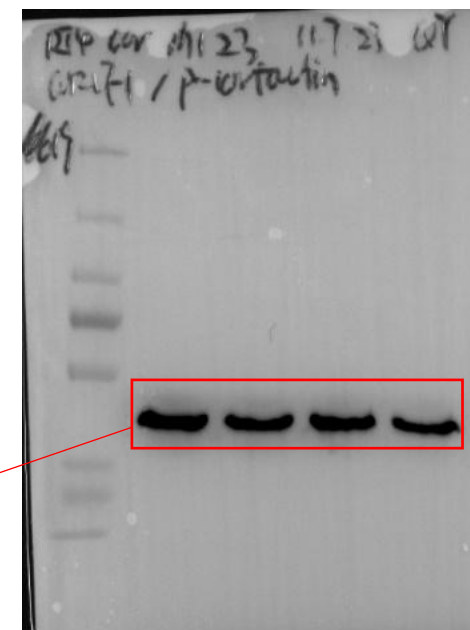

Supplement: Supplementary file 4 — Supplementary Material 4 [file 41598_2025_23687_MOESM4_ESM.pdf]
