# Supplementary material for: Loss of p190A RhoGAP induces aneuploidy and enhances bladder cancer cell migration and invasion by modulating actin dynamics
Source: Sci Rep. 2025 Nov 18;15:40399. doi: 10.1038/s41598-025-23687-4 (PMC12627482; doi:10.1038/s41598-025-23687-4)
Supplement: Supplementary file 5 — Supplementary Material 5 [file 41598_2025_23687_MOESM5_ESM.pdf]

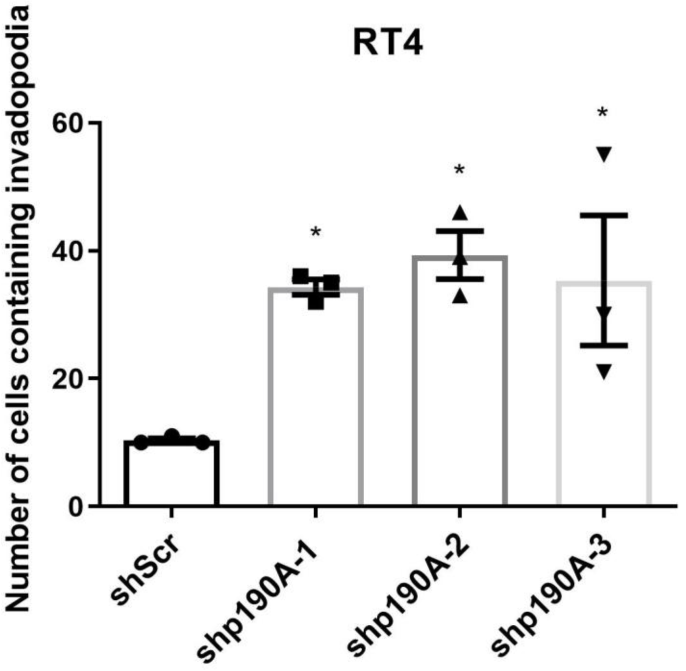

Supplementary Figure 5. Knockdown of p190A increases the number of RT4 cells containing invadopodia. RT4 bladder cancer cells were transduced with scrambled shRNA (shScr) or three independent shRNAs targeting p190A (shp190A-1, shp190A-2, shp190A-3). The number of cells containing invadopodia was quantified. All three p190A knockdown constructs showed a significant increase in invadopodia-containing cells compared to control. Quantification was based on the results of three independent experiments, and results are presented as mean ± SEM. ANOVA were used for the statistical analyses. (* $p < 0.05$).
