# Supplementary material for: Loss of p190A RhoGAP induces aneuploidy and enhances bladder cancer cell migration and invasion by modulating actin dynamics
Source: Sci Rep. 2025 Nov 18;15:40399. doi: 10.1038/s41598-025-23687-4 (PMC12627482; doi:10.1038/s41598-025-23687-4)
Supplement: Supplementary file 6 — Supplementary Material 6 [file 41598_2025_23687_MOESM6_ESM.pdf]

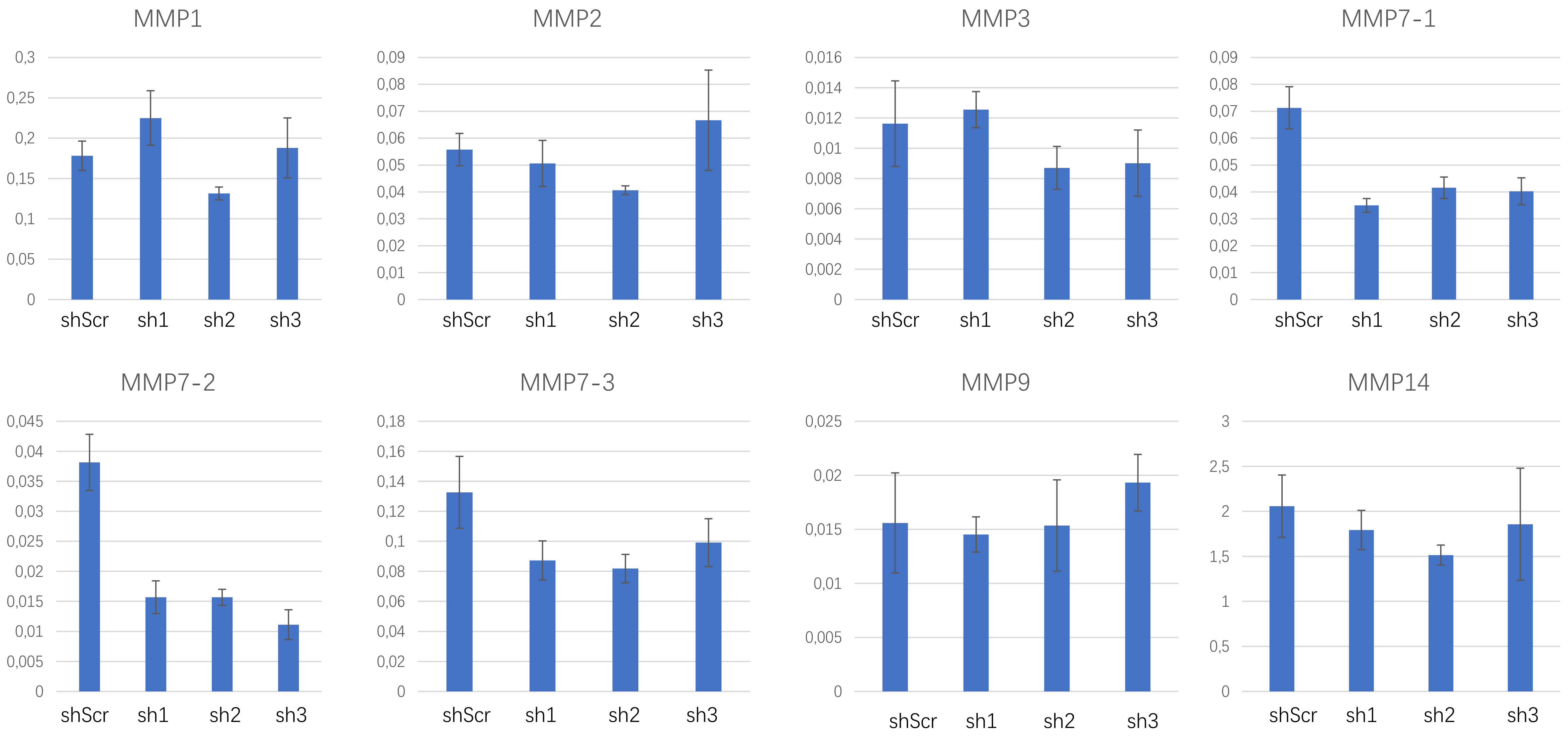

Supplementary Figure 6-A. Expression of MMPs in RT4 cells in relation to p190A knockdown.
Quantitative RT-PCR analysis to evaluate relative mRNA levels of different matrix metalloproteases (MMPs) in RT4 cells with scrambled control (shScr) or p190A-targeting shRNAs (sh1, sh2, sh3).
Data are presented as fold change relative to shScr control and normalized to GAPDH expression.

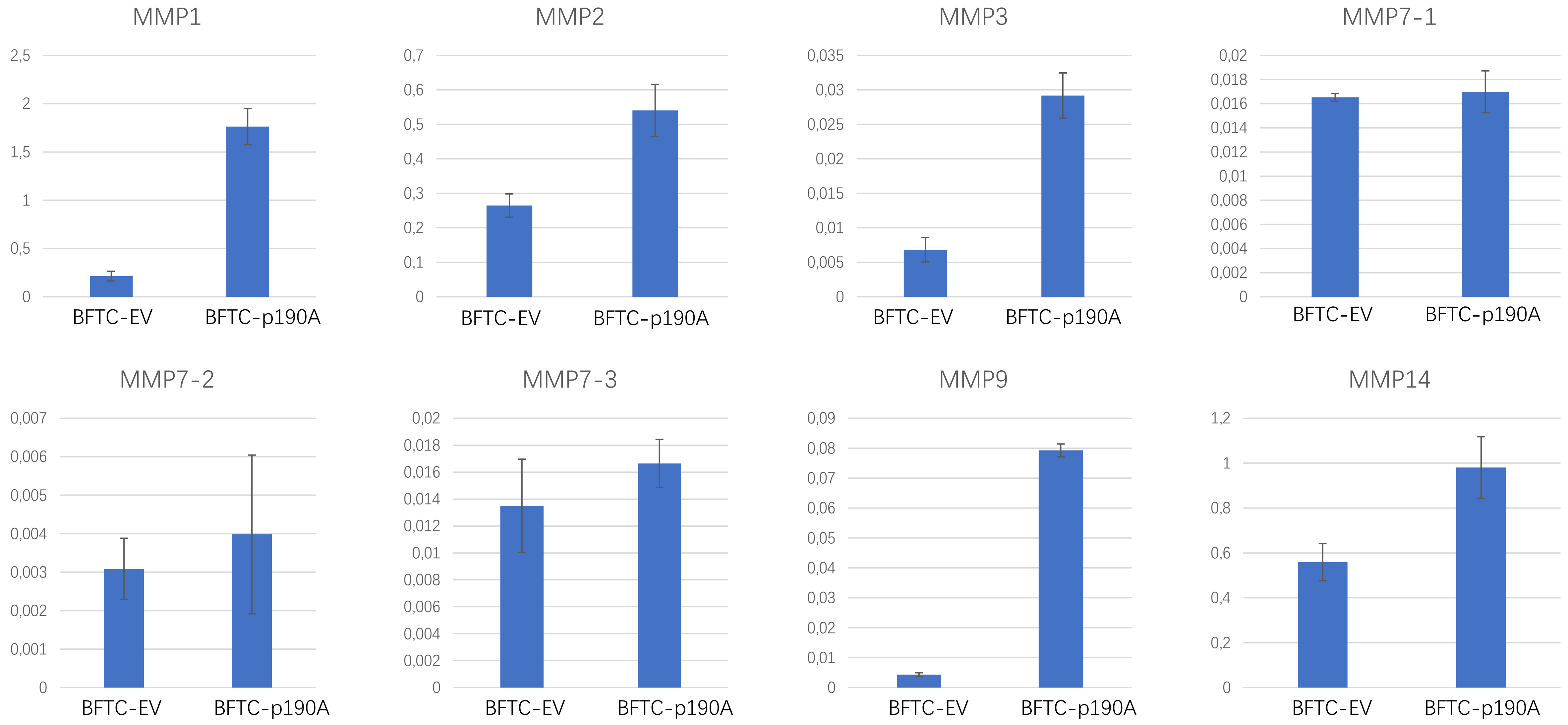

Supplementary Figure 6-B. Expression of MMPs in BFTC cells in relation to p190A overexpression.
Quantitative RT-PCR analysis to evaluate relative mRNA levels of different matrix metalloproteases (MMPs) in BFTC cells overexpressing p190A (p190A-OE) compared to empty vector control (EV).
Data are presented as fold change relative to EV control and normalized to GAPDH expression.

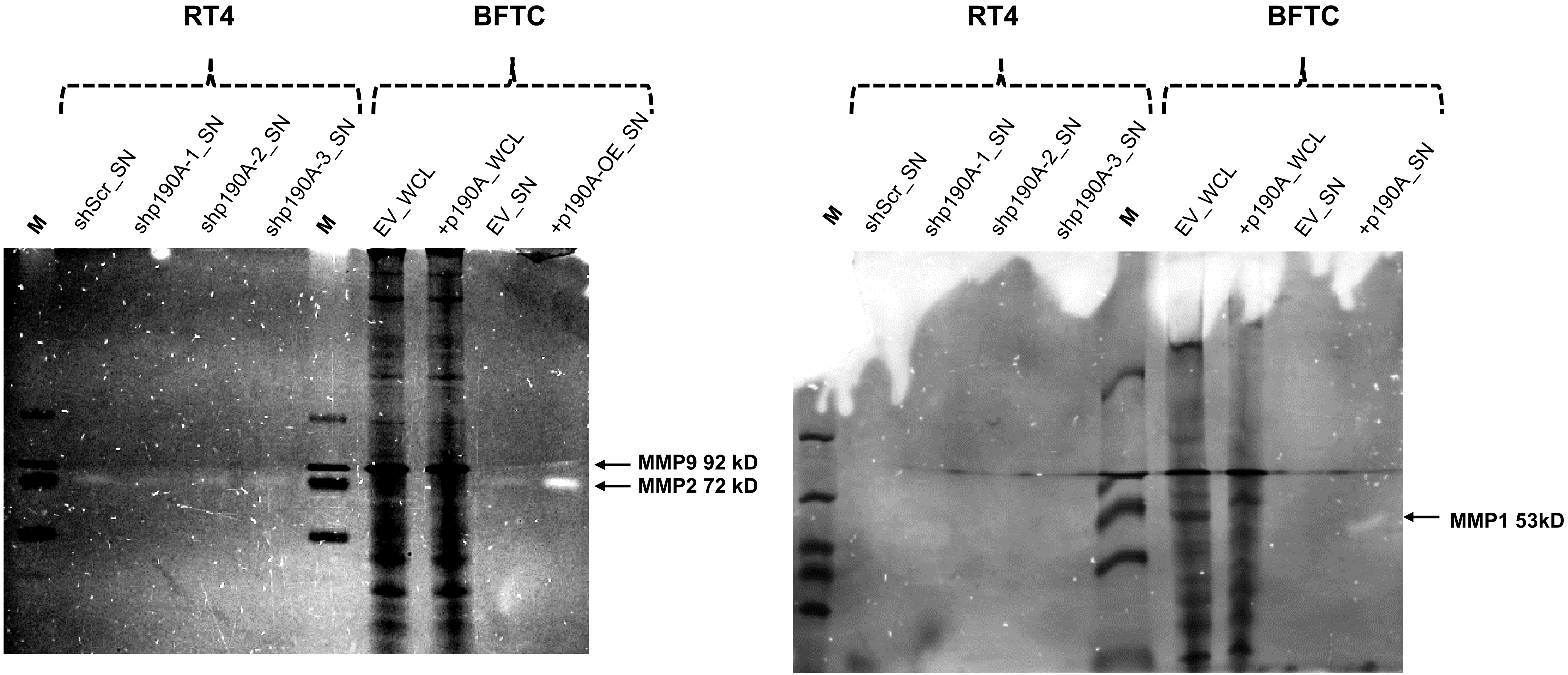

Supplementary Figure 6-C. Gelatin and collagen zymography analyses for MMP2, MMP9, and MMP1 activity in RT4 and BFTC cell lines.
Left panel (gelatin zymography): Using cell culture supernatant (SN) from RT4 cells with a control shRNA (shScr) or RT4 cells with shRNAs targeting p190A (shp190A1–3), MMP2 (72 kDa) and MMP9 (92kDa) activities were visualized as gelatin degradion areas (white bands in dark background). Similarly, BFTC cell culture SNs of BFTC cells with an empty vector (EV) or with an ectopic p190A were used to visualize MMP2 and MMP9 (92 kDa) activities. **Right panel (collagen zymography):** Using cell culture supernatant (SN) from RT4 cells with a control shRNA (shScr) or RT4 cells with shRNAs targeting p190A (shp190A1–3), MMP1 (53 kDa) activity was visualized as collagen degradion areas. Similarly, BFTC cell culture supernatants (SN) or BFTC whole cell lysates (WCL) with an empty vector (EV) or with an ectopic p190A were used to visualize MMP1 (53 kDa) activity (white bands in dark background).
