# Supplementary material for: Loss of p190A RhoGAP induces aneuploidy and enhances bladder cancer cell migration and invasion by modulating actin dynamics
Source: Sci Rep. 2025 Nov 18;15:40399. doi: 10.1038/s41598-025-23687-4 (PMC12627482; doi:10.1038/s41598-025-23687-4)
Supplement: Supplementary file 7 — Supplementary Material 7 [file 41598_2025_23687_MOESM7_ESM.pdf]

Figure 1

Figure 1C-Y235T

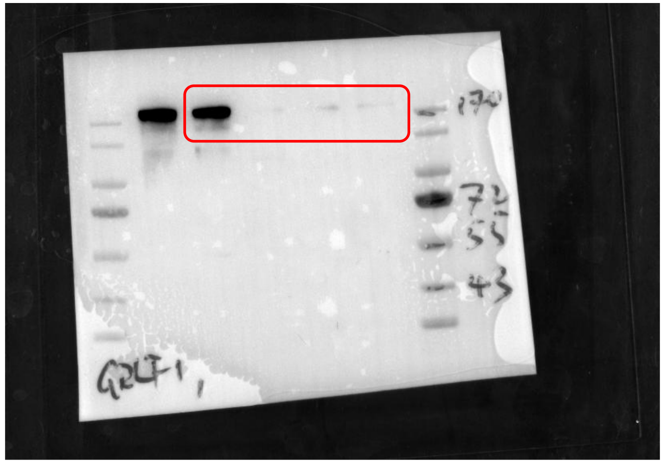

p-190A

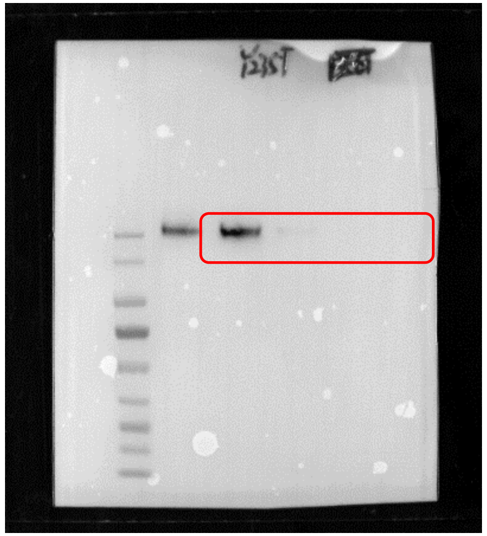

p-190A

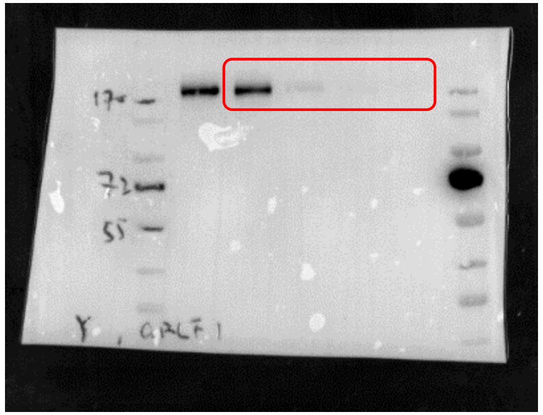

p-190A

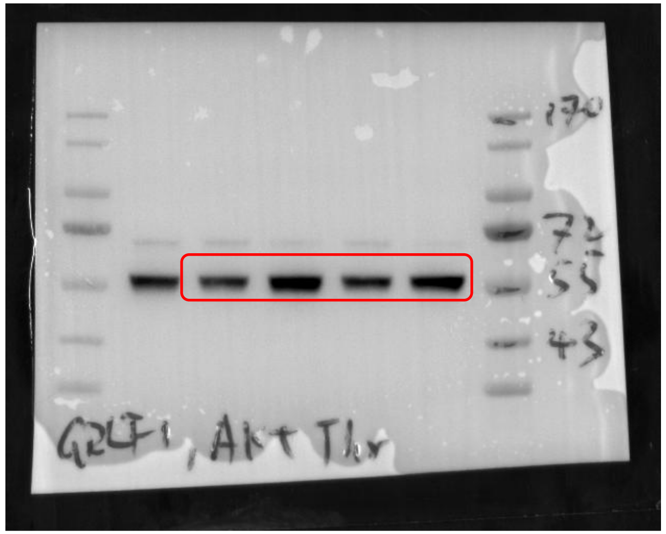

α-tubulin

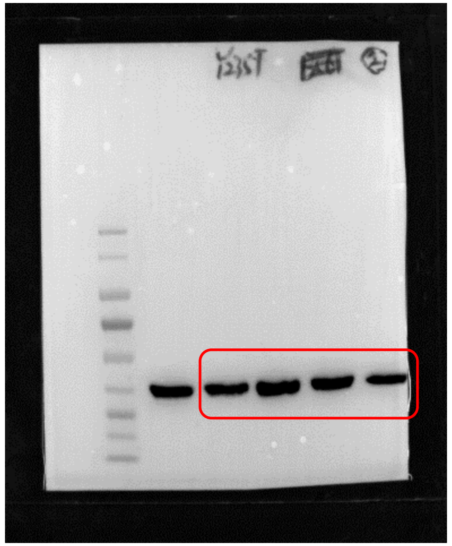

β-actin

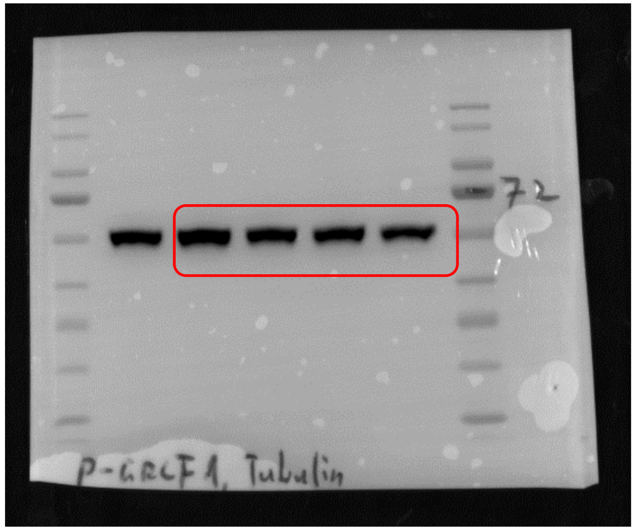

a-tubulin

Figure 3

Figure 3A-cell lines

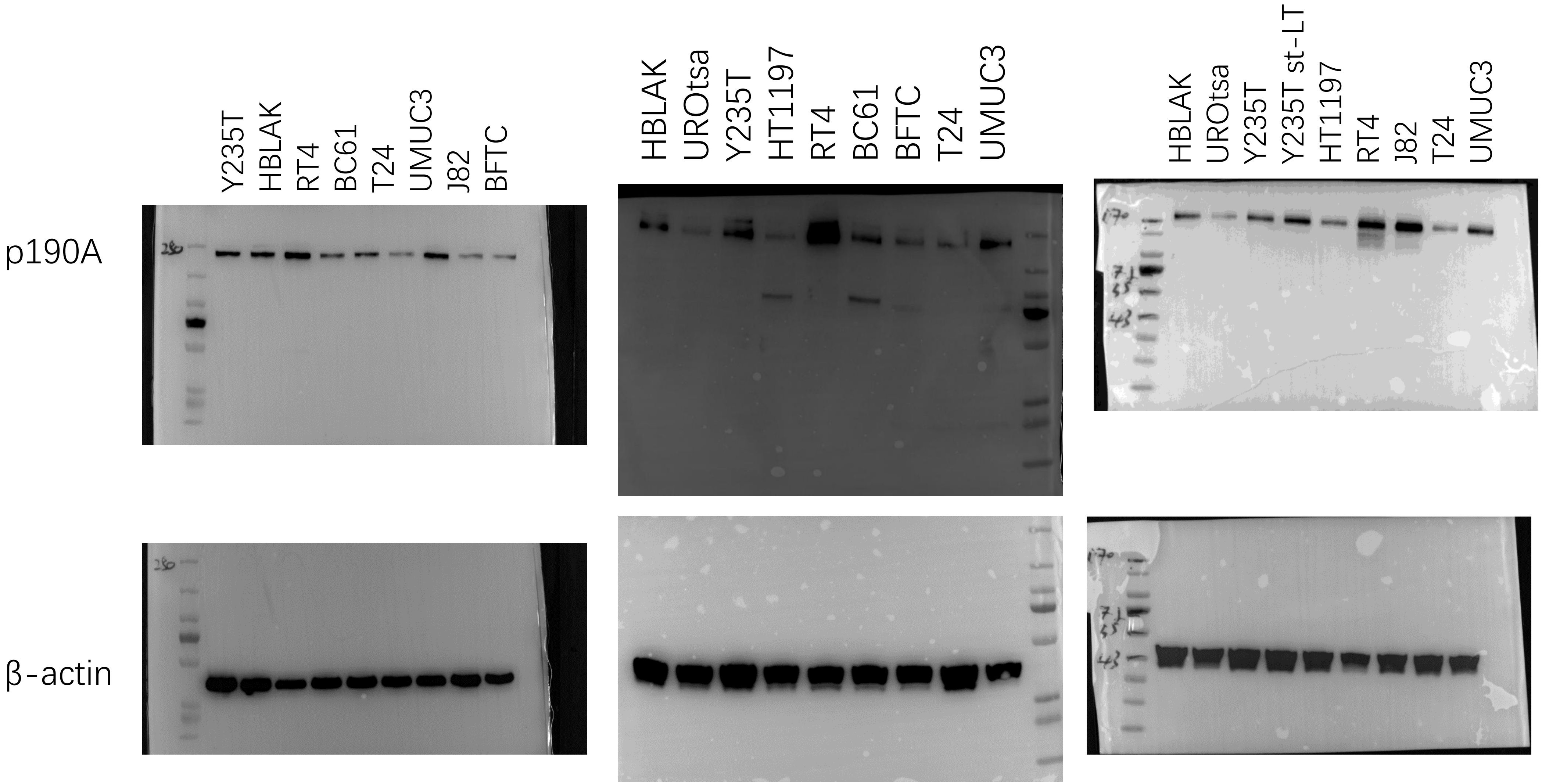

Figure 3B-RT4

p190A

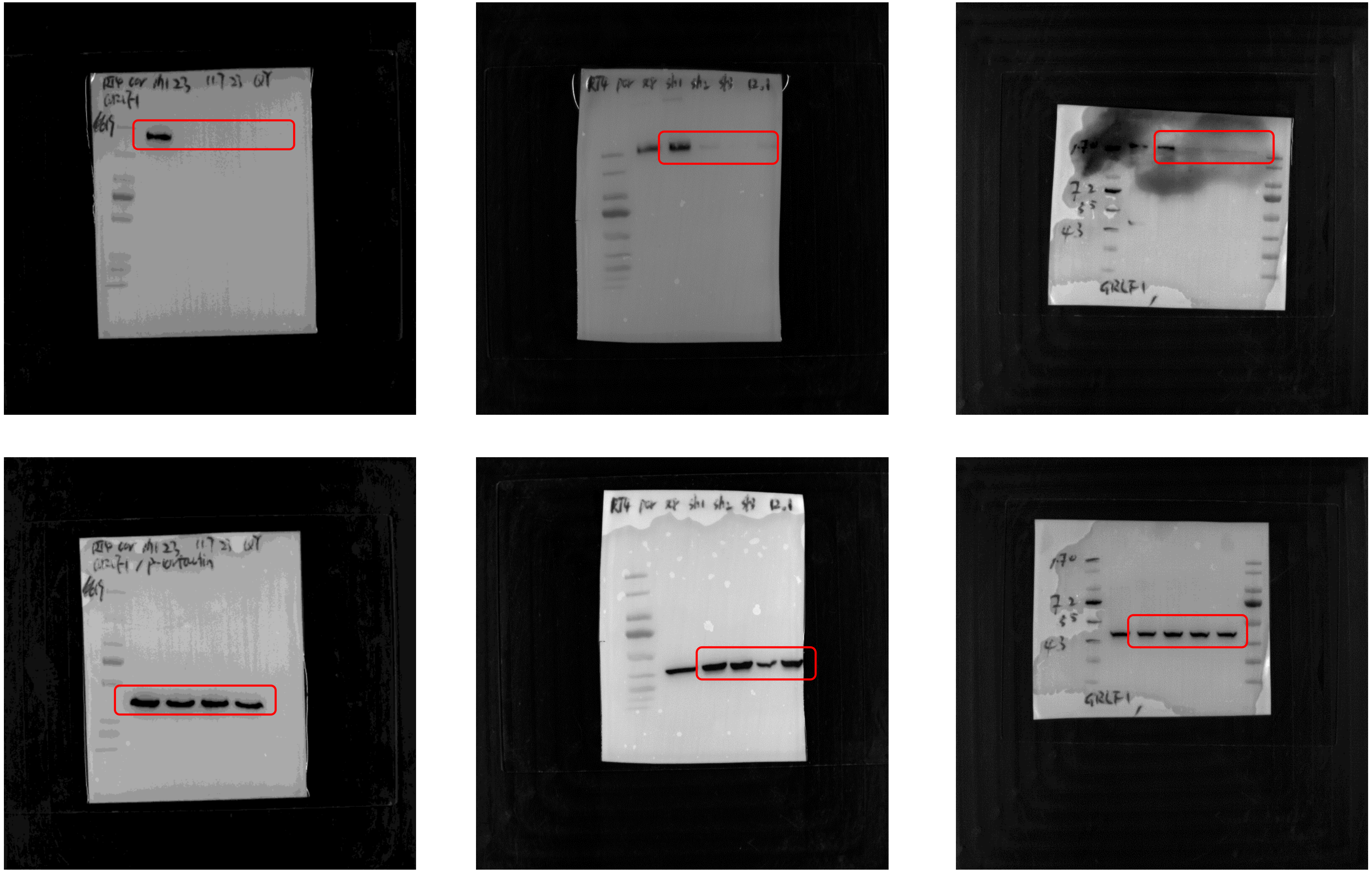

β-actin GAPDH β-actin

Figure 3C-BFTC

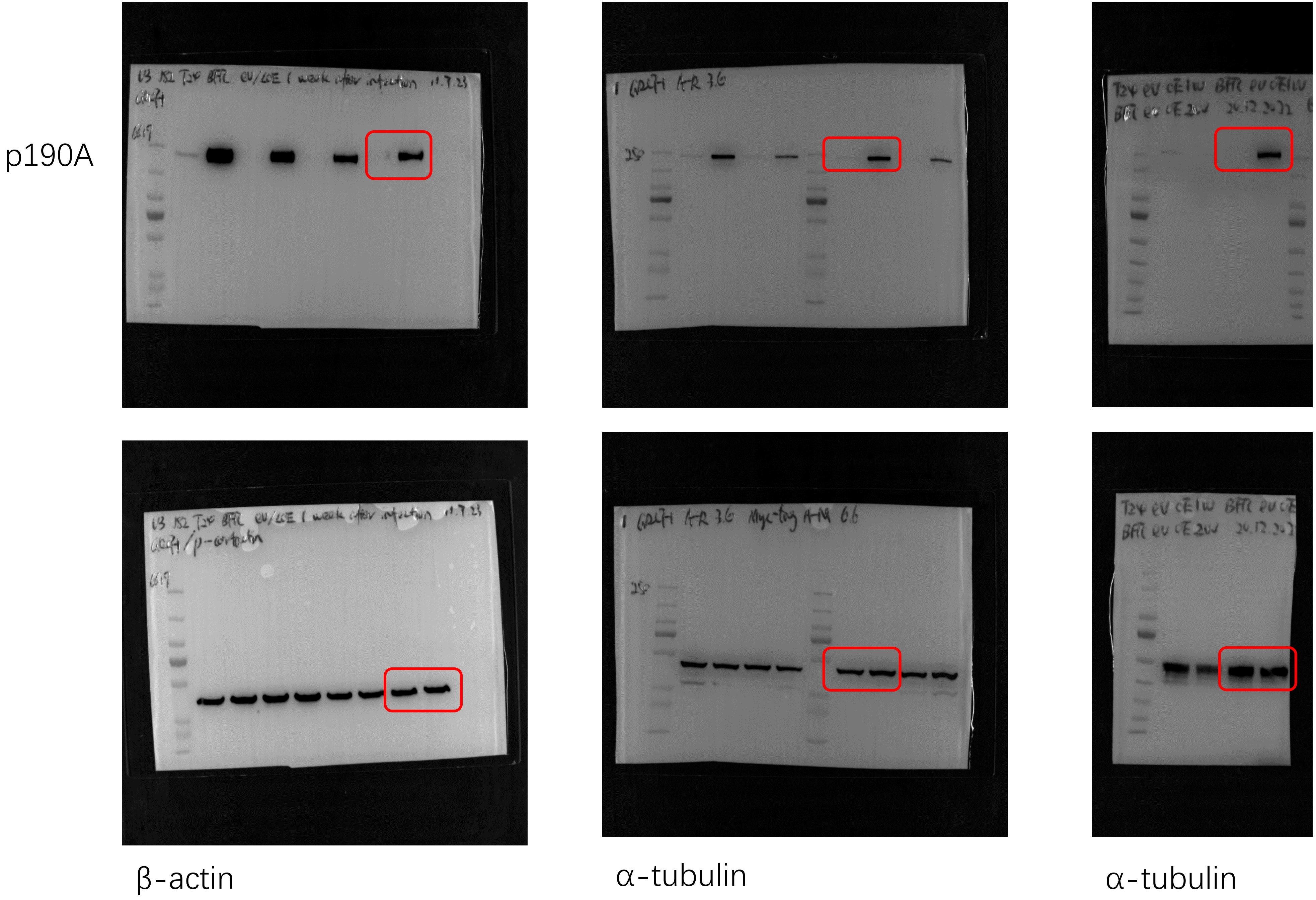

Figure 3D-T24

p190A

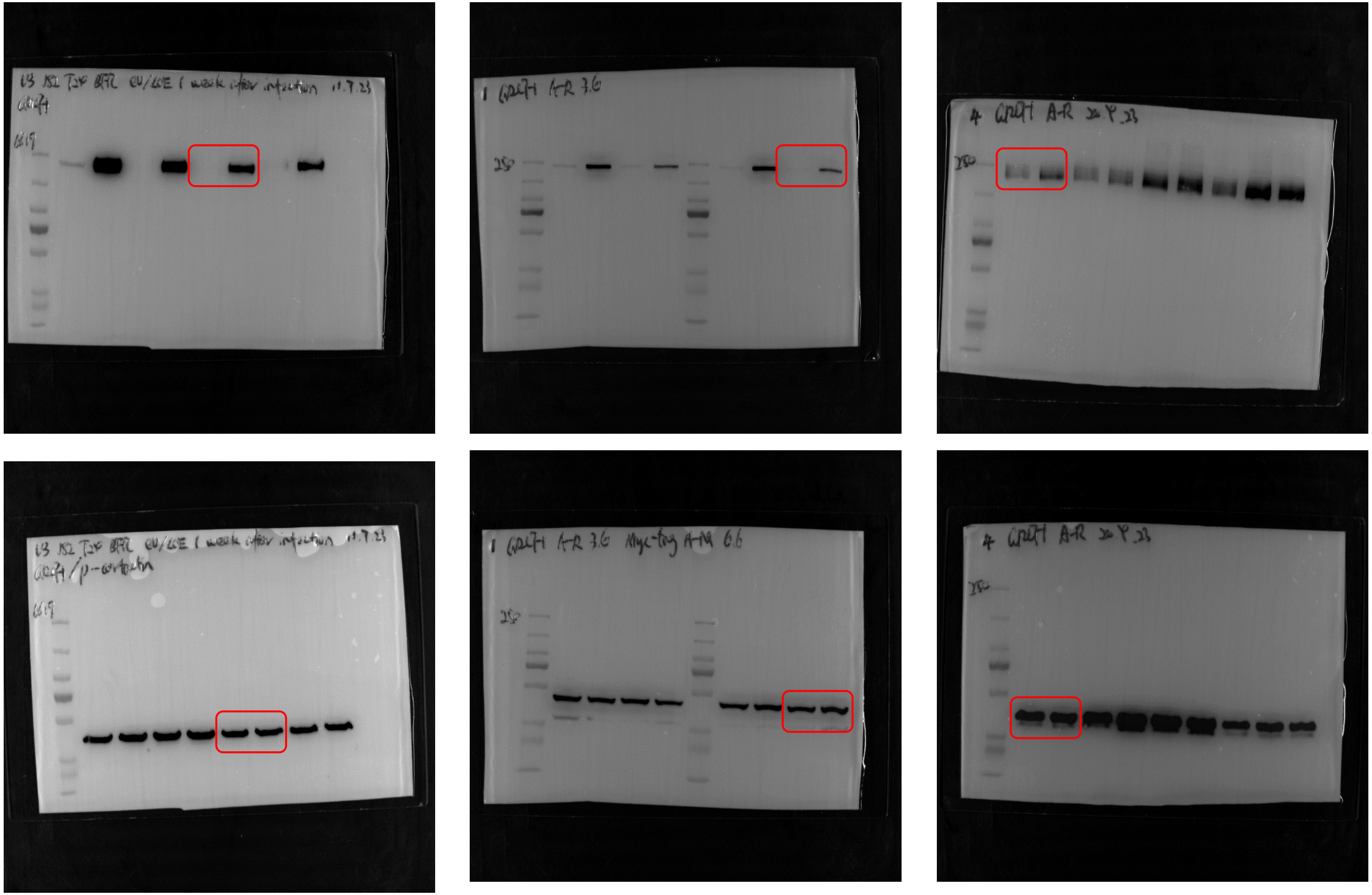

β-actin α-tubulin β-actin

Figure 5

Figure 5A-p190A

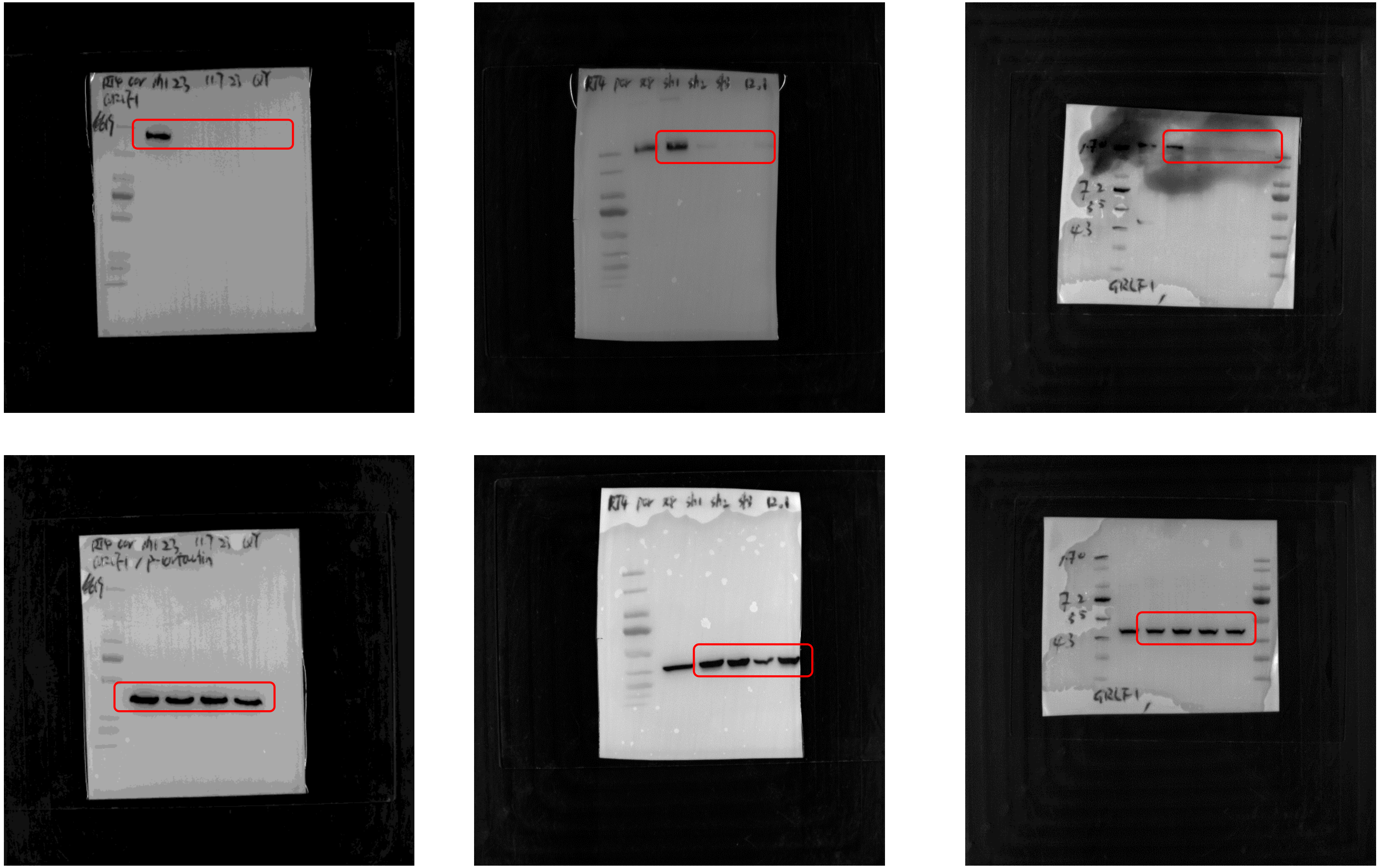

β-actin

GAPDH

β-actin

Figure 5A-RhoA

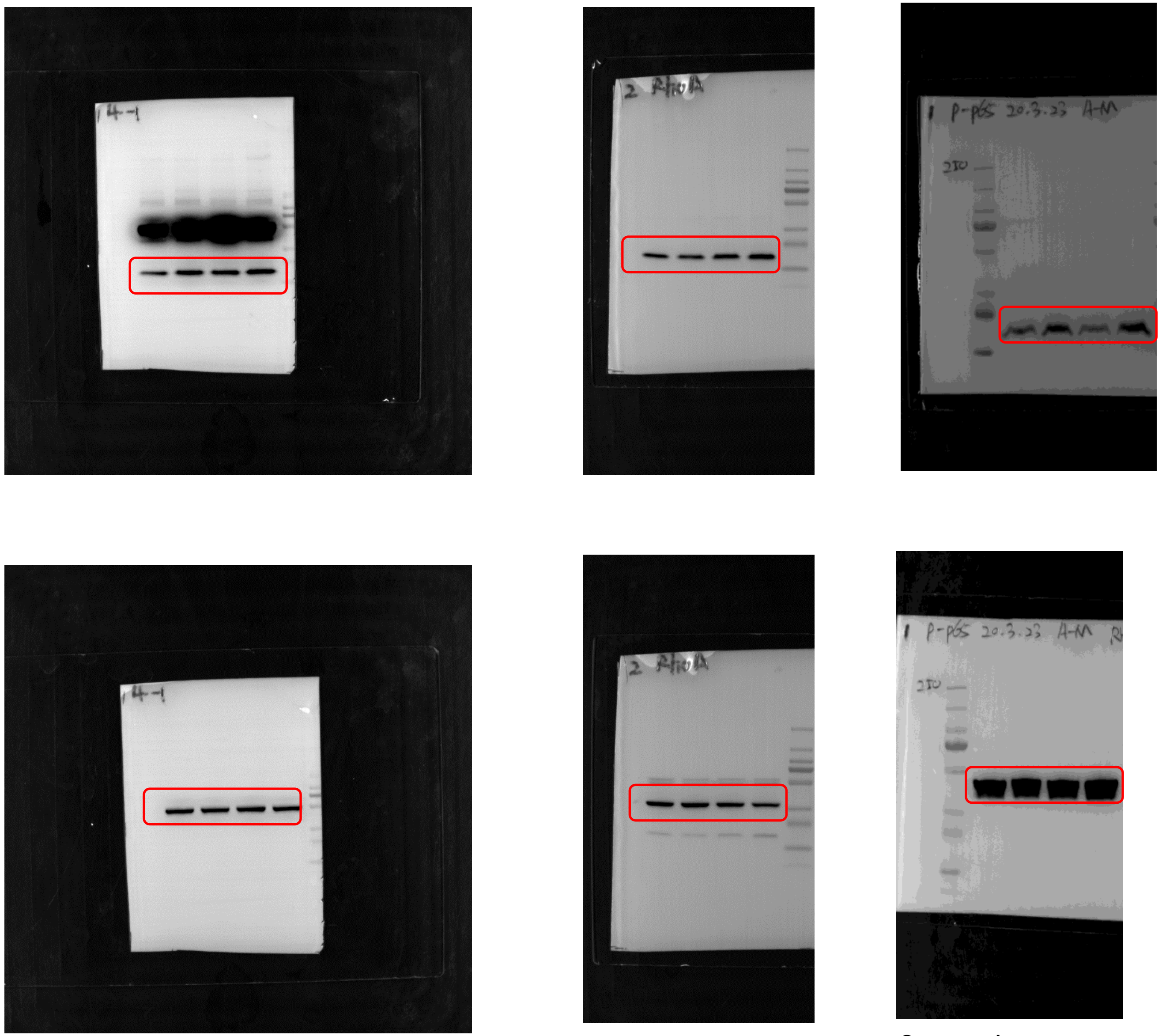

α-tubulin GAPDH β-actin

Figure 5A-ROCK1

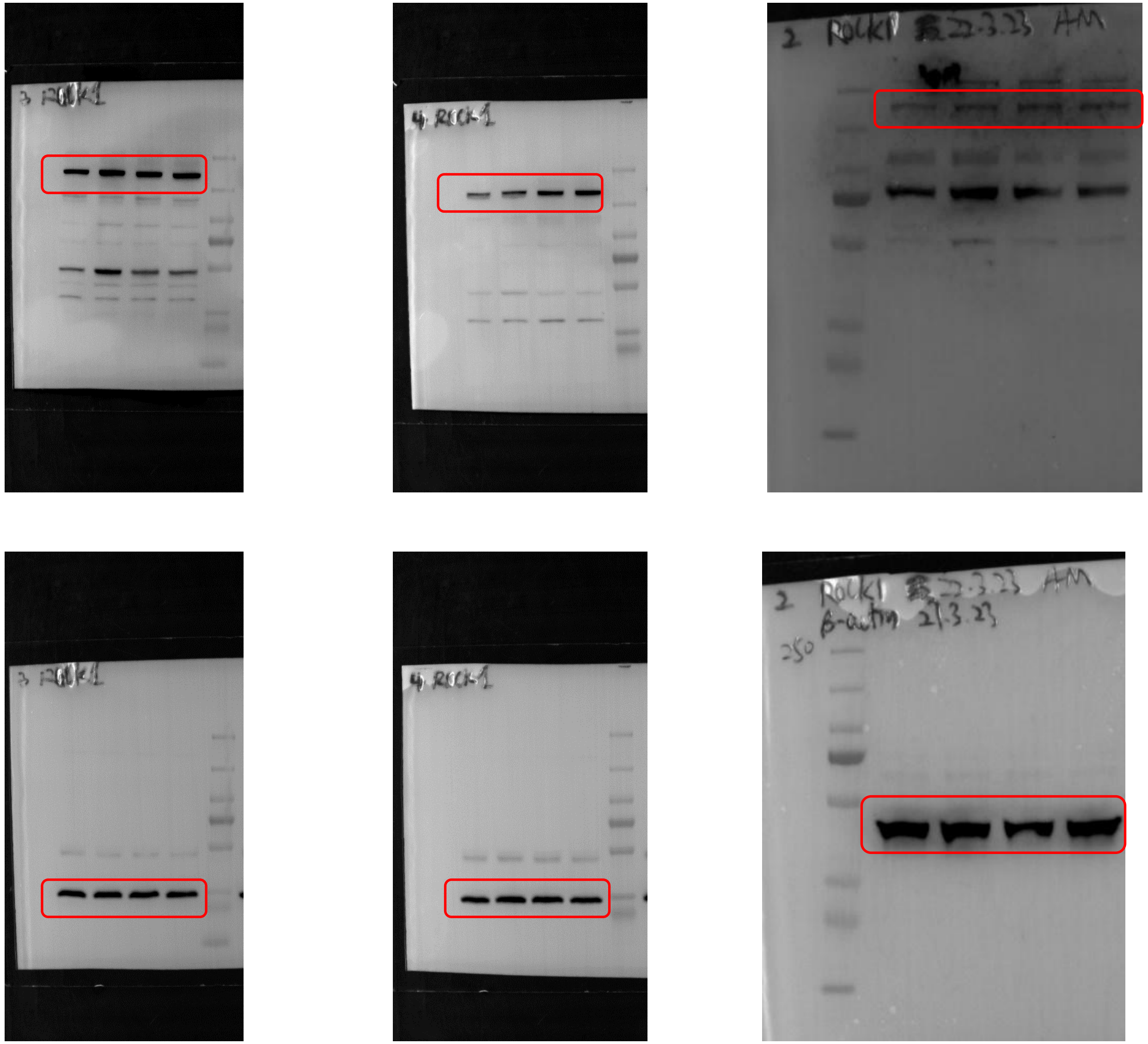

GAPDH GAPDH β-actin

Figure 5A-LIMK

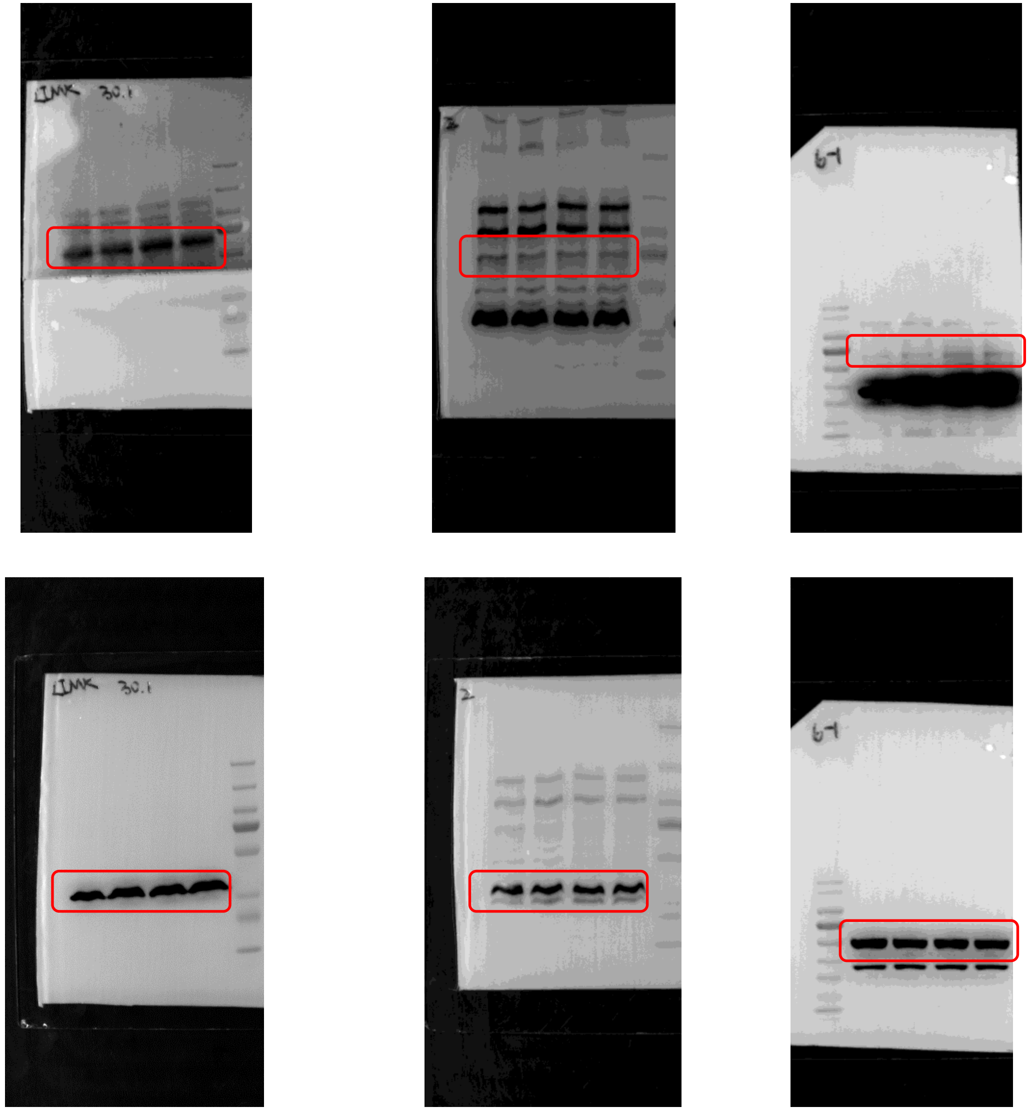

GAPDH GAPDH α-tubulin

Figure 5A-p-LIMK1/2

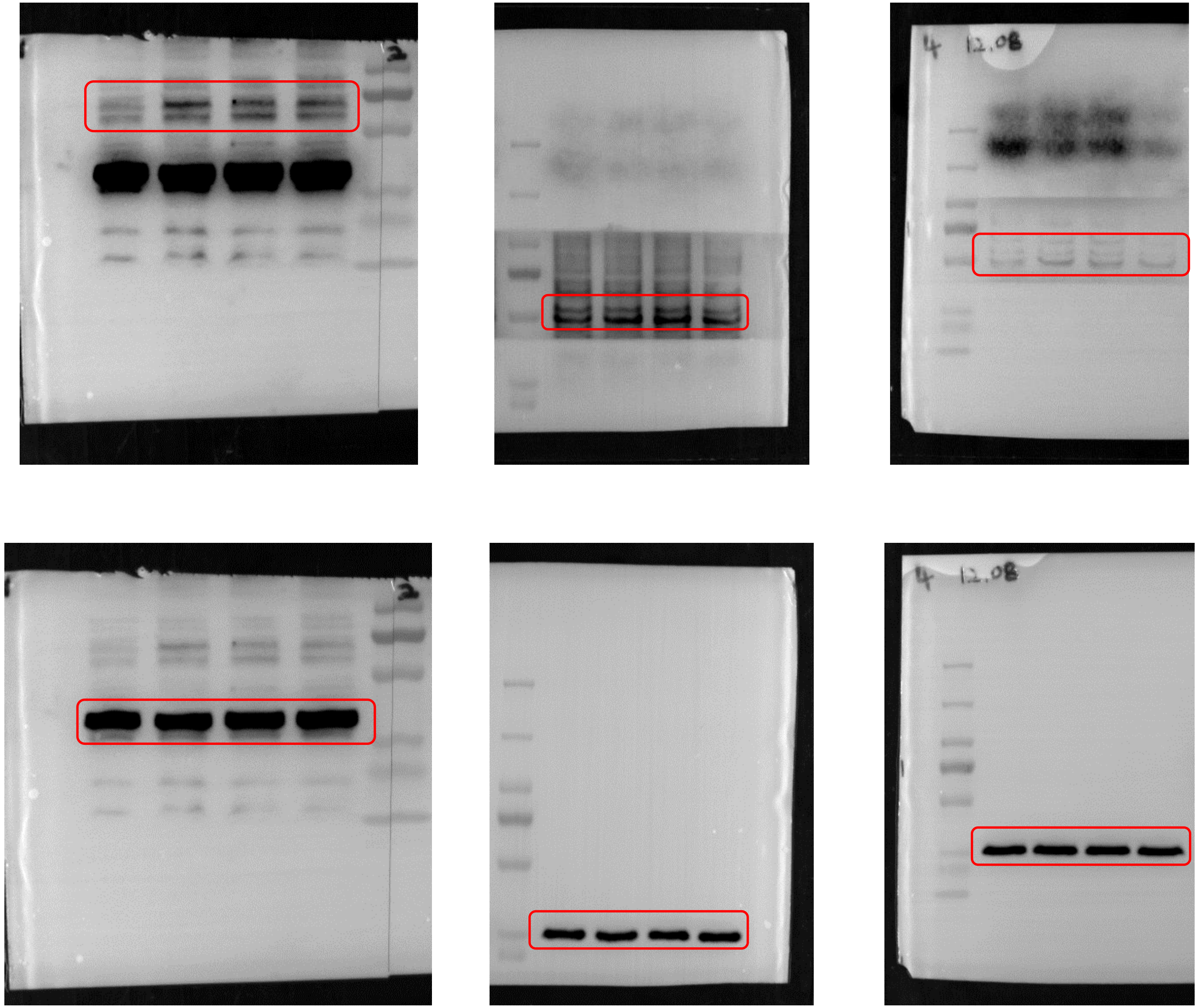

β-actin GAPDH GAPDH

Figure 5A-cofilin

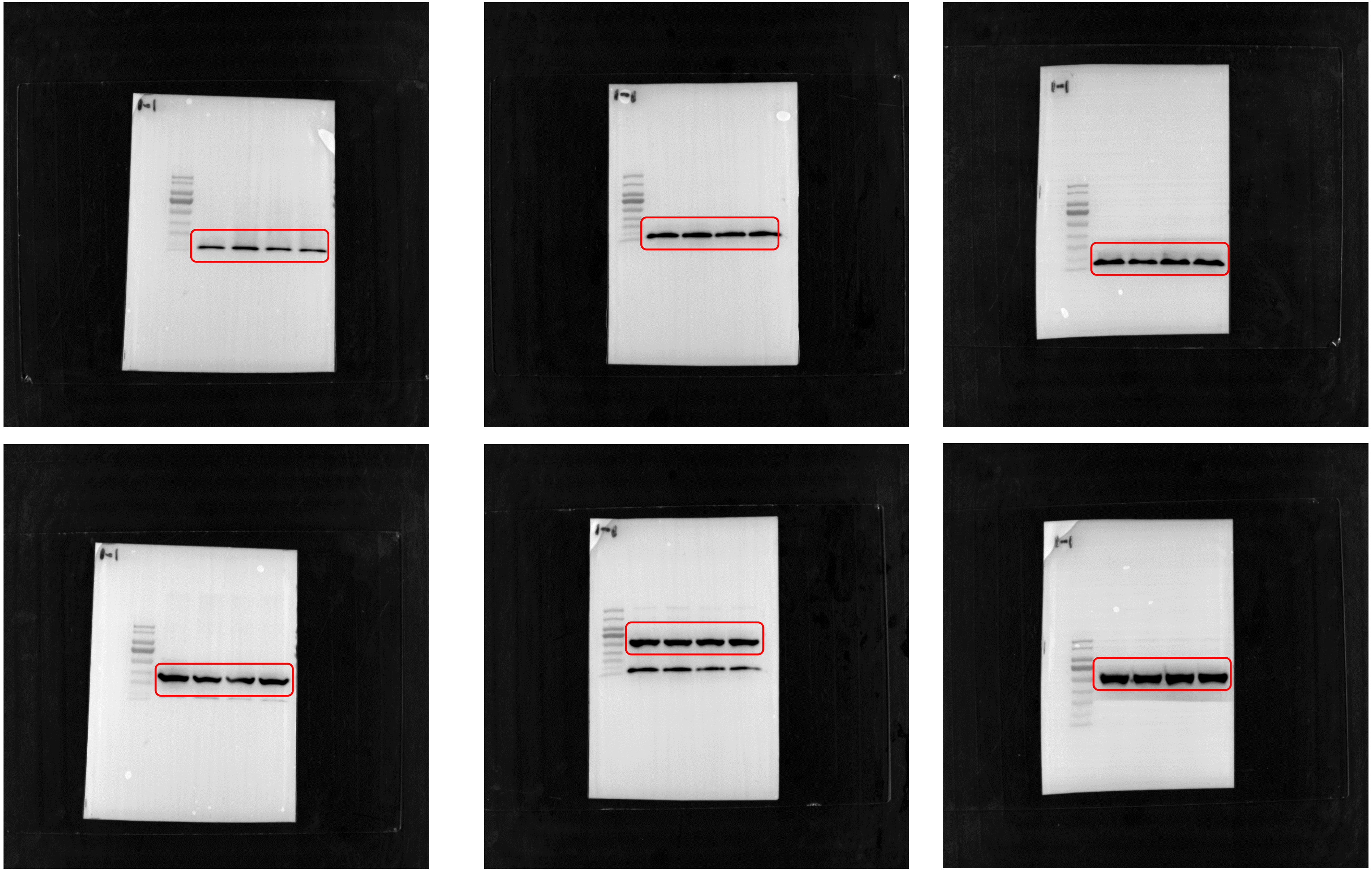

GAPDH α-tubulin α-tubulin

Figure 5A-p-cofilin1

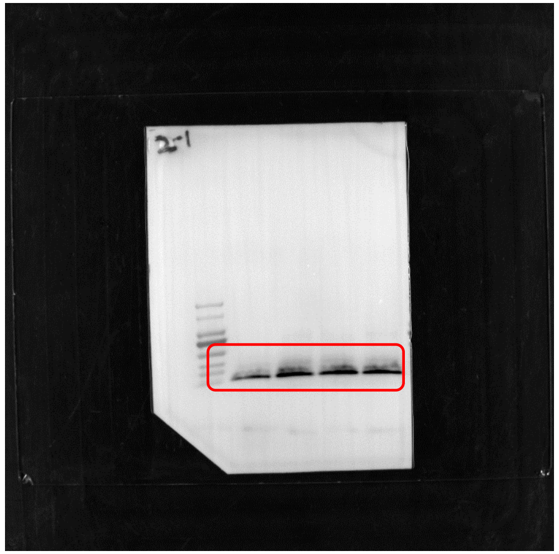

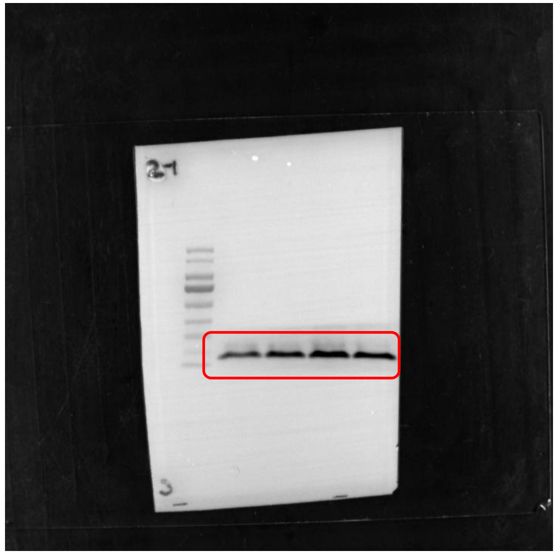

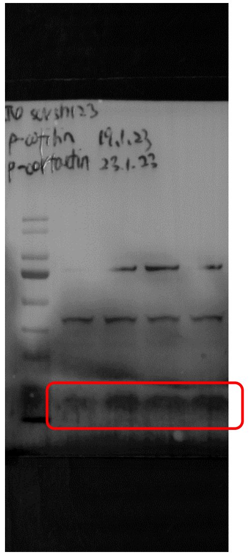

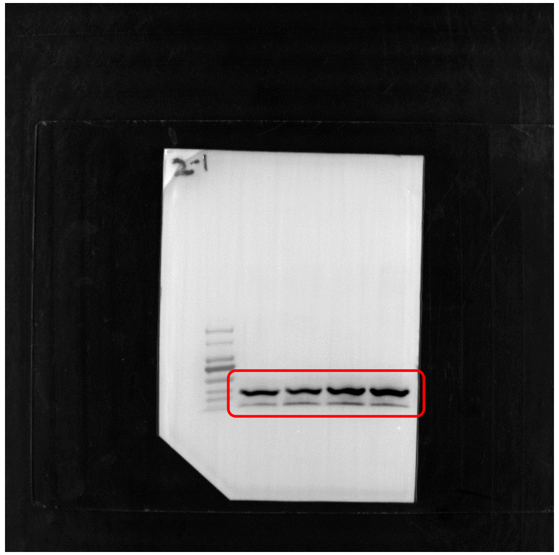

GAPDH

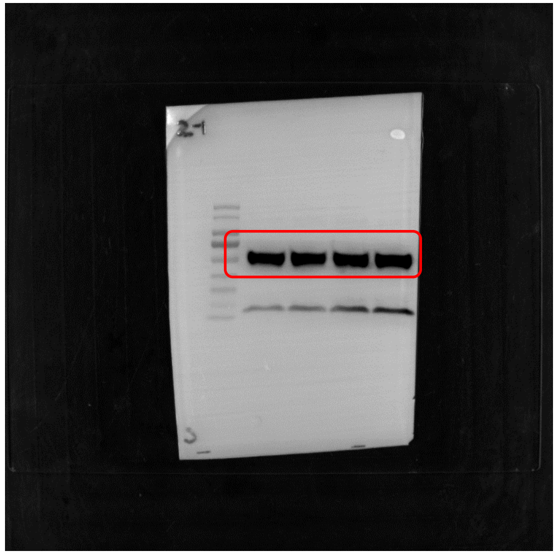

α-tubulin

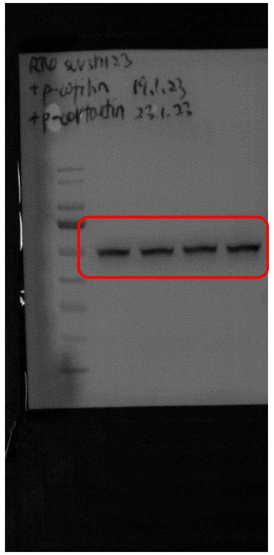

α-tubulin

Figure 5A-FAK

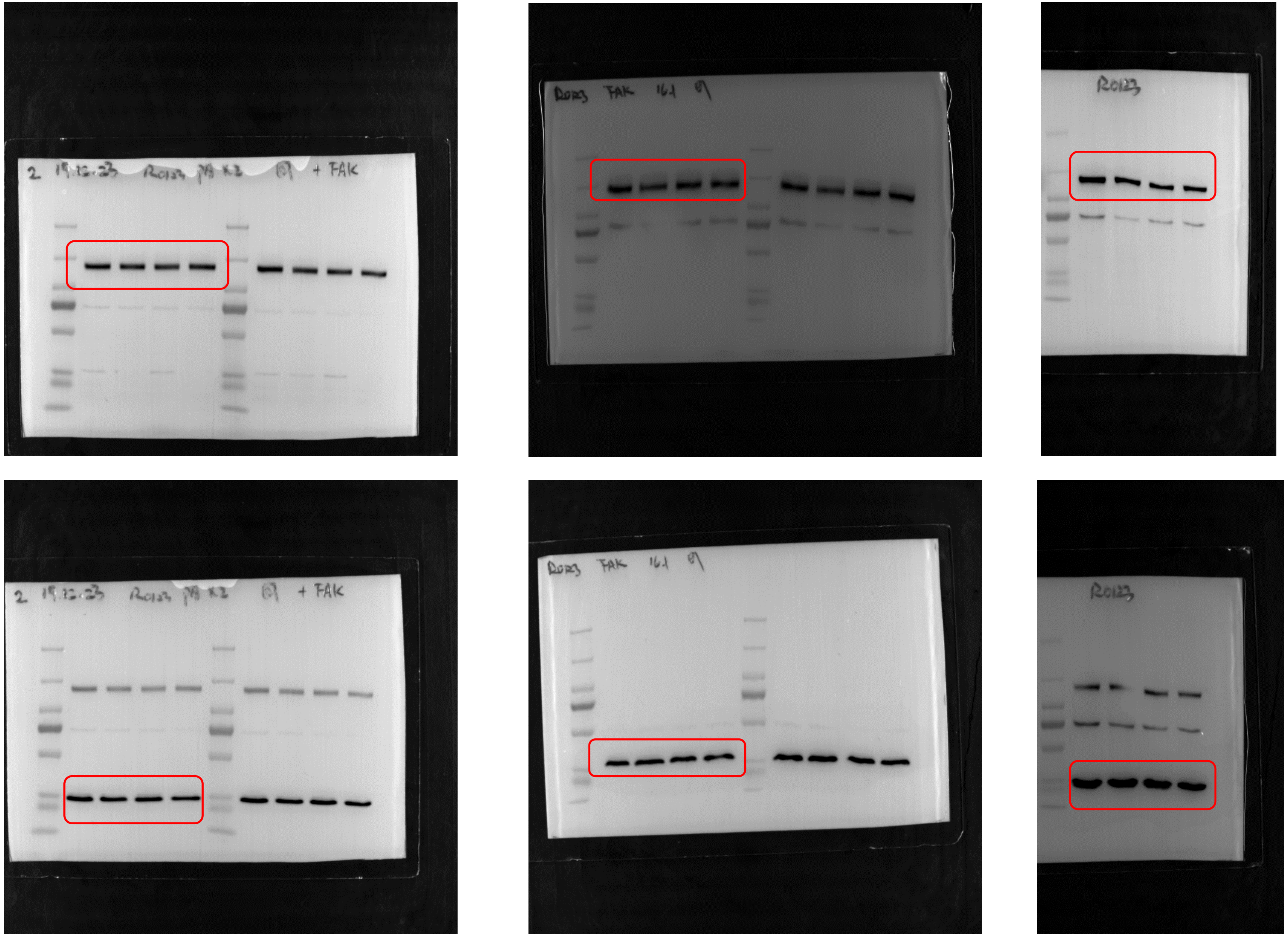

GAPDH GAPDH GAPDH

Figure 5A-p-FAK

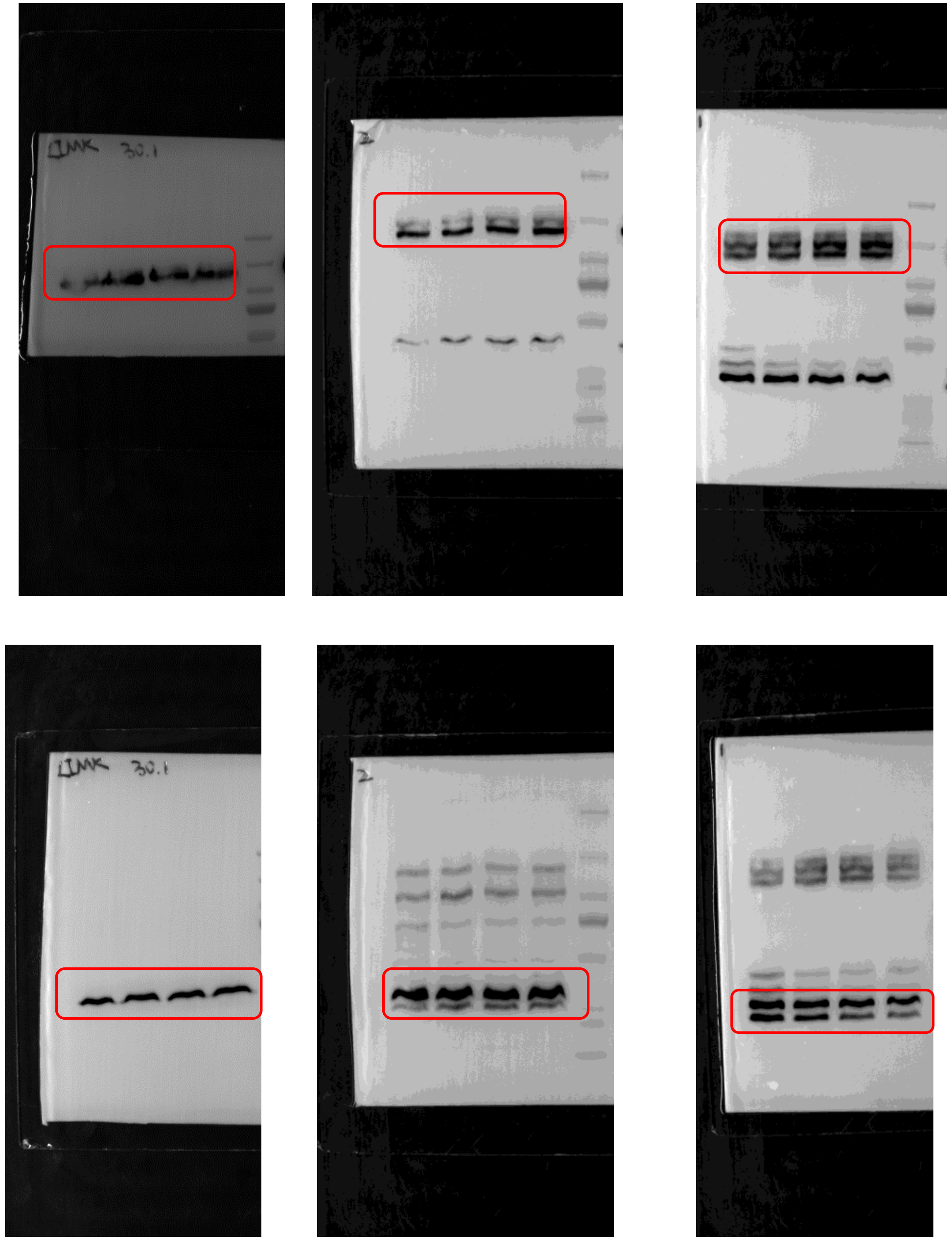

GAPDH GAPDH GAPDH

Figure 5A-cortactin

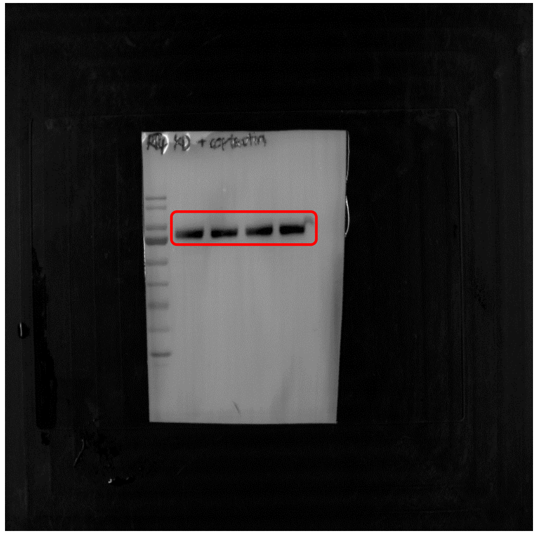

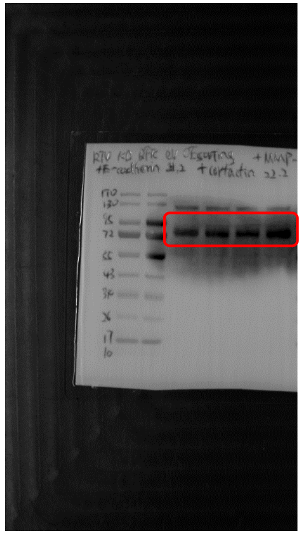

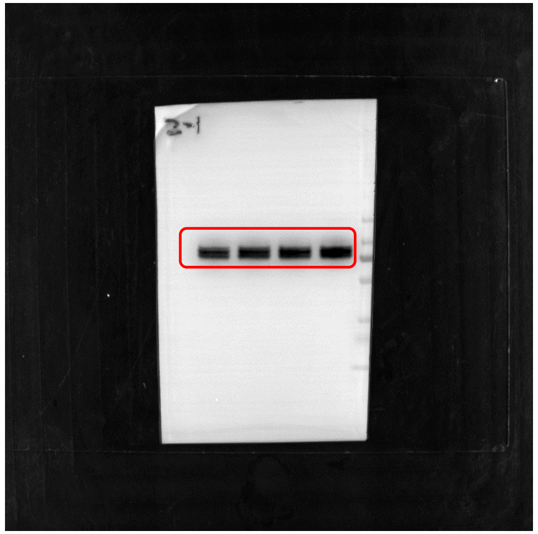

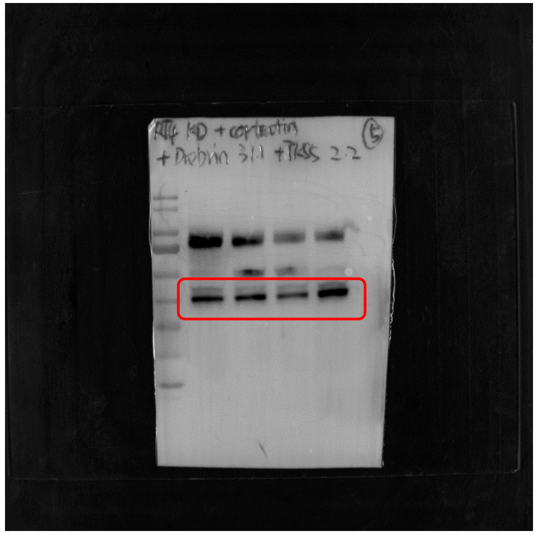

β-actin

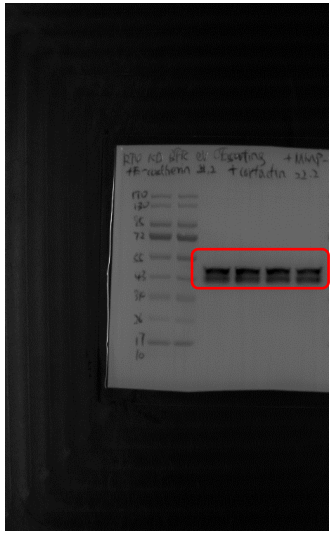

β-actin

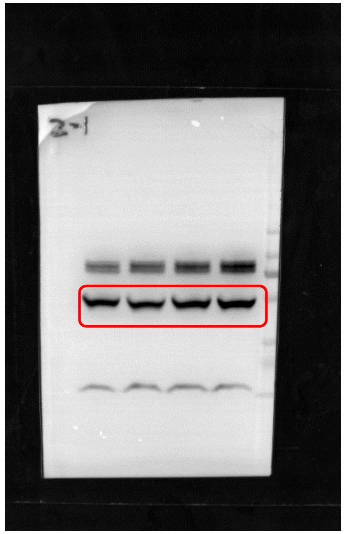

α-tubulin

Figure 5A-p-cortactin

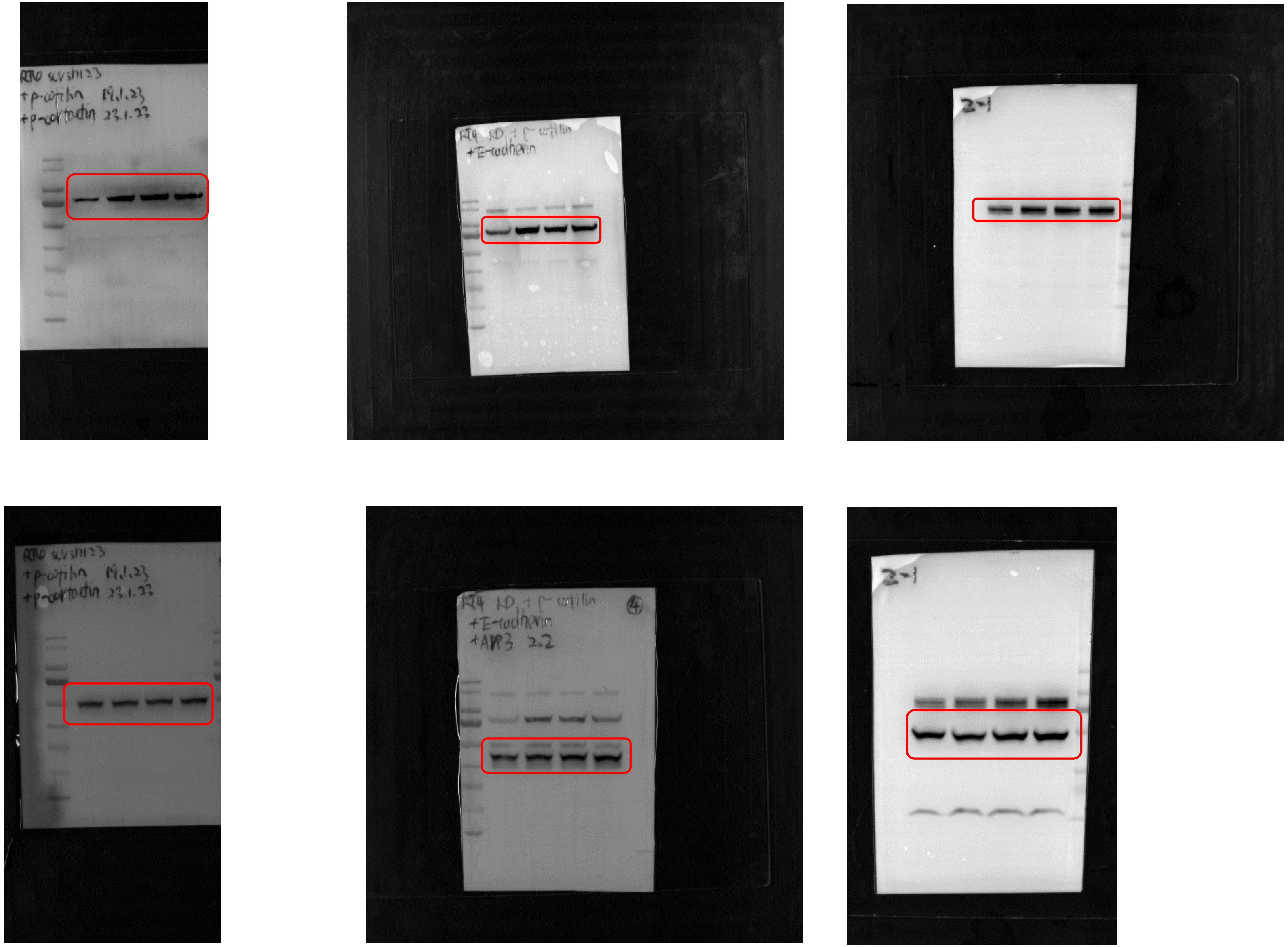

α-tubulin β-actin α-tubulin

Figure 5B-p190A

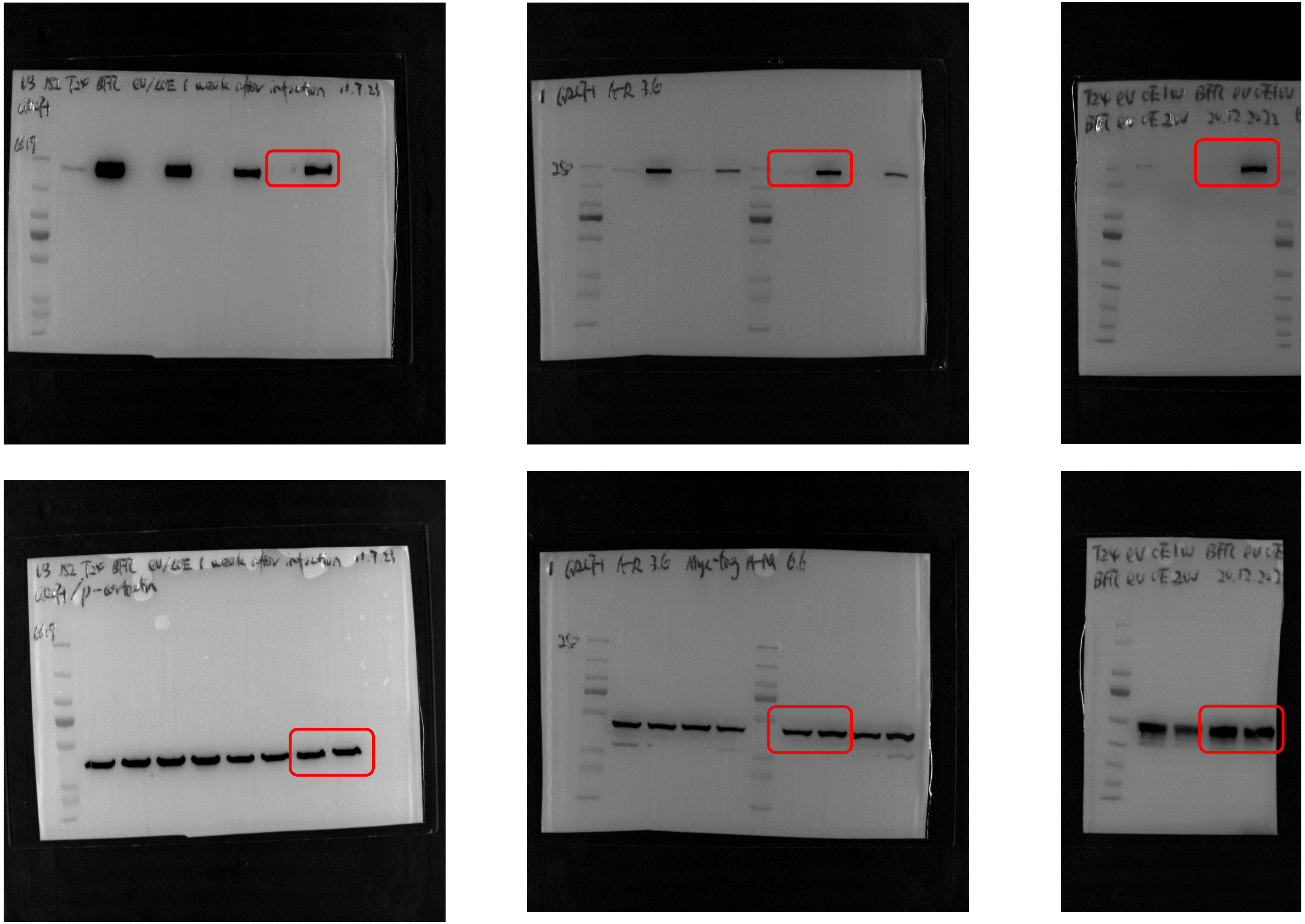

β-actin α-tubulin α-tubulin

Figure 5B-RhoA

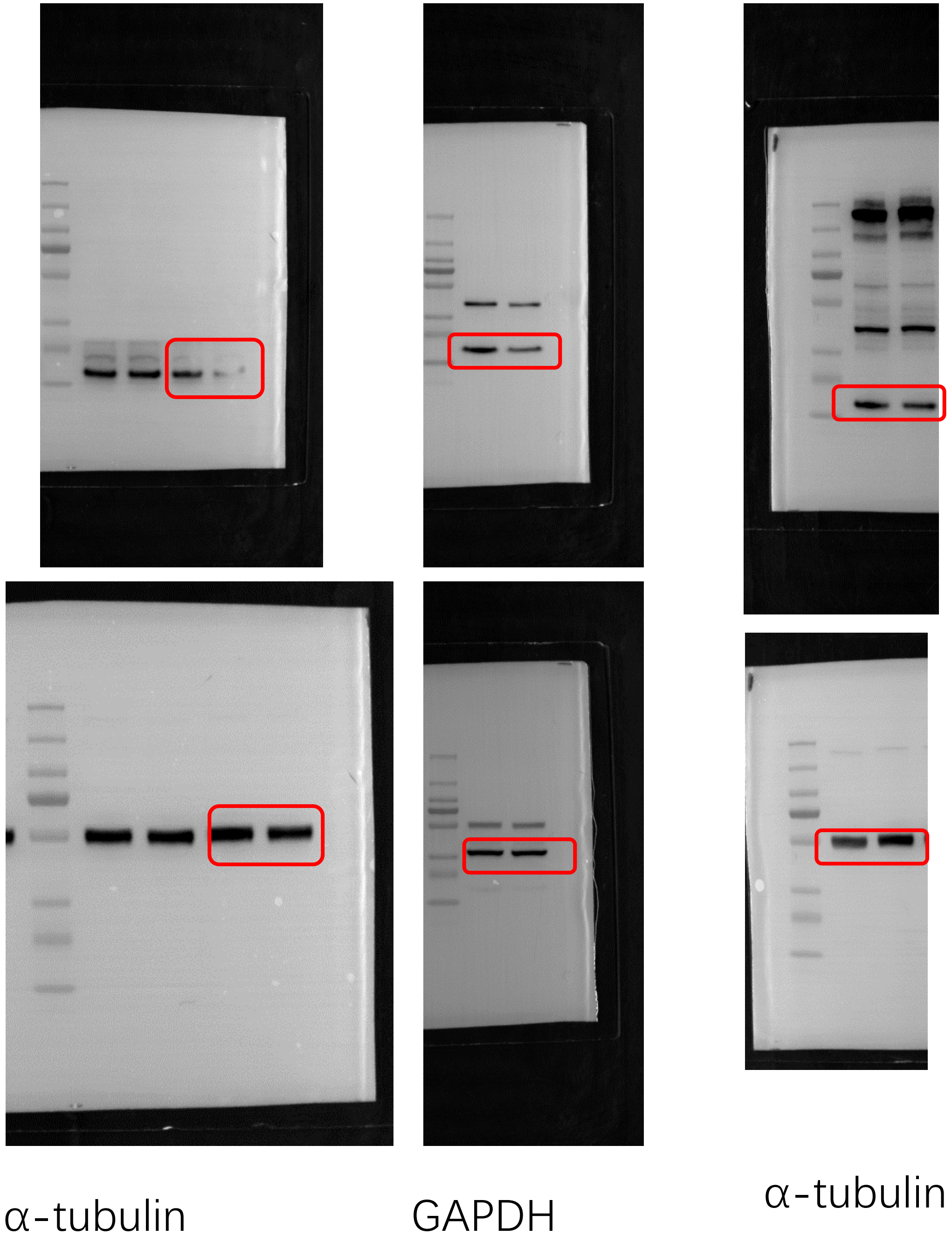

Figure 5B-ROCK1

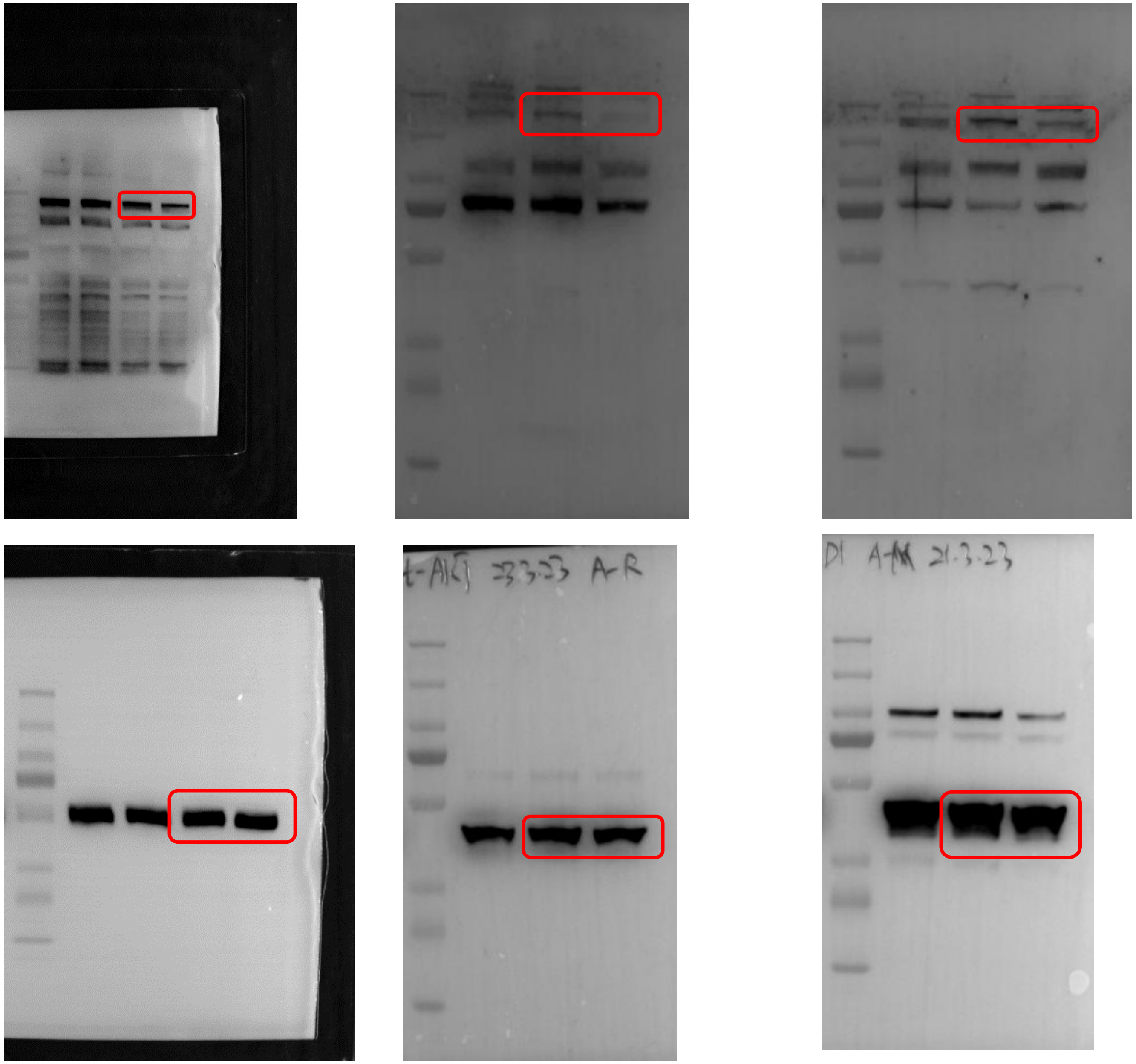

α-tubulin β-actin β-actin

Figure 5B-LIMK

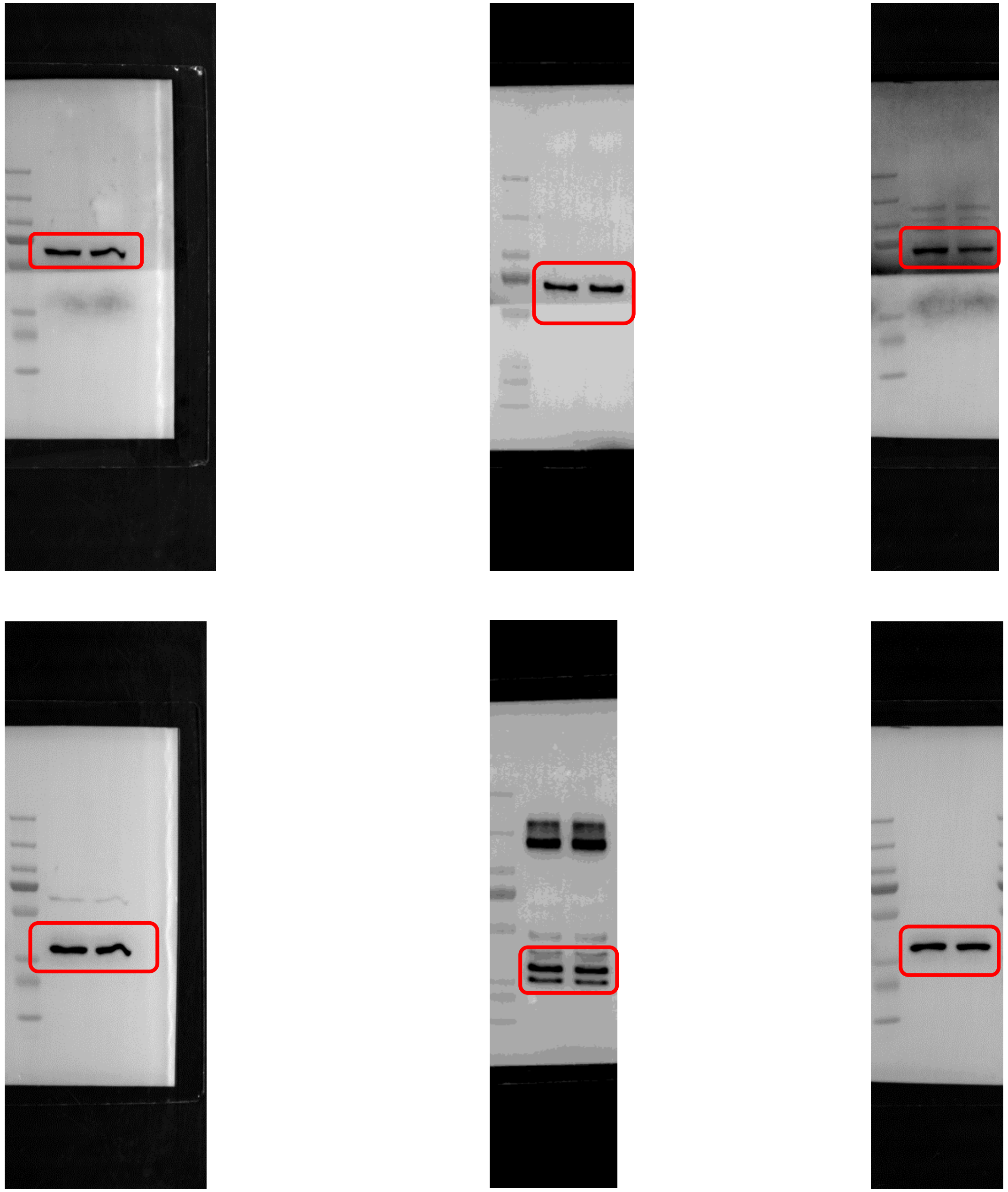

GAPDH GAPDH GAPDH

Figure 5B-p-LIMK

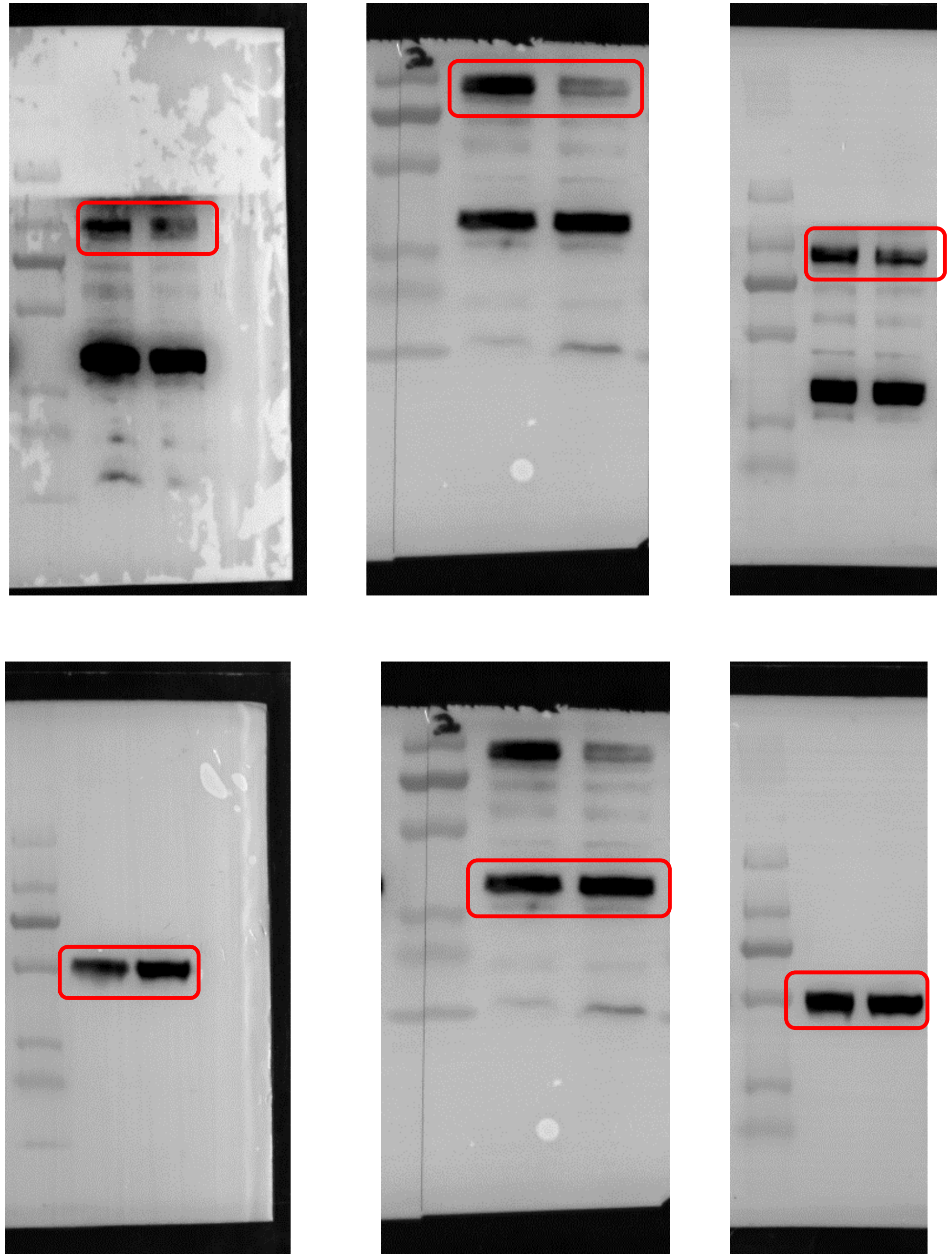

α-tubulin GAPDH α-tubulin

Figure 5B-cofilin

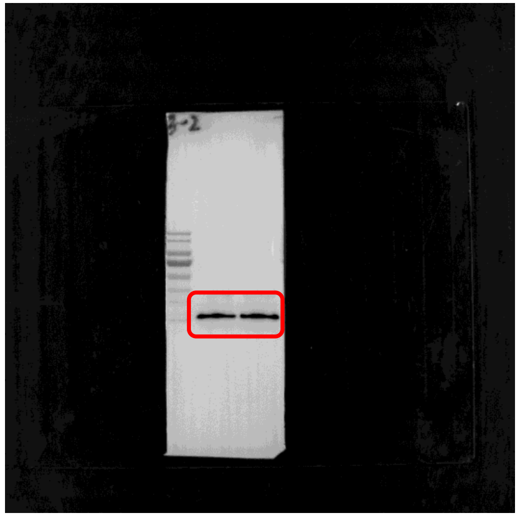

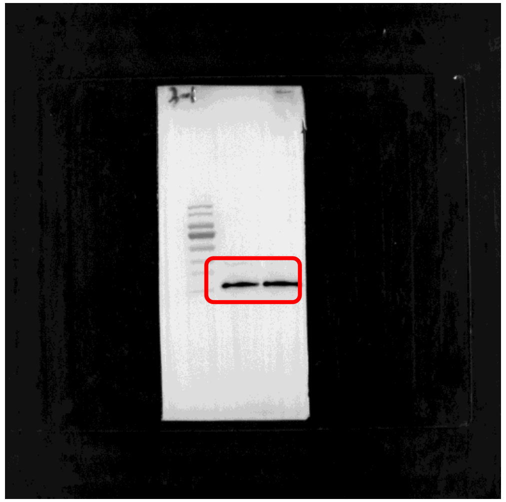

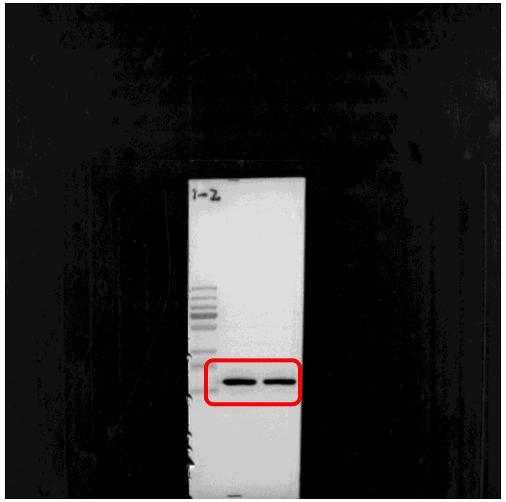

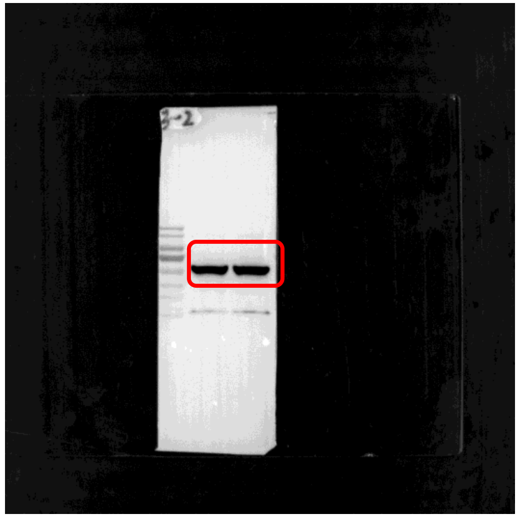

α-tubulin

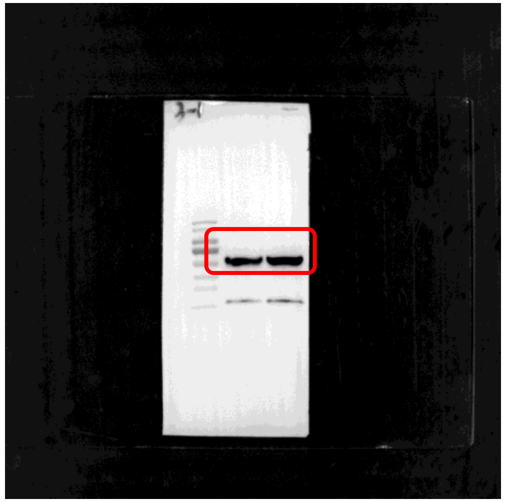

α-tubulin

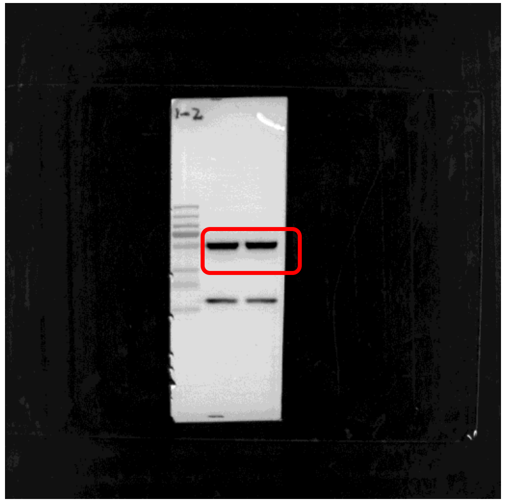

α-tubulin

Figure 5B-p-cofilin

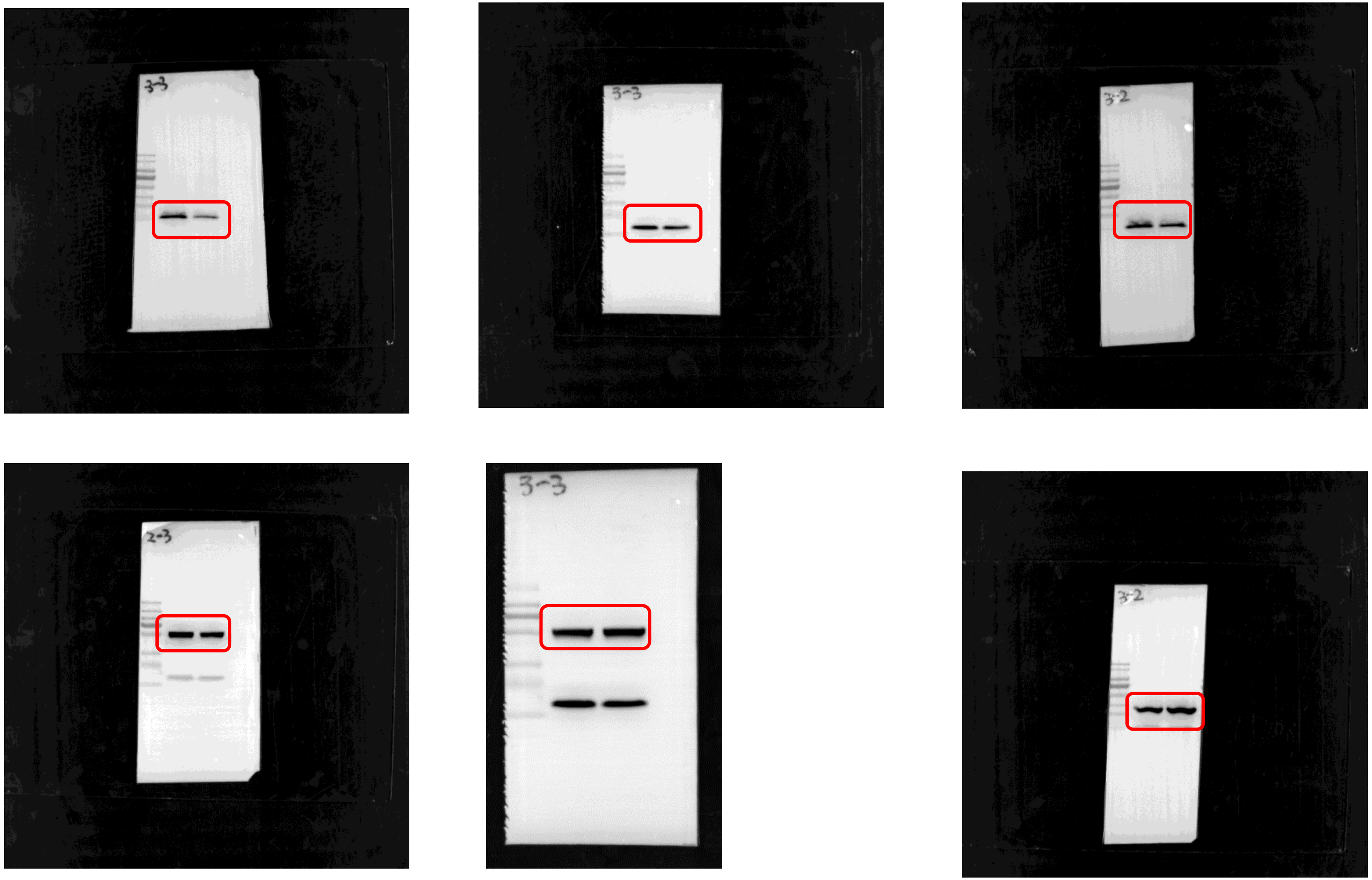

α-tubulin   α-tubulin   GAPDH

Figure 5B-FAK

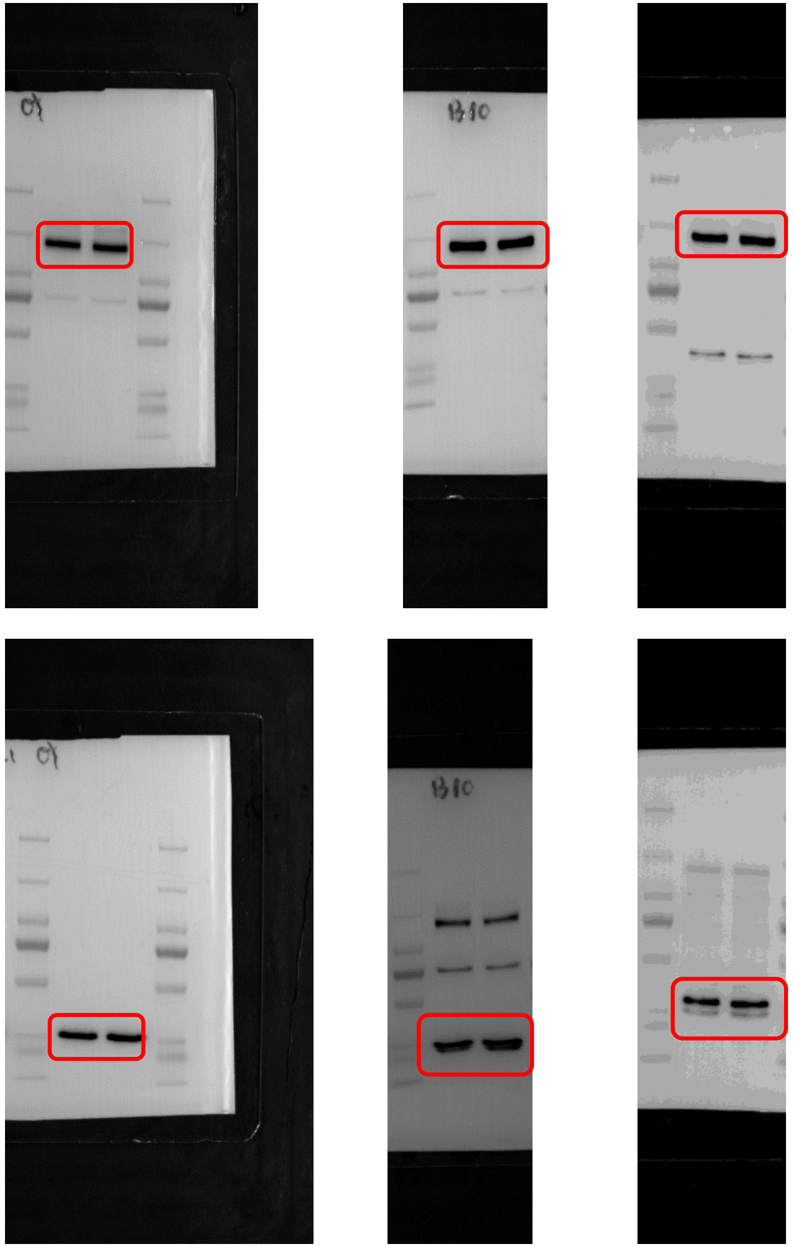

GAPDH GAPDH GAPDH

Figure 5B-p-FAK

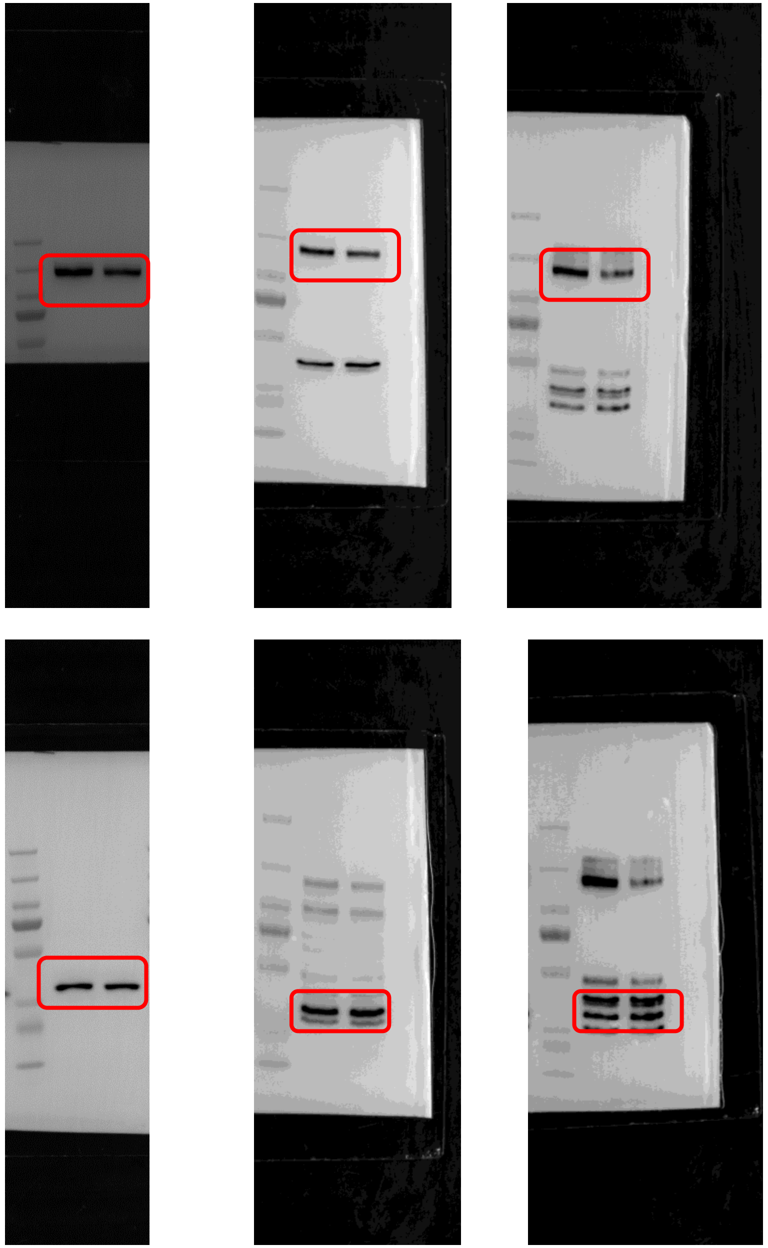

GAPDH GAPDH GAPDH

Figure 5B-cortactin

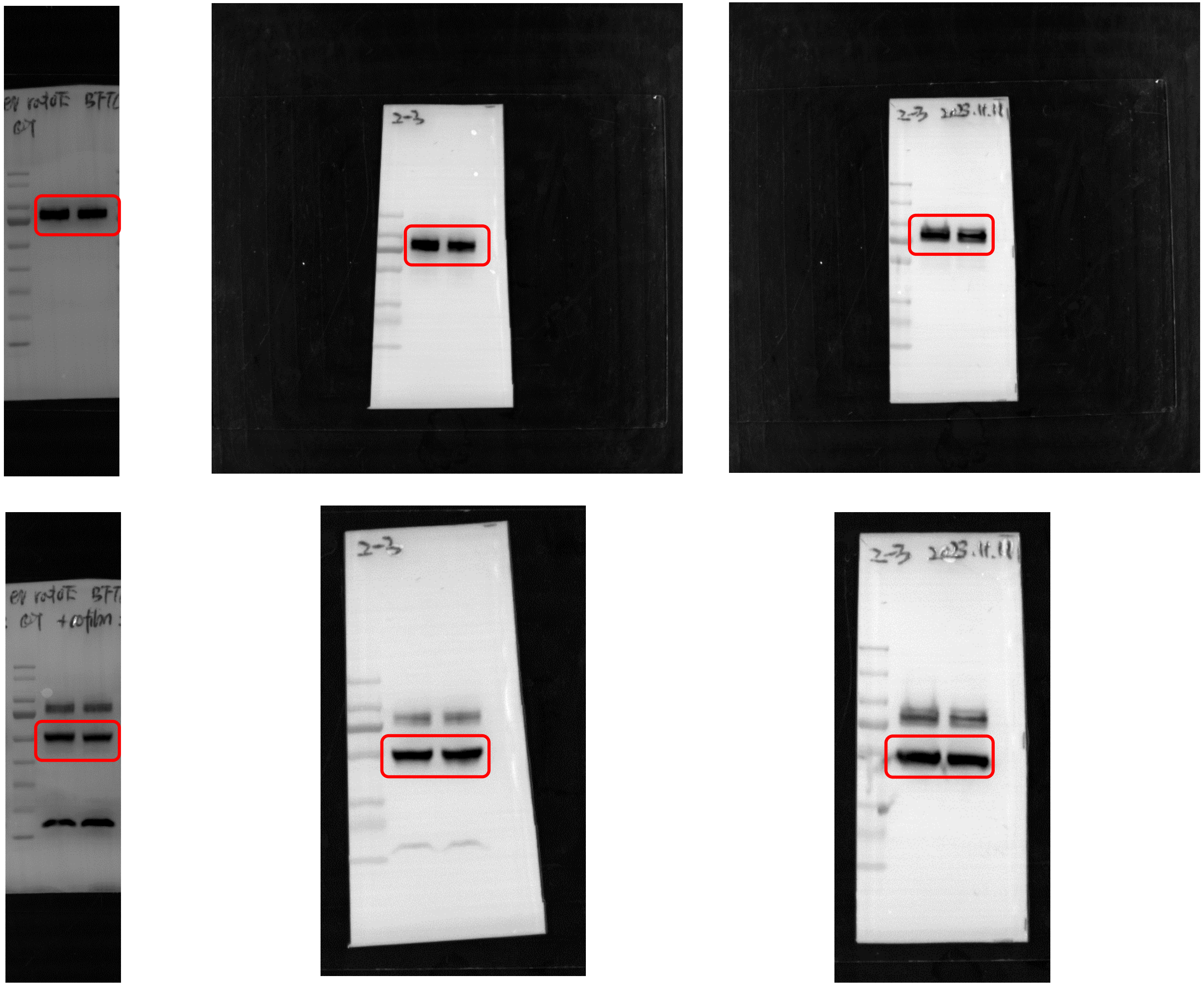

α-tubulin α-tubulin α-tubulin

Figure 5B-p-cortactin

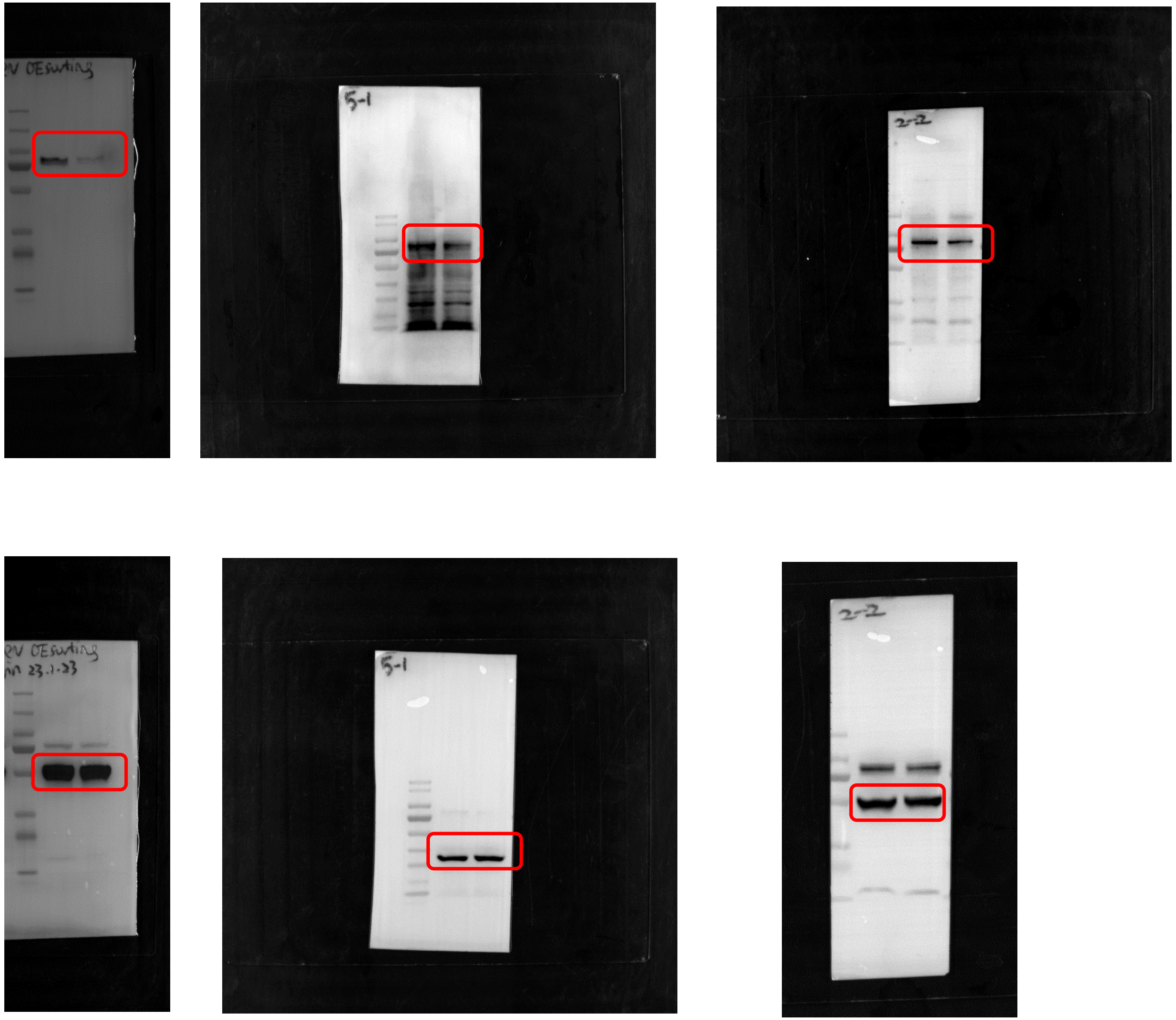

α-tubulin GAPDH α-tubulin
