# Supplementary material for: Loss of p190A RhoGAP induces aneuploidy and enhances bladder cancer cell migration and invasion by modulating actin dynamics
Source: Sci Rep. 2025 Nov 18;15:40399. doi: 10.1038/s41598-025-23687-4 (PMC12627482; doi:10.1038/s41598-025-23687-4)
Supplement: Supplementary file 8 — Supplementary Material 8 [file 41598_2025_23687_MOESM8_ESM.pdf]

Supplementary Tables

Table S1. Clinical data of patient with MIBC

**Total**	**152**
**OS**	
dead	132
Unknown	20
**RFS**	
Recurrence	78
No recurrence	54
Unknown	20
**pT stage**	
pT2	29
pT3	66
pT4	37
Unknown	20
**pN stage**	
pN0	82
PN1	11
pN2	28
pNX	11
Unknown	20
**WHO grading**	
high grade	132
low grade	0
Unknown	20
**Gender**	
Male	92
Female	40
Unknown	20

**Age**	
37-59	25
60-79	81
80-91	26
Unknown	20
**Histology types**	
Neuroendocrine	2
Urothelial	130
Unknown	20
**MDACC subtype**	
Basal	53
DN	4
Luminal	50
Unkown	45
**Variant Histology**	
Glandular	2
Glycogen rich	1
LNUC	5
Lymphoepithelioma-like	2
MPUC	8
Neuroendocrine	4
NOS	55
PUC	4
Sarcomatoid	15
Squamous	36
Unknown	20

Table S2. Staining intensity scores

Staining intensity (SI)	Score
no color detectable	0
low staining intensity	1
moderate color intensity	2
strong staining intensity	3

Table S3. Number of positive cells scores

Number of positive cells (PP)	Score
no positive detectable	0
less than 10 % positive cells	1
10-50 % positive cells	2
51-80 % positive cells	3
more than 80 % positive cells	4

Calculation of the Immunoreactive score (IRS) = SI x PP
